# Synthetic biology as driver for the biologization of materials sciences

**DOI:** 10.1016/j.mtbio.2021.100115

**Published:** 2021-05-26

**Authors:** O. Burgos-Morales, M. Gueye, L. Lacombe, C. Nowak, R. Schmachtenberg, M. Hörner, C. Jerez-Longres, H. Mohsenin, H.J. Wagner, W. Weber

**Affiliations:** a École Supérieure de Biotechnologie de Strasbourg - ESBS, University of Strasbourg, Illkirch, 67412, France; b Faculty of Biology, University of Freiburg, Freiburg, 79104, Germany; c Signalling Research Centres BIOSS and CIBSS, University of Freiburg, Freiburg, 79104, Germany; d Spemann Graduate School of Biology and Medicine - SGBM, University of Freiburg, Freiburg, 79104, Germany; e Department of Biosystems Science and Engineering - D-BSSE, ETH Zurich, Basel, 4058, Switzerland

**Keywords:** Interactive materials, Engineered living materials, Metabolic engineering, Cell encapsulation, Nanomaterials, Smart materials, Stimulus-responsive materials

## Abstract

Materials in nature have fascinating properties that serve as a continuous source of inspiration for materials scientists. Accordingly, bio-mimetic and bio-inspired approaches have yielded remarkable structural and functional materials for a plethora of applications. Despite these advances, many properties of natural materials remain challenging or yet impossible to incorporate into synthetic materials. Natural materials are produced by living cells, which sense and process environmental cues and conditions by means of signaling and genetic programs, thereby controlling the biosynthesis, remodeling, functionalization, or degradation of the natural material. In this context, synthetic biology offers unique opportunities in materials sciences by providing direct access to the rational engineering of how a cell senses and processes environmental information and translates them into the properties and functions of materials. Here, we identify and review two main directions by which synthetic biology can be harnessed to provide new impulses for the biologization of the materials sciences: first, the engineering of cells to produce precursors for the subsequent synthesis of materials. This includes materials that are otherwise produced from petrochemical resources, but also materials where the bio-produced substances contribute unique properties and functions not existing in traditional materials. Second, engineered living materials that are formed or assembled by cells or in which cells contribute specific functions while remaining an integral part of the living composite material. We finally provide a perspective of future scientific directions of this promising area of research and discuss science policy that would be required to support research and development in this field.

## Graphical abstract

**Figure:**
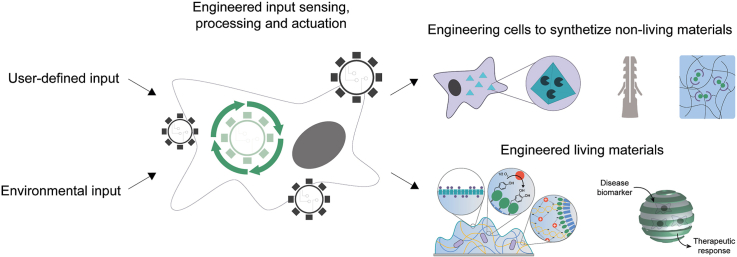

## 1 Introduction

The development of new materials has been a key driver of major changes in human history. Many of the periods of humanity, from the Stone Age to the Bronze Age and Iron Age, are classified by innovations in materials sciences. A major breakthrough in materials sciences of our era was the development of plastics, which are now ubiquitous in all areas of life but which also pose an enormous environmental burden. However, despite such unceasing progress in materials sciences, our ability to synthesize and engineer materials still falls short when it comes to synthesizing materials that can recapitulate the properties and functions of natural materials. Valuable properties of materials in nature include programmability [1,2], multifunctionality [3], or the ‘self’-properties such as self-growth [4], self-adaptivity [5], self-assembly [4], or self-healing [6]. Furthermore, nature-made materials are synthesized from renewable resources in hierarchical structures, they harvest required energy directly from their environment and are, in a cradle-to-cradle circle, degraded to serve as raw blocks for other materials [7] — properties that are also desirable in modern human-made materials.

**Fig. 1:**
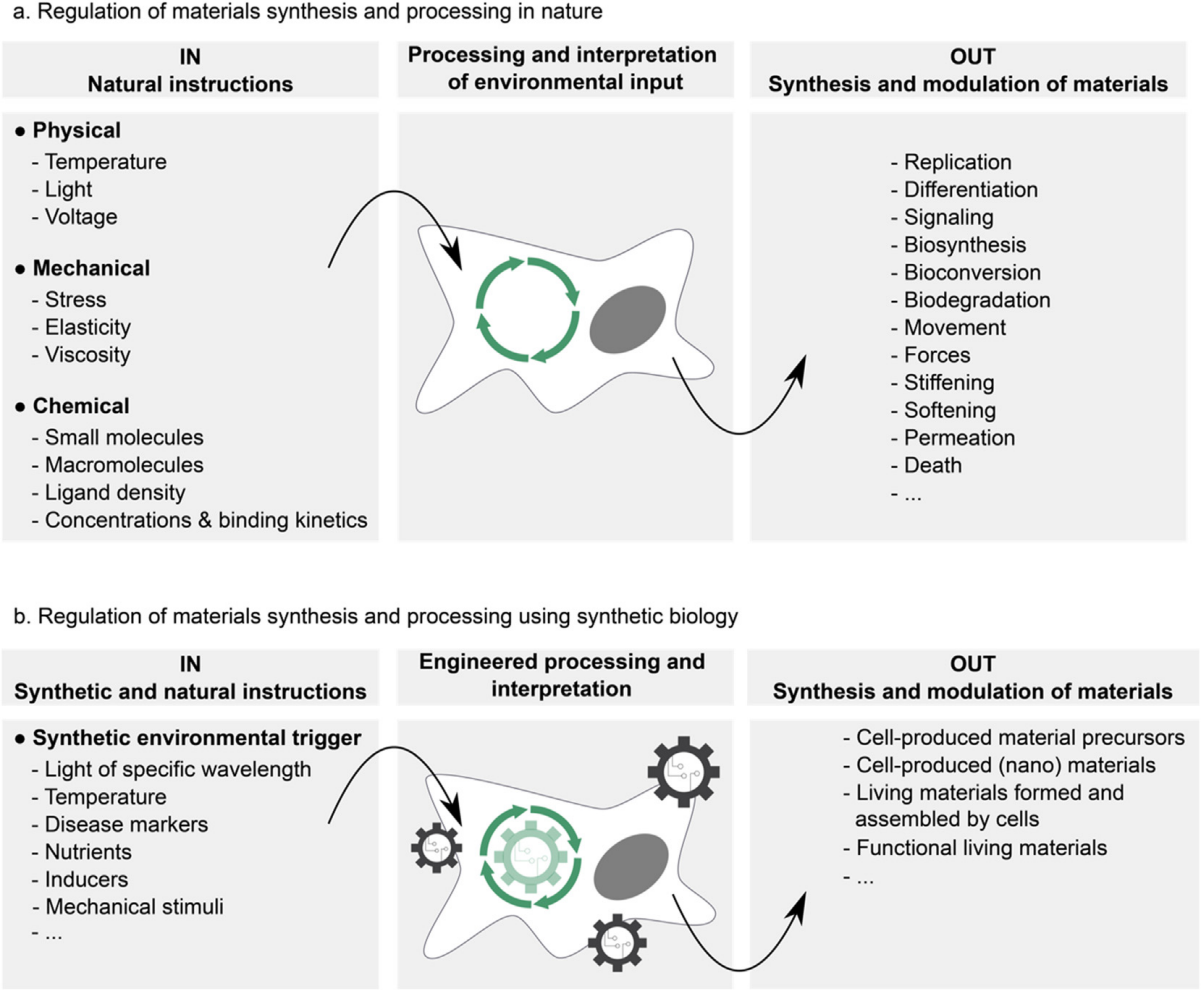
**Natural and engineered control of materials synthesis and processing by cells. a.** Situation in nature. Cells sense environmental parameters such as physical, mechanical, and chemical cues and integrate and process these inputs by cellular signaling networks. There, signals are, for example, amplified, dampened, integrated with each other, or stored to control the genetic, epigenetic, and metabolic programs of the cell. These programs subsequently determine the cellular activity to modulate the synthesis, processing, or function of the natural material. **b.** Synthetic biology approach. Using synthetic biology, the cellular signaling network is engineered. Synthetic and environmental input signals are wired to desired processing networks that subsequently control cellular output. Thus, cell fate and function are controlled to program the synthesis, processing, and the function of materials or their precursors.

The primordial synthesis machine of materials found in nature is the living cell. Organisms — whether unicellular or multicellular — have tailored their material synthesis capabilities to their needs and environment through billions of years of evolution. To achieve this, cells must be exquisitely attuned to their environment and respond appropriately. This process comprises three core steps (Fig. 1a): sensing the environment, processing the input information, and modulation of materials synthesis. Cells sense environmental parameters such as nutrient availability or sunlight, and mechanical inputs such as the viscoelasticity of their environment or externally applied forces. Furthermore, in multicellular communities and organisms, cells are embedded in an intercellular signaling network: neighbors interact with one another and more distant cells communicate through molecular or mechanical signals. The cell senses this multitude of input signals by receptors that transduce the information to intracellular signal processing. There, different signals are amplified, stored, or integrated with each other by layers of signaling processes involving kinases, phosphatases, second messengers, proteases, and other signal transducers that link the external information flow to metabolic, genetic, and epigenetic programs. Based on the outcome of such cellular signal processing, cells produce enzymes to synthesize, export, remodel, or degrade the material in the extracellular matrix. The cell may further change material properties by contraction and dilatation, or by stiffening and softening via modulation of its cytoskeleton. For example, a growing plant depends on the availability of light. Thus, when shade is perceived by photoreceptors, this information is relayed to the auxin signaling network, which in turn activates metabolic and genetic programs that trigger cell elongation and cellulose synthesis to enable the plant to grow higher than the shadow-casting object (for an overview of how the synthesis of natural materials is controlled at the examples of plant cell walls and bones, see Refs. [[8], [9], [10], [11]]).

In this review article, we argue that the key to making materials with properties and functions similar to those in nature is to gain precise control over the sensing, processing, and actuation machinery of living cells (Fig. 1b). The capabilities for such control have been growing exponentially in the past two decades with the emergence of synthetic biology. Synthetic biology uses the classical engineering cycle of design, build, test, and learn [12,13]. In the first step, the biological system is designed based on modular biological building blocks such as receptors, genes, and enzymes. To refine the design, mathematical models simulating the interplay of the different parts have proven highly valuable [14]. Subsequently, the system is built and tested for performance. The outcome of such testing provides information on how to further tune the system in the next iterations toward reaching the desired specifications. Following this approach, synthetic biology is increasingly providing access to the cell's sensing, processing, and actuation machinery. For example, different input sensors responsive to physical (multichromatic light, temperature), chemical (drugs, metabolites), or electrical (applied voltage or current) stimuli can be read out and functionally linked to desired downstream processes. Subsequently, such input information can be processed by synthetic networks functioning as amplifier, integrator, oscillator, time-delay, or memory. Further modes of synthetic signal processing include Boolean algebra operations, robust and perfect adaptation as well as non-linear feedback and feed-forward loops (reviewed elsewhere [15]). Also, the installation of (optogenetic) switches at key points of cell signaling yields external control of signal processing [16]. Finally, the outcome of such signal processing can be wired to desired cellular actuation. This could be, for example, the up- and downregulation of endogenous or transgenic biosynthesis and biodegradation pathways [17,18], the growth, migration, differentiation, or death of a cell, or the transmission of information to other cells for steering collective cell behavior. Such engineering of cellular signal sensing, processing, and actuation has already now a huge impact on the biomedical, energy, agriculture, fine- and bulk-chemical as well as environmental fields (for further reading, see Refs. [[19], [20], [21], [22], [23]]). Fueled by fundamental research and motivated by economically attractive markets [24], synthetic biology has matured to a stage where it can now transform the way materials are designed, synthesized, used, and disposed [[25], [26], [27], [28], [29], [30], [31]].

**Fig. 2:**
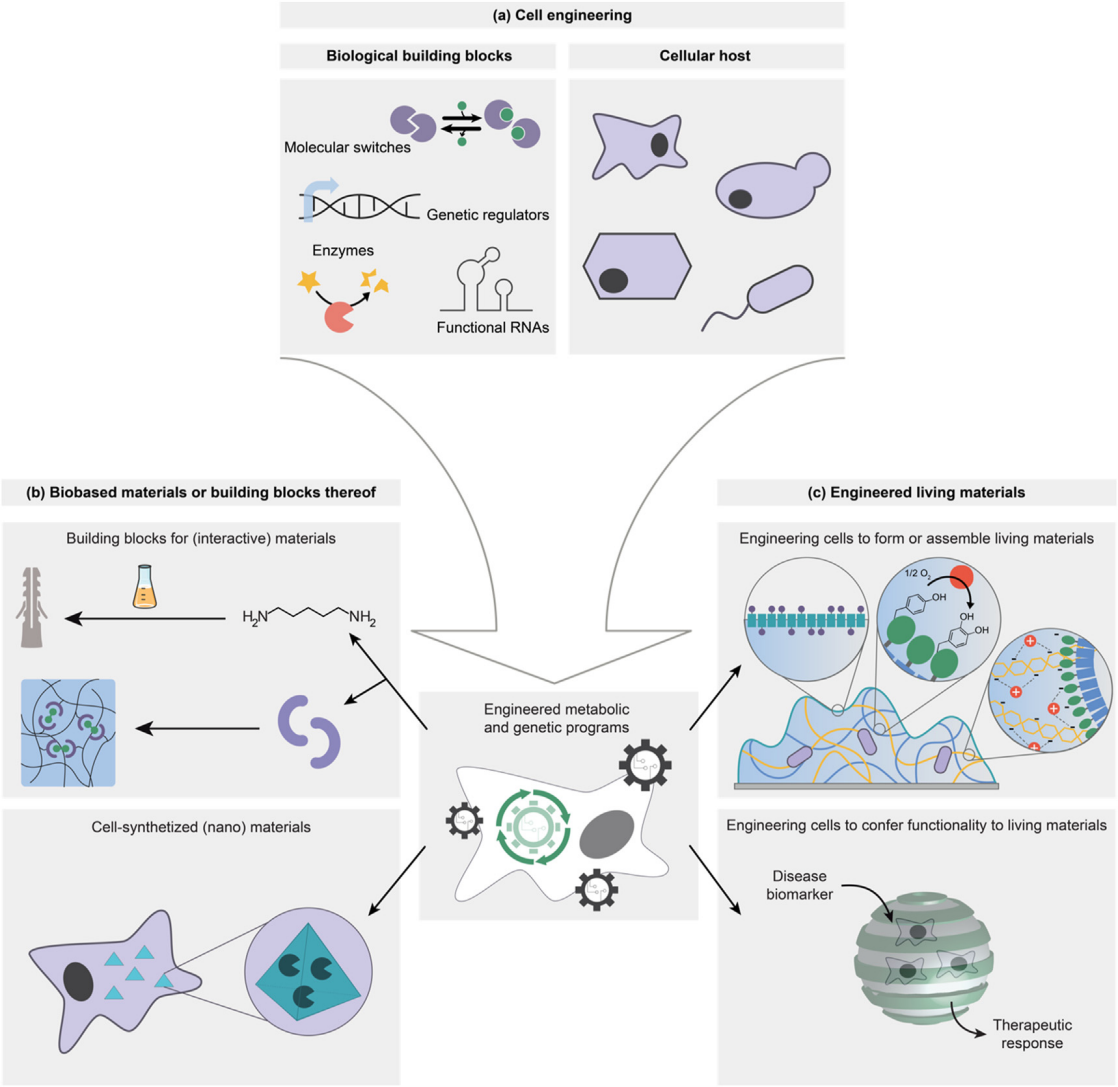
**Opportunities of synthetic biology for driving the biologization of materials sciences. a.** Cell engineering. Modular biological building blocks are assembled to switches and networks to program cell fate and function. **b.** Cells are engineered to produce building blocks for the subsequent chemical synthesis of materials (see Section 2 of this article). These can either be, for example, monomers for the synthesis of polymer materials, or biological macromolecules such as proteins as functional building blocks for the synthesis of (interactive) materials. Furthermore, cells can be engineered to synthesize nanostructured materials. Parts of the image were produced using an image modified from Servier Medical Art (Servier, www.servier.com, licensed under a Creative Commons Attribution 3.0 Unported Licence, CC BY 3.0). **c.** Engineered living materials (see Section 3 of this article). Cells can be engineered to form or assemble living materials of which they remain an integral part. These can be, for example, construction materials or adhesives. Complementarily, cells can be engineered to confer specific functional performance to living materials to operate, for example, as living electronic devices or as self-regulated, self-replenishable drug depot.

In this review article, we identify and review two areas where synthetic biology is driving the biologization of materials sciences (Fig. 2). First, we review how cells can be engineered to synthesize precursors and building blocks for the subsequent synthesis of structural and functional materials (Section 2). This includes already existing materials, the precursors of which can now be synthesized from renewable resources but also materials with properties and functions that have so far been out of reach without the tools and technologies provided by synthetic biology. Second, we review the emerging field of engineered living materials (ELMs), where living cells are an integral part of the material (Section 3). Here, cells either contribute to the formation or assembly of the material or provide novel functionality to the living composite while remaining an integral part of it. We conclude this review by discussing future challenges and opportunities for the further development of synthetic biology-based materials (Section 4).

## 2 Engineering cells to synthesize (precursors of) non-living materials

Advances in understanding natural biosynthesis pathways and the strongly increasing capability to engineer cell function at multiple levels have yielded novel opportunities for the production of biomolecules with desired structure and function. Such bio-produced compounds can either be used to replace materials synthesis routes relying on non-regenerative resources such as petrochemicals, or they can be used to confer novel functionality to materials hitherto out of reach for classical chemistry-based materials synthesis. In this section, we review strategies for the bio-based production of materials synthesized from small-molecule metabolites (Section 2.1), polysaccharides (Section 2.2), nucleic acids (Section 2.3), inorganic materials (Section 2.4), as well as engineered proteins (Section 2.5).

### 2.1 Metabolic engineering to produce precursors for materials synthesis

The synthesis of polymer materials is commonly based on the polymerization of precursors derived from petrochemical processes. In light of the increasing environmental challenges we are facing, the development and strengthening of synthesis routes from renewable resources have become a main interest in today's materials research. To this aim, metabolic engineers have endowed microorganisms with the ability of producing polymer precursors by reprogramming their metabolic fluxes.

**Fig. 3:**
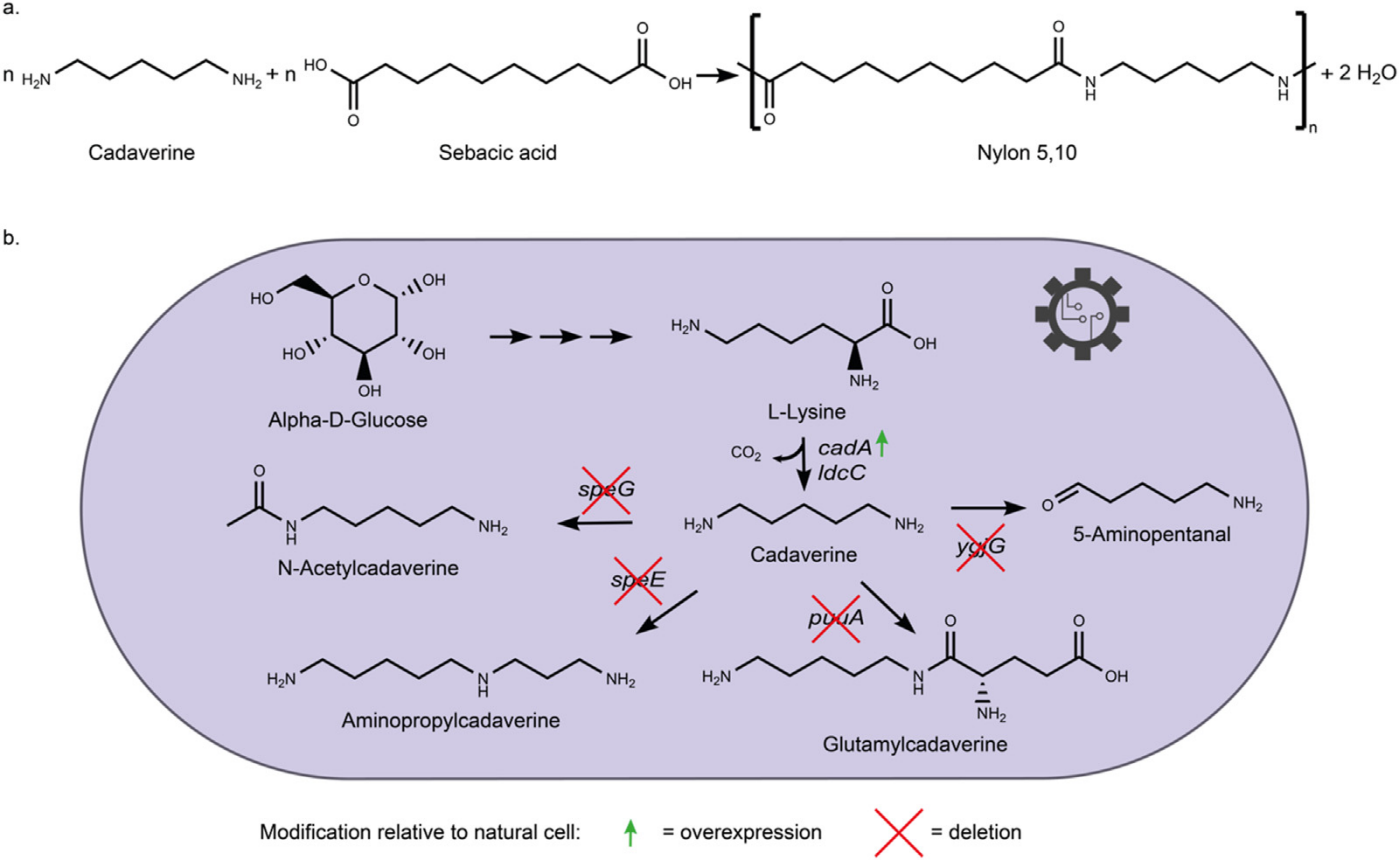
**Bio-based synthesis of nylon. a.** Nylon can be synthesized by polymerization of a linear diamine (here, cadaverine) with a linear dicarboxylic acid (here, sebacic acid). **b.** Bio-based production of cadaverine. Cadaverine was synthesized by decarboxylation of lysine. To increase yield, the decarboxylase gene *cadA* was overexpressed while genes encoding enzymes that metabolize cadaverine were deleted [32].

Nylon is part of a wide range of commodity goods [32] and is used in many different pharmaceutical and industrial applications. For example, in the medical sector, nylon is favored because of its good biocompatibility. Fields of application include, for example, scaffolds for tissue cultures [33]. About 9.7 million tons of nylon were globally produced in 2020 [32]. Nylon precursors are derived from a variety of sources, most of which are petrochemicals. Owing to the need for more sustainable synthesis routes, there is an increasing interest in the bio-based production of nylon precursors. Although some nylons, such as nylon-6, are produced through ring-opening polymerization synthesis from lactam monomers, other nylons, such as nylon-66, are produced through polycondensation of diamines and dicarboxylic acids [32,34]. One of the latter, adipic acid, the most abundant monomer in the production of nylon-66, has been produced by metabolic engineering of *Escherichia coli* and *Saccharomyces cerevisiae* [35,36]. In *E. coli,* a five-step reverse adipate-degradation pathway has been engineered. Moreover, cadaverine (1,5-diaminopentane, also referred to as pentane-1,5-diamine or PMD), a diamine precursor for polyamides, is another potential candidate for the production of bio-based nylon [32,33]. The Lee group produced cadaverine in metabolically engineered strains of *Corynebacterium glutamicum* and *E. coli* using glucose as a substrate [34]. In *E. coli,* cadaverine is produced by decarboxylation of l-lysine by two l-lysine decarboxylase isozymes, which are encoded by the *cadA* and *ldcC* genes. For enhanced cadaverine production, *cadA* has been overexpressed and genes encoding for enzymes that divert cadaverine or its precursors to other pathways have been deleted (Fig. 3) [32]. More recently, cadaverine has been produced by displaying beta-xylosidase BSU17580 derived from *B. subtilis* on the cell surface of *C. glutamicum*, providing a solution for the cadaverine production from cheap xylooligosaccharides as a carbon source [37]. Besides the bio-based production of diamines and dicarboxylic acids, other nylon precursors such as lactams and their ω-amino acid precursors have also been produced by metabolically engineered strains [34].

For example, polylactic acid is a widely used biodegradable bioplastic, composed of poly-l and poly-d-lactic acid. Recently, lactic acid has been produced in metabolically engineered *Bacillus subtilis* and *C. glutamicum*, among others [38,39]. The Inui group used metabolic engineering of *C. glutamicum* to increase l-lactic acid production from 73 g/L to 212 g/L with a yield of 97.9% and d-lactic acid production from 112 g/L to 264 g/L with a yield of 95.0% [39]. Unlike natural lactic acid producing strains, *C. glutamicum* does not need a nutrient-rich medium but only requires cost-effective mineral salts to produce l-lactic acid from carbon sources. Consequently, metabolic engineering of this strain could simultaneously increase production yield and reduce costs [39]. Table 1 provides further references to reviews and original research articles describing how metabolic engineering can be applied to produce precursors for polymer materials.

**Table 1:** Overview of metabolic engineering approaches to produce monomers for polymer synthesis.

Substance	Description	Reference
Different monomers	Review of bio-based production of monomers and polymers by metabolically engineered microorganisms	[40]
Diamines, dicarboxylic acids, and ω-amino acids	Review of bio-based production of monomers and polymers by metabolically engineered microorganisms	[41]
Dicarboxylic acids and diamines	Review of metabolic engineering to produce dicarboxylic acids and diamines	[32]
Lactams and ω-amino acids	Engineered strains for lactam production as a precursor for nylon synthesis	[35]
Cadaverine	High-efficiency and low-cost production of cadaverine using engineered *E. coli*	[42]
Lactic acid	Review of microbial production of lactic acid	[43]
l-lactic acid	l-lactic acid–producing strain developed through CRISPR-Cas9 gene-editing platform	[44]
Adipic acid	Metabolic engineering of *E. coli* for producing adipic acid through the reverse adipate-degradation pathway	[36]
Polyhydroxy-alkanoates	Co-production of microbial polyhydroxyalkanoates, used as biodegradable plastics, with other chemicals of industrial interest	[45]
Polyhydroxy-butyrate	Microbial engineering for the production of polyhydroxybutyrate	[46]
Styrene	*E. coli* was engineered for styrene production by targeting 54 genes with 85,,420 mutations. The yield was improved 3.45-fold. Produced styrene was subsequently polymerized to polystyrene by free radical synthesis.	[47]
Di-amines	Microbial engineering to produce di-amines for the synthesis of hyaline for use in the electronics industry	[48]
Glutaric acid	Metabolic engineering of an l-lysine–overproducing *C. glutamicum* strain	[49]

### 2.2 Engineering cells to produce polysaccharide-based materials

Biopolymers formed from polysaccharides have properties of interest for industrial and medical applications. For example, polysaccharides are used in drug delivery, wound healing, as adhesives, or as food stabilizers and additives. Polysaccharides are naturally produced by cells for the modification of proteins and other biomolecules or as a structural component in the extracellular matrix. For example, polysaccharide-based bacterial cellulose is produced for the formation of a protective envelope around the cell. Also here, synthetic biology offers methods to confer desired properties to polysaccharides produced at a large scale [13,50].

Bacterial cellulose is of interest because of its crystalline structure and its high physical strength. Further advantages are its high water-holding capacity and its biodegradability mimicking the properties of natural tissues. These features make bacterial cellulose interesting for biomedical applications such as scaffolds for tissue engineering or artificial blood vessels [51]. To enhance or adapt the properties of bacterial cellulose, naturally produced by several *Acetobacteraceae* species, genetic engineering has been applied. One example is the production of bacterial cellulose in *Komagataeibacter* strains, which are highly acid-resistant and naturally produce bacterial cellulose in large quantities. In addition to the introduction or deletion of genes to enhance the production of bacterial cellulose, several genes have been introduced into bacterial cellulose-producing species to provide new metabolic pathways [52,53]. For instance, the introduction of an operon of three genes from the yeast *Candida albicans* in *Komagataeibacter xylinus* has enabled the production of a cellulose-chitin co-polymer, which can be degraded by animal lysozymes, unlike cellulose. Its ability to be degraded within the body makes it a useful material for the production of stents and vein prostheses, which would not require surgical removal [53]. The Ellis group pioneered a complementary approach to synthesize and engineer bacterial cellulose [54]. They isolated a strain of *Komagataeibacter rhaeticus*, a natural producer of bacterial cellulose. Based on the sequencing of the bacterium, they developed genetic tools to produce heterologous proteins and to control the formation of bacterial cellulose. These and other applications of bacterial cellulose are covered in a recent review by the same group [53].

Moreover, it is possible to build complex products with microbially produced materials. An example is the headset named Korvaa, built exclusively from microbially grown materials [[55], [56]].

In an interesting approach, the Sang Yup Lee group together with colleagues from Samsung evaluated the possibility of using bacterial cellulose as a separator for lithium-rechargeable batteries [57]. They metabolically engineered a *K. xylinus* strain for increased bacterial cellulose production, performed a fermentation at 30 L scale and further fibrillated the cellulose for disentanglement and homogenization. Subsequently, they processed the material to form a thin membrane, which was finally used to produce a cylindrical battery via a continuous roll-to-roll process [57].

In another example, the production of more complex sulfated glycosaminoglycans was engineered into *E. coli*. To this aim, the Koffas group introduced three metabolic pathways into cells to produce the three required components of chondroitin sulfate synthesis — chondroitin, a sulfate donor, and a sulfotransferase [58]. This study provides a simple synthesis route as an alternative to the conventional isolation of chondroitin sulfate from animal tissues. Other polysaccharide-based materials are alginate, chitin, chitosan, or hyaluronan. The production and properties of these materials can be engineered as well using synthetic biology strategies as recently reviewed elsewhere [[59], [60], [61]].

### 2.3 Engineering cells to produce nucleic acid–based materials

The design of DNA-based nanostructures assembled from synthetic DNA sequences via specific base-pairing represents a very active field of research opening many different applications in biomedicine, bio-imaging, and diagnostics, such as the development of nanosized optical sensors for pathogens [[62], [63], [64]]. Most of such DNA-based nanostructures are assembled from DNA synthesized *in vitro,* for example, by means of classical solid-phase chemistry yielding different applications as reviewed elsewhere [65,66]. Here, we describe synthetic biology approaches to genetically encode defined nucleic acid structures and to synthesize and assemble them in living cells. The intracellular synthesis of DNA structures allows, for example, the creation of artificial organelles but also significantly facilitates the upscaling of DNA nanostructure synthesis by simply cultivating the organisms at a larger scale. DNA nanostructures are commonly assembled from single-stranded DNA (ssDNA), a species not abundantly occurring in living cells. The Voigt group overcame this limitation by engineering *E. coli* to contain double-stranded DNA that was transcribed to non-coding RNA (ncRNA) further containing an HIV terminator-binding site (HTBS) [67]. The HTBS recruited the HIV reverse transcriptase, which then synthesized the desired ssDNA (32–205 nt). Intracellular assembly was demonstrated by the production of ssDNAs spontaneously assembling to four-part crossover nanostructures. In addition, the assembly of purified ssDNAs to one-dimensional DNA wires and two-dimensional DNA sheets has been shown [67].

In a complementary approach, the Dietz group engineered a cell-based approach for the mass production of DNA origami [68]. To this aim, they used bacteriophages producing single-stranded DNA that contained self-excising sequences encoding Zn^2+^-dependent DNA-cleaving DNAzymes. Following isolation of the single-stranded DNA, the addition of Zn^2+^ activated the DNAzymes and yielded the desired, short ssDNA molecules. With this approach, the authors obtained 163 mg folded DNA-based nanorods from a 1.9 L bioreactor culture [68]. This approach suggests that DNA molecules of arbitrary length and sequence can be produced at a large scale.

More recently, the Wang and Mao groups reported programmed self-assembly of RNA in bacterial cells [69]. For that, nanostructures composed of RNA-duplexes were formed from one long single-stranded RNA (ssRNA) in *E. coli*. This technique provided a cost-effective way for the production of nucleic acid nanostructures in large-scale quantities with prospective application in biomedicine, for example, by the introduction of functional RNA nanostructures controlling cellular processes *in vivo* [69].

### 2.4 Engineering cells to produce inorganic materials

Throughout evolution, several organisms have accomplished hierarchical self-assembly of mineral-based materials. The process by which organisms produce such materials is known as biomineralization [70,71]. The understanding of the fundamental mechanisms that drive those fabrications could help material engineers in their quest for biohybrid materials. Examples of research papers pertaining to mineral-based building blocks for biohybrid materials are summarized in Table 2. Materials built by harnessing the biomineralization process, including those using synthetic biology approaches have been extensively reviewed elsewhere [30,[70], [71], [72]]. Biomineralization is also a promising area in ELMs as described in chapter 3 of this article.

**Table 2:** Overview of biologically engineered inorganic materials.

Protein class	Description	Reference
Silicatein	A bioorganic–inorganic hybrid material is produced by the incorporation of nanoparticles into sponge spicules	[73]
Bacterial biofilm protein (CsgA), silaffin	A diatom frustule-like structure is built by harnessing a curli biofilm protein, a chitin-binding domain, and a silaffin peptide, which together serve as a scaffold for silica mineralization. The construction of an artificial photosynthesis system is achieved upon colonization of the material with living cells	[74]
Silaffin	Recombinant silaffin peptides from diatoms, which nucleate the formation of silica nanostructures, are produced in *E. coli* with different post-translational modifications, obtaining nanostructures with different properties	[75]
	A genetically engineered diatom produces silaffin peptides fused to proteins of interest, resulting in the immobilization of the protein in the diatom silica	[75]
	A genetically engineered diatom produces a silica matrix displaying antibodies on its surface. This material is applied for the targeted delivery of chemotherapeutic drugs	[76]
	A genetically engineered diatom produces a silica matrix that incorporates proteins of interest. The proteins' stability and functionality in the silica matrix is assessed	[77]
	Calcium carbonate production and crystal formation is achieved by urease-expressing engineered *E. coli* cells	[78]
Silaffin and Major ampullate Spidroin 1 (MaSp1) protein from spider silk	Protein-silica composites able to form structures with different morphologies were obtained by combining silica precursors with a spider silk protein fused to a silaffin peptide	[79]
Silicatein	Synthetic sponge spicules are obtained by means of recombinant silicatein mixed with inorganic compounds	[80]
Thiosulfate reductase & arsenal reductase	Redox pathways from two strains of bacteria are transferred into a heterologous host to synthesize arsenic sulfide nanomaterials	[81]

### 2.5 Engineering cells to produce proteins as precursors for materials

Natural and engineered protein-based materials such as silk, keratin, elastin, or resilin offer a myriad of applications in diverse fields (e.g. see reviews in Refs. [[82], [83], [84], [85]]). We here focus on recent synthetic biological and protein engineering strategies producing materials with desired structural and functional properties. We first review different classes of protein-based materials. Second, we review recent work, where protein materials with specific properties and functions have been engineered. These include (i) protein-based engineered nanostructures, (ii) designed liquid protein-based materials, and (iii) interactive protein materials.

#### 2.5.1 Materials based on engineered silk

Silk-based materials functionalized by molecular engineering offer manifold applications in the textile sector, in cosmetics production, or in the medical sector as implant coating [86]. The extraordinary properties of natural silk, such as its high durability and elasticity, has raised interest in producing these materials in industrial quantities and standards. For that, a gene derived from the spider *Araneus diadematus* and encoding silk proteins was recombinantly produced in *E. coli*. Protein production was induced and the recombinant silk protein was purified afterward [87,88]. Furthermore, genetic engineering allowed the production of functionalized silk harboring single amino acid substitutions, functional peptides, and chimeric proteins endowing the recombinant silk with non-silk functions. Interesting examples are silk-elastin-like polymers (SELPs), which combine the mechanical strength of silk and the thermoresponsive properties of elastin [86]. For an overview of approaches to engineer silk proteins, see reviews and original work listed in Table 3.

**Table 3:** Synthetic biological strategies for engineering silk proteins.

Protein	Strength	Description	Reference
Recombinant Spidroins based on MaSp1 (major ampullate spidroin) single repeat unit (1-mer)	Tensile: 1.03 ± 0.11 GPa	Large size spidroins (556 kDa, 192-mer) by ligation of two 96-mer recombinant proteins that replicate natural spider silk	[89]
Natural *N. clavipes* dragline	Tensile: 0.8–1.2 GPa	Large size spidroins (>300 kDa)	
MaSp1		Recombinant biocompatible and biodegradable spider silk protein as implantable optical waveguides	[90]
MaSp2		*E. Coli* enhanced production of spider silk protein	[91]
MaSp1/MaSpa2 1:1 mix	Tensile: 100.2 MPa	Copolymer behavior mimetics of spider silk proteins	[92]
Natural *A. aurantia* dragline	Tensile: 1702 MPa		
MaSp1&2, ADF4 (*Araneus diadematus* Fibroin)		Biomaterials applications (cosmetics, regenerative medicine, textile …)	[86]
MaSp1, ADF4, FN-4RC (fibronectine 4RepCT protein)		Spider silk proteins for 2D and 3D cell culture, tissue engineering (bone, cartilage, heart-muscle regeneration …)	[93]
Diverse spidroins		Biomedical applications (drug delivery, regenerative medicine, implant coating …)	[87]
e.g. eADF4 foam	Compression: 0.94–3.24 kPa	Bone and cartilage tissue engineering	
e.g. rS1/9 (recombinant spidroin) foam	Tensile: 190 kPa	Bone and cartilage tissue engineering	
ADF4 (among others)		Examples of silk and functional motifs used for engineered silk biomaterials and current companies with R&D and/or silk-based products for biomedical applications	[94]
ADF4		Spider silk protein as delivery system for vaccine application	[95]
I16 (recombinant major ampullate spidroin 1)		Membraneless organelle mimetics for cell compartment functionalization in *E. coli*	[96]
TuSp2-RP (tubuliform silk gland fibroin repetitive domain)	Tensile: 128 MPa	Silk protein-based material for seawater clean-up	[97]
Silkworm silk fibroin		Physicochemical state of the art of silkworm-based biomaterials: from composition and properties of proteins to applications	[98]
Silk fibroin		3D printing with silk fibroin	[99]

#### 2.5.2 Materials based on engineered elastin and resilin proteins

Materials based on elastin-like recombinamers (ELRs) or elastin-like peptides (ELPs) are used in, for example, drug-containing ELR-conjugates for intracellular drug release or ELR-based crosslinked hydrogels that enable the long-term delivery of a loaded therapeutic agent after implantation [[100], [101], [102]]. Often, material precursors are further engineered to equip the resulting materials with defined properties or to introduce novel functions. For instance, a precisely defined elastin-based rubber-like hydrogel for usage in tissue engineering has been designed by recombinant expression of designed ELPs. For that, the length of the sequence, as well as the components of the ELPs have been adjusted before cross-linking of the peptides with 4-arm N-hydroxysuccinimide-functionalized polyethylene glycol (PEG/NHS) [103]. Another material building block suitable for processing hydrogels is resilin-like proteins (RLPs), a class of elastomeric proteins found in insects. In a recent study, RLPs were crosslinked to hydrogels [104]. The further incorporation of glycerol yielded adhesive properties. Finally, the hydrogel was doped with graphene yielding a stretchable (over 4 times of its original length), adhesive (24 kPa), and electrically conductive material. The hydrogel was used as an electrical sensor, for example, for measuring the bending of a finger. Such materials are promising for the development of smart wearables [104]. For further information on strategies to engineer elastin- and resilin-based materials, see reviews and original work in Table 4.

**Table 4:** Engineered elastin- and resilin-based materials.

Protein class	Description	Reference
Elastin-like recombinamers (ELRs)	Drug, vaccine, and gene delivery systems based on ELRs in biomedical applications and the use of ELR-based hydrogels in tissue engineering and regenerative medicine	[105]
Elastin-like peptides (ELPs)	Elastin-like polypeptides in drug delivery	[101]
ELPs, silk-elastin-like peptides	ELP constructions for drug delivery system. Methods for fabrication and biomedical applications.	[106]
ELPs	Design and representative biomedical applications of ELPs, focus on tissue engineering and drug delivery	[100]
Elastin	Elastin-related biomaterials and their benefit on wound healing	[107]
ELRs	Elastin-like hydrogels for tissue engineering applications, general use in different biomedical fields	[108]
Elastin, silk, collagen, resilin	Recombinant biomaterials and applications	[109]
Elastin, collagen, silk, resilin & -like proteins	Proteins in nanomaterials, modular design, applications in tissue engineering and drug delivery	[110]
Tropoelastin & resilin	Tropoelastin & resilin-based biomaterials, applications in tissue engineering, (composite materials with silk)	[111]
RLPs	Liquid–liquid phase separation to generate microstructured hydrogels (applications in tissue engineering and drug delivery)	[112]
RLPs	Genetic control of material properties through modular design of RLPs and via chemical crosslinking (responsiveness to multiple stimuli, mechanical properties, cell adhesion, and proliferation)	[113]
RLPs	Engineering mechanical properties, self-assembly, and phase separation, autofluorescence (applications as multifunctional materials in tissue engineering or nanomaterials)	[114]
β-hairpin, α-helical coiled coil peptides, ELPs, silk fibroin &resilin	Hydrogels with (natural/engineered) peptides or proteins in biomedicine	[115]
RLPs	Elastomeric and cell-adhesive material based on RLPs (potential for growth factor delivery and proteolytic remodeling)	[116]
RLPs	Hydrogels for tissue engineering	[117]

#### 2.5.3 Materials based on engineered reflectins

Reflectins form a protein family that allows cephalopods to manipulate light [[118], [119], [120]]. They are capable of self-assembly and form nanostructures that show thin-film interference and infrared camouflage. These properties have been leveraged to develop protein-based optical materials such as thin films and coatings on different materials (e.g. glass, silicon, graphene oxide). Recombinant reflectin-like peptides have been designed based on conserved repeated motifs present in natural reflectin isoforms to improve solubility, expression, and purification. The refractive indices of these films, and thus their optical appearance, have been shown to be tunable by chemical, mechanical, and electrical stimuli making them suitable for adjustable camouflage systems and biophotonic technologies [[118], [119], [120]]. Reflectins and other optically active recombinant proteins are reviewed in more detail elsewhere (Table 5).

**Table 5:** Optically active recombinant proteins.

Protein class	Description	Reference
Reflectins	Tunability of reflectins and their potential for optoelectronics	[120]
Reflectins	Reflectins as materials for color-changing coatings	[119]
Reflectins	Mainly about natural optical mechanisms, recombinant reflectins briefly mentioned	[118]
Reflectins and others	Proteins in optical and electronic materials	[121]
Different peptides	Peptide nanophotonics (caused by a certain structure/associated with dye), applications in medicine, biochips	[122]

#### 2.5.4 Materials based on engineered mussel foot proteins

Another interesting class of protein materials is mussel foot proteins. Mussel foot proteins form glue-like sticky materials with excellent underwater adhesive properties. The proteins are based on proteins contained in the byssus of mussels and have been subjected to extensive engineering to confer adhesive functions to protein materials. This topic has been recently covered elsewhere. See Table 6 for references to review articles and selected original work.

**Table 6:** Synthetic biology-inspired materials based on mussel foot proteins.

Proteins	Strength/Energy	Description	Reference
rMAP fp-151 (recombinant mussel adhesive protein, foot protein-151)		Bulk adhesive strength comparison of recombinant hybrid mussel adhesive protein with and without the addition of chemical cross-linkers reagents	[123]
e.g. modified rMAP fp-151	Adhesion strength: 0.42–1.06 MPa		
e.g. unmodified rMAP fp-151	Adhesion strength: 0.33 MPa		
e.g. white glue	Adhesion strength: 0.17 ± 0.04 MPa		
MAP fp-151		Antibacterial coating using MAP	[124]
rMAP, e.g. rfp-151-RGD (recombinant foot protein-151-arginylglycylaspartic acid)	Underwater porcine skin adhesion: rgd:120 kPa Underwater rat bladder tissue adhesion: rgd:140 kPa Underwater aluminum adhesion: rgd:880 kPa	Mfp: summary of diverse applications (coating materials and bioadhesives)	[125]
Mfp (mussel foot protein), TMPs (thread matrix proteins), preCol (collagenous thread proteins)		Summary of recombinantly produced mussel byssus proteins: from protein description to production strategy. Mfp are therefore described and used as solutions, nanofibrils, coacervates, and hydrogels	[126]
Mfp3, Mfp 5, and CsgA^a^	Underwater adhesion energy: 20.9 mJ m^−2^	Hybrid molecular material: protein fusion between Mfp^a^ and *E. Coli* curli fibers for an enhanced adhesion	[127]
Pvfp-5β (*Perna viridis* foot protein 5β)		Recombinant mussel protein with tissue bioadhesive potential	[128]

a CsgA: *E. coli* major curlin subunit.

#### 2.5.5 Materials based on engineered curli

Many bacterial species naturally form a biofilm by producing a protective extracellular matrix composed of saccharides and proteins and, to a smaller extent, nucleic acids. Recent research has focused on curli, a protein-based amyloid nanofiber that represents the main proteinaceous structural component of biofilms formed by *E. coli* [129]. Curli forms a tangled, robust network that encapsulates the producing bacteria. Curli is synthesized from the secreted protein CsgA with the help of the outer-membrane protein CsgB, which mediates CsgA nucleation and anchoring of the resulting nanofibers to the cell surface. For curli synthesis, further genes located in the two curli operons are required for processing (CsgE, CsgF) and secretion (CsgC, CsgG) [129]. Curli proteins can be genetically engineered, produced by *E. coli*, and subsequently purified and further processed for different applications. An overview of recent work on producing and processing curli protein is shown in Table 7. Curli-based living materials are reviewed in Section 3.1.2.

**Table 7:** Materials based on engineered curli.

Development	Description	Reference
Aquaplastic	Protein-based hydrogels from engineered curli biofilms.	[130]
Truncated CsgA-like self-assembling scaffold	Minimum required structure to drive the assembly of curli fibers and to aggregate into supramolecular materials and hydrogels. Applied to produce hydroxyapatite (HAP)-containing curli-like amyloid fibers	[131]
Amyloid curli fibers–alginate nanocomposite	Amyloid supplementation to alginate hydrogels for enhanced stiffness.	[132]
Electron transfer curli nanofibers	Engineered *E. coli* system for the synthesis of conductive protein networks.	[133]

#### 2.5.6 Protein-based engineered nanostructures

This section describes advances in the design and production of protein nanostructures such as nanocontainers and nanovesicles, nanosized hydrogels, as well as *de-novo* designed protein-based platonic solids (protein origami). Interesting starting points for the synthesis of these nanomaterials are naturally occurring protein cages [134], such as lumazine synthase assemblies that naturally form icosahedral capsids [135]. Based on the lumazine synthase (AaLS) from *Aquifex aeolicus,* the Hilvert group developed a tight protein container by directed evolution [136]. To this aim, acidic amino acids were introduced into AaLS leading to a negative charge on its surface (AaLS-neg). Furthermore, a positively charged deca-arginine sequence (R_10_) was attached to the C-terminus of the HIV protease leading to its sequestration in the AaLS-neg capsid. This sequestration partially neutralized the toxic effect of the protease on the production of host *E. coli*. In the next step, a plasmid-based library encoding mutated versions of AaLS-neg was created and introduced into *E. coli* also co-expressing the gene for the positively charged HIV protease. In this configuration, AaLS-neg variants that efficiently shielded the HIV protease from the cytoplasm provided a growth advantage for the respective host cells that could subsequently be selected for. It was shown that the evolved protein containers had an increased loading capacity for HIV protease [136]. In a similar approach, the Baker group evolved a protein nanocontainer to encapsulate its own RNA genome [137]. Such protein containers may have applications in drug delivery, in the formulation and stabilization of enzymes, or in bioremediation.

**Fig. 4:**
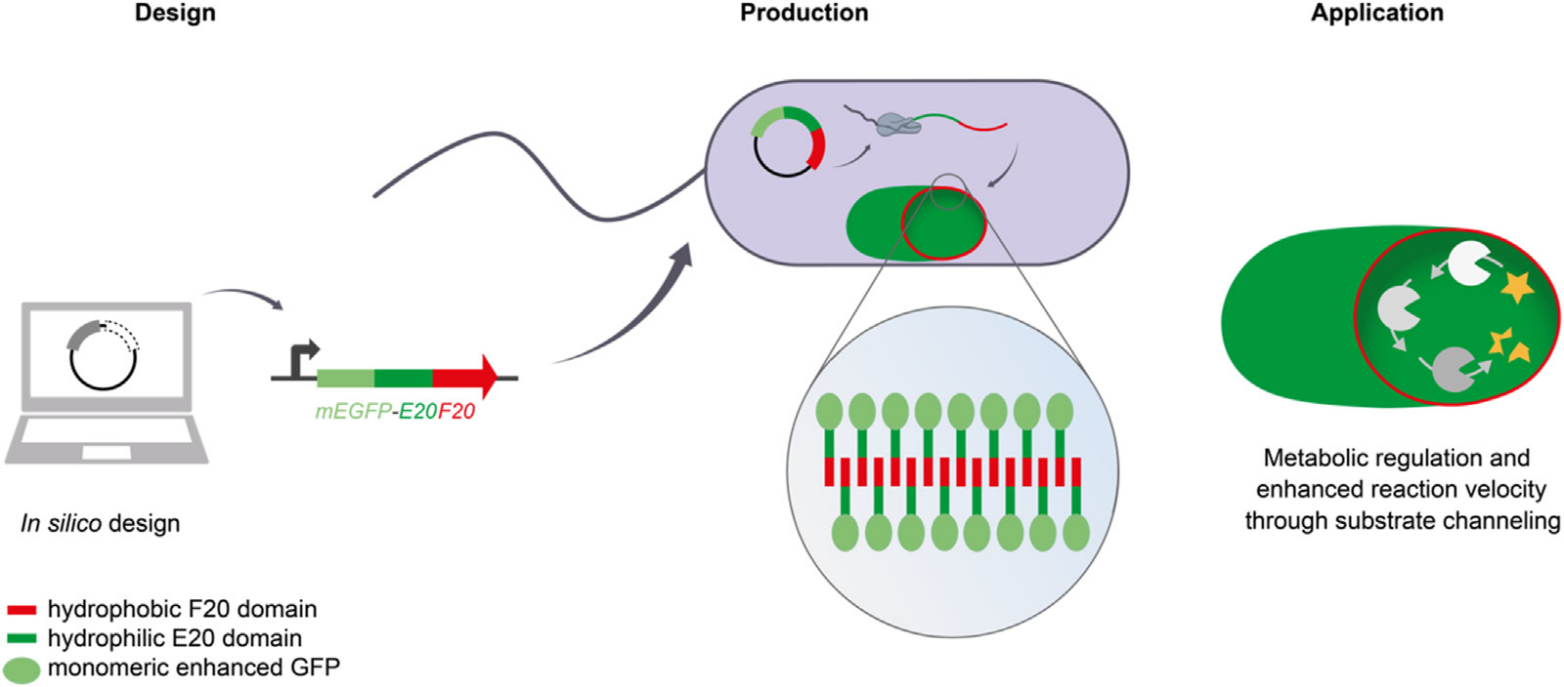
**Synthetic intracellular organelles formed by an amphiphilic designer protein.** A protein sequence encoding amphiphilic domains derived from repetitive elastin-like peptides fused to green fluorescent protein was designed and produced in *E. coli*. After protein production, the amphiphilic proteins spontaneously assembled into an intracellular organelle. This could, for example, be used to accelerate metabolic fluxes within such organelles through substrate channeling [138].

Another example for synthetic biology-based proteinaceous nanomaterials is self-organizing vesicles assembled from amphiphilic proteins. The Schiller group designed and produced such amphiphilic proteins, based on repetitive motifs from elastin-like peptides, in *E. coli* [138]. By fusing a fluorescent protein to them, the formation of intracellular vesicles could be observed. The vesicles were suggested to serve as synthetic reaction compartments for enzymes catalyzing desired bioconversions (Fig. 4) [138].

More recently, computational approaches for the *de novo* design of protein-based nanomaterials have attracted great interest [139,140]. The Jerala group used such an approach for the design of polyhedral protein cages based on coiled coil protein dimers, giving rise to tetrahedrons, square pyramids, or triangular prisms *in vitro* but also in bacterial and mammalian cells [140,141]. Protein-based coiled coil dimers can be orientated either in parallel or antiparallel, which allows the formation of more complex structural designs than with DNA-strands that can only be hybridized in antiparallel orientation. Such coiled-coil-based nanomaterial structures may be used as drug carriers in molecular therapies. For example, the presentation of antigens on the surface of protein nanomaterials is suggested to yield vaccines inducing a stronger immune response than using the non-immobilized antigens [139,142].

The incorporation of enzymes into protein nanostructures formed via coiled coil domains was shown to accelerate multistep catalytic reactions through a process known as substrate channeling [142]. Following this approach, the biosynthesis of resveratrol and mevalonate has been optimized in *E. coli* and *S. cerevisiae*, respectively. For example, connecting the resveratrol biosynthesis enzymes 4CL (4-coumarate coenzyme A ligase) and STS (stilbene synthase) via coiled coil domains yielded a 1.3-fold increase in resveratrol biosynthesis compared to the non-clustered enzymes. Similarly, in *S. cerevisiae*, the production of mevalonate could be improved 8.8-fold by forming a nanomaterial comprising the mevalonate biosynthesis enzymes ERG10 (Acetyl-CoA acetyltransferase), HMGS (3-Hydroxy-3-methylglutaryl-coenzyme A synthase), and tHMGR (truncated HMG-CoA reductase) via a coiled coil scaffold [143].

The above-described examples demonstrate the potential of producing genetically encoded proteinaceous nanostructures with defined topology. Further development of this field will strongly depend on the availability of reliable computational methods for designing proteins with desired secondary, tertiary, and quaternary structures. Unlike the computational design of complex DNA structures, predicting protein structures from the amino acid sequence remains highly challenging. A very promising approach in this direction is the application of deep learning algorithms trained on the large palette of existing 3D protein structures. For example, the program AlphaFold developed by the Google subsidiary DeepMind is based on deep learning and outperformed previous structure prediction algorithms (see below) [144].

#### 2.5.7 Designed liquid protein-based materials

**Fig. 5:**
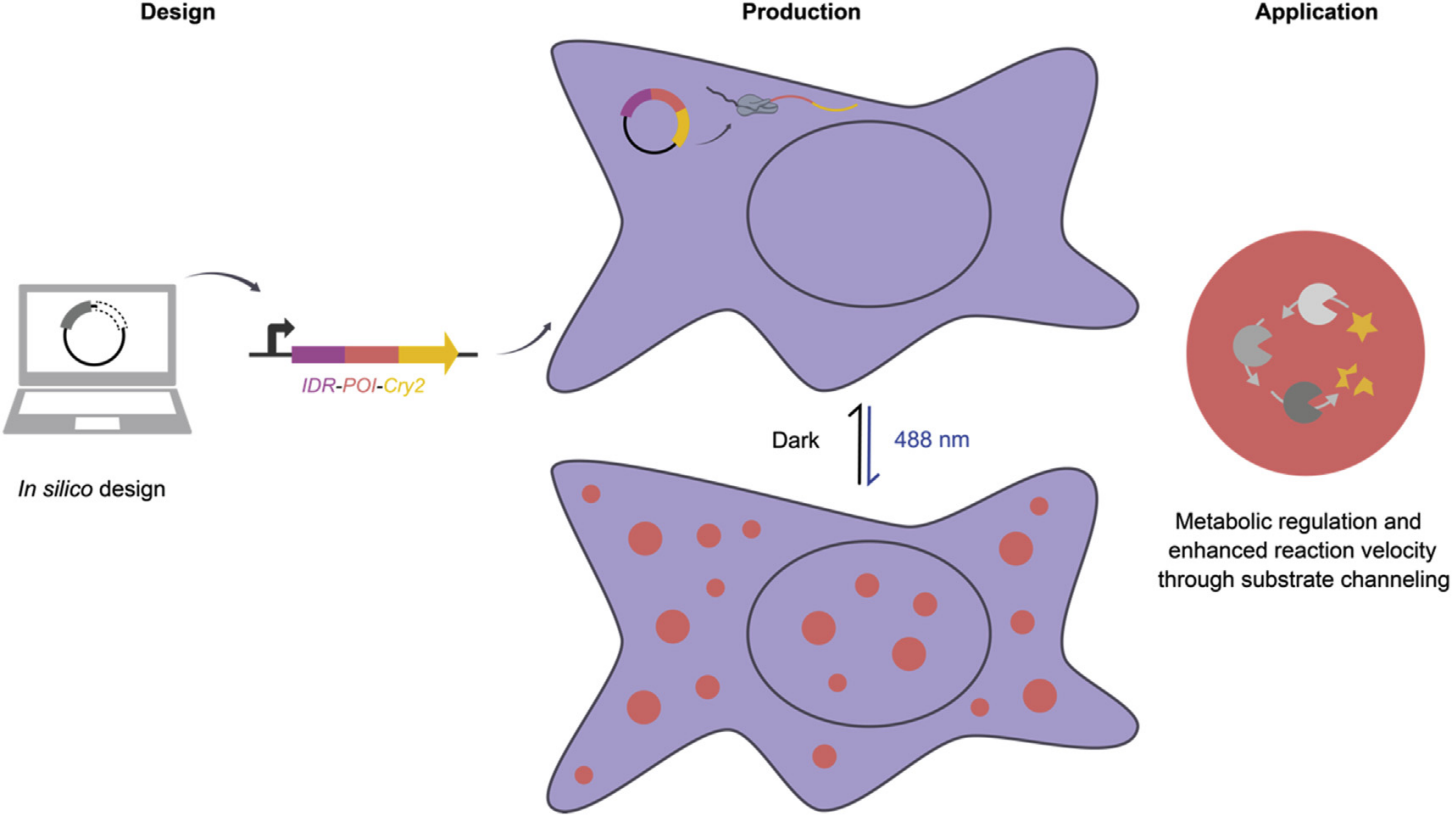
**Design, assembly, and application of liquid nanomaterials.** A protein comprising an IDR, a protein of interest (POI), and the blue light photoreceptor cryptochrome 2 (Cry2) was designed and produced in mammalian cells. Upon blue light (488 nm) illumination, Cry2 homo-oligomerized led to liquid–liquid phase separation mediated by the IDR domain. When using a fluorescent protein as POI, fluorescent liquid droplets were observed in the cell [146]. Using metabolic enzymes as POI, liquid materials could be formed with superior substrate processing capability due to substrate channeling [147].

The discovery that proteins undergo liquid–liquid phase separation in cells to form membrane-less organelles as distinct reaction compartments [145] yielded the opportunity to engineer liquid protein materials. Liquid–liquid phase separation of proteins can generally be induced by an increase in the concentration of proteins containing multivalent protein–protein interaction domains or specific sequences such as intrinsically disordered regions (IDRs). Several approaches have been developed to synthetically induce the formation of such liquid materials. For example, the Toettcher and Brangwynne groups developed an optogenetic platform for the spatiotemporal control of liquid–liquid phase separation [146]. For that, self-associating IDRs were fused to the *Arabidopsis thaliana* blue light receptor cryptochrome 2 (Cry2), which homo-oligomerizes upon illumination with blue light. It was shown that multimerization of Cry2-IDR fusions triggered liquid–liquid phase separation as observed by the formation of nano-sized ‘protein droplets’ within the cells (Fig. 5) [146]. This so-called ‘OptoDroplet’ system was used for the formation of metabolically active liquid nanomaterials. For example, the Toettcher group fused two enzymes of the deoxyviolacein biosynthesis pathway, VioE and VioC, to droplet-forming IDRs and the PixE/PixD blue light–responsive multimerization system [147]. They showed that bringing both enzymes into close proximity within the liquid nanomaterial resulted in a six-fold improved product formation rate while simultaneously increasing product specificity by 18-fold. Given the modular nature of the IDRs and the photoreceptors, this approach is extendable to recruiting other proteins into liquid nanomaterials. For example, a recent study showed that the formation of coacervates of synthetic transcription factors enhanced transcription activation in mammalian cells and mice [148].

As an alternative approach for the formation of protein-based liquid nanomaterials, the Good and Hammer groups developed an enzyme-triggered approach. In this study, repeats of the intrinsically disordered arginine/glycine-rich RGG domain from the P granule protein LAF-1 were fused to the maltose-binding protein (MBP) as solubility tag, with a tobacco etch virus (TEV) protease cleavage site in between. The addition of TEV protease cleaved off the MBP tag, resulting in phase separation of the RGG domains. The study further demonstrated that additional cargo, such as fluorescent proteins, could be recruited to the liquid nanomaterial by fusing them to an RGG domain [149].

#### 2.5.8 Interactive materials based on switchable proteins

**Fig. 6:**
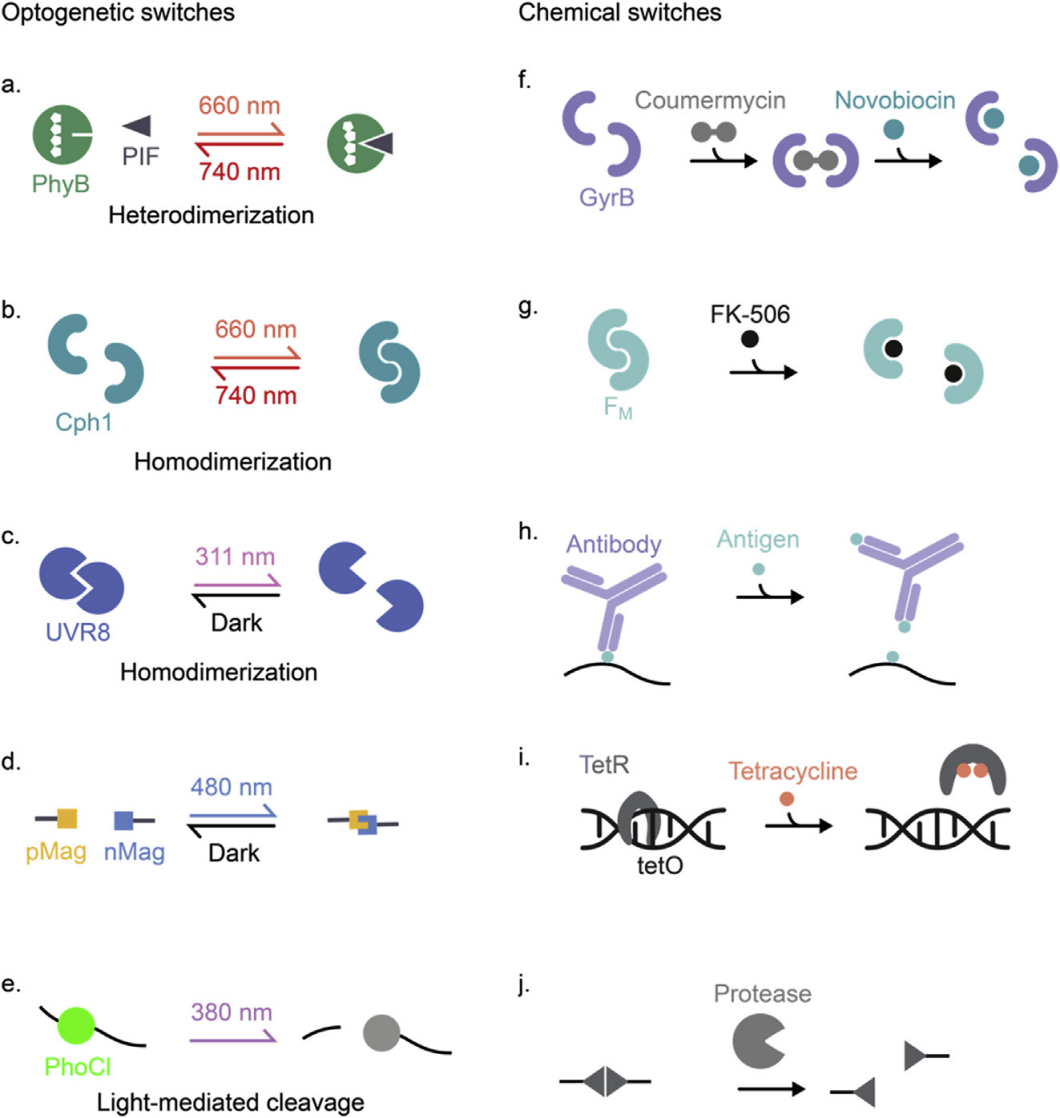
**Examples for genetically encoded switches used in synthetic biology and materials sciences.** a.–e. Optogenetic switches; f.–j. chemically responsive switches. **a.** Heterodimerization of PhyB and PIF under illumination with 660 nm and dissociation under 740 nm light [156]. **b.** Homodimerization of Cph1 under irradiation with 660 nm and dissociation under 740 nm light [157]. **c.** Dissociation of UVR8 homodimers under irradiation with 311 nm. Spontaneous reversion to dimeric state in the dark [158]. **d.** Heterodimerization of light, oxygen, and voltage-sensing (LOV) domains pMag and nMag after irradiation with 480 nm light and spontaneous reversion in the dark [159]. **e.** Light-mediated irreversible cleavage of PhoCl backbone after irradiation with 380 nm light [160]. **f.** Dimerization of GyrB by coumermycin and monomerization by the addition of novobiocin [161]. **g.** Spontaneous dimerization of F_M_ protein and monomerization in the presence of FK-506 [162]. **h.** Spontaneous binding of an antibody (fragment) to its antigen linked to a domain of interest such as a polymer. Competitive dissociation of the antibody by the addition of free antigen [163]. **i.** Spontaneous binding of the protein TetR to its operator DNA sequence tetO. Dissociation by the addition of tetracycline [164]. **j.** Cleavage of a specific protein sequence by precision proteases [156,165,166].

The emergence of synthetic biology was driven by the development of molecular switches enabling the external control of the fate and function of living cells [150]. These switches translate a physical or chemical input into a molecular response such as a conformational change in a protein structure, a change in protein–protein or protein–DNA interactions, or a change in enzyme activity. Beyond cell engineering, such switches have recently been applied for the synthesis of stimulus-sensitive hydrogels or of information-processing materials systems. Synthetic biology-based switches are derived from biological systems and thus are inherently functional and optimized for operation in a biological background. This is a notable advantage compared to classical chemical switches such as azobenzene-based photoswitches [151] that show undesired cross-reactivities with abundant biomolecules, for example, with serum albumin [152]. Key advantages of synthetic biological switches are as follows: (i) the sensitivity to input stimuli in the physiological concentration range, (ii) a high specificity toward their target and low/no unspecific cross-reactivities despite a complex environment, (iii) the full reversibility of the switching in some cases, (iv) the easy biotechnological synthesis from renewable resources, and (v) their complete biodegradability [153]. Fig. 6 shows a selection of synthetic biology-derived switches that have been used for the synthesis of stimulus-responsive and information-processing materials. A comprehensive overview of light-responsive molecular switches and their different applications is described in the database OptoBase [154,155].

##### 2.5.8.1 Stimuli-responsive materials based on engineered biological switches

The switches shown in Fig. 6 translate chemical or light stimuli into a change in the structure, interaction, or integrity of a biomolecule. Such switches have been applied to (reversibly) crosslink polymers, yielding hydrogels with switchable gel–sol transition or with switchable stiffness. For example, responsive hydrogels were synthesized by crosslinking polyacrylamide via dimers of gyrase B (GyrB) or F_M_ proteins or via the TetR/tetO and HucR/hucO repressor protein/DNA operator pairs. These systems allowed gel dissolution by the addition of the clinically licensed drugs novobiocin, FK506, and tetracycline, or by the gouty arthritis-related metabolite uric acid, respectively [161,162,164,167]. Similarly, eight-arm PEG was crosslinked to a hydrogel by the interaction of a humanized antibody fragment with its antigen fluorescein [168]. This hydrogel was applied as a remote-controlled drug depot for developing a single-injection-based vaccination protocol. In this configuration, the boost dose of a vaccine against the human papillomavirus was physically entrapped in the hydrogel and administered to mice together with the first (prime) dose. One week later, the clinically licensed fluorescein was given orally to induce dissolution of the implanted depot and release the second (boost) dose. The antibody titers obtained with this depot strategy compared favorably to a conventional vaccination regime involving two (prime and boost) injections [168,169]. Besides, no adverse reactions toward the depot were observed by histological analysis.

**Fig. 7:**
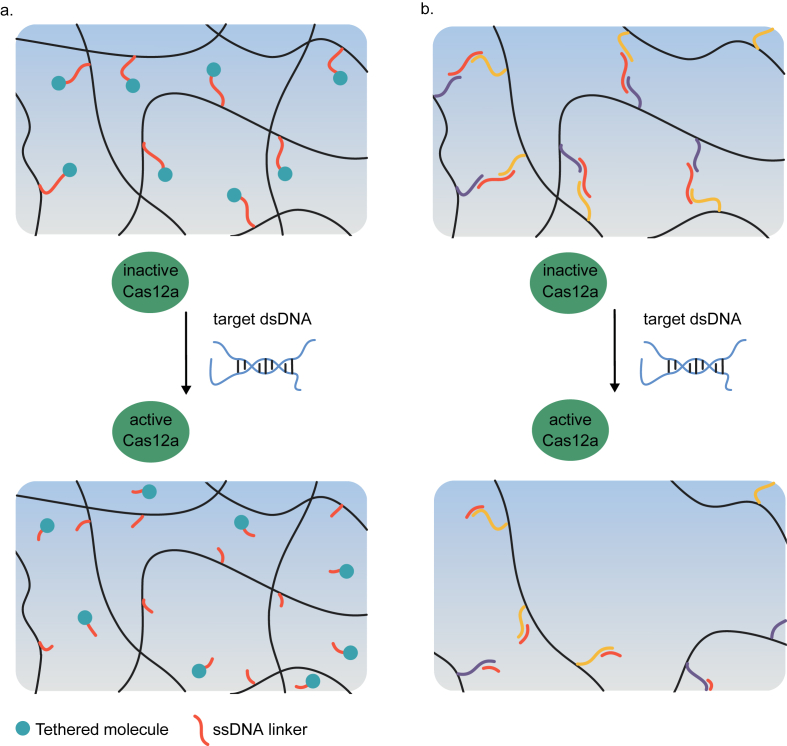
**CRISPR/Cas-responsive programmable hydrogels. a.** Release of a fluorescent dye from a hydrogel in response to double-stranded DNA (dsDNA). A fluorescent dye was coupled via single-stranded DNA (ssDNA) to a PEG-based hydrogel. Inactive, gRNA-loaded Cas12a was further added. The addition of a target dsDNA cognate to the gRNA induced non-specific ssDNA nuclease activity leading to the release of the dye. **b.** Hydrogel dissolution triggered by dsDNA. Polyacrylamide functionalized with ssDNA sequences was crosslinked to a hydrogel by complementary ssDNA oligonucleotides. Addition of gRNA-loaded Cas12a and a cognate dsDNA induced non-specific ssDNA nuclease activity, the cleavage of the crosslinker ssDNA and the dissolution of the hydrogel [170,171].

The Collins group recently pioneered a new class of nucleic acid–responsive hydrogels by the development of the first CRISPR-Cas–responsive material (Fig. 7) [170,171]. The system relied on *Lachnospiraceae* bacterium ND2006-derived Cas12a, that displays specific nuclease activity toward double-stranded DNA (dsDNA) sequences matching the gRNA spacer sequence, and subsequent non-specific hydrolase activity toward (ssDNA). This non-specific cleavage activity is highly efficient with approx. 1250 turnovers per second. Three different variants of Cas12a-based materials were described. In the first one, a fluorescent dye was covalently coupled via a ssDNA linker to a PEG-based hydrogel synthesized from multi-arm PEG crosslinked via thiol-vinyl sulfone chemistry. Cas12a was loaded with gRNA sequences recognizing several antibiotic resistance genes from multidrug-resistant *Staphylococcus aureus* and added to the hydrogel. The subsequent addition of dsDNA encoding the resistance genes resulted in the specific activation of Cas12a, the hydrolysis of the ssDNA linker, and the release of the dye with a half-life of 1–2.5 h, depending on the PEG concentration in the hydrogel. In the second configuration, a hydrogel was engineered that dissolved upon the detection of specific dsDNA. To this aim, polyacrylamide was crosslinked via ssDNA, so that the addition of gRNA-loaded Cas12a and a trigger dsDNA resulted in the degradation of the DNA-based hydrogel crosslinks leading to gel dissolution. This dsDNA-induced dissolution was used to release previously embedded gold nanoparticles or human primary peripheral blood mononuclear cells on command. In the third application, a dsDNA-sensitive electrical fuse was constructed. To this aim, carbon-black conductive nanoparticles (CB-NPs) were incorporated into a DNA-only material by melting dsDNA and cooling it in the presence of CB-NPs. The resulting material was applied on interdigitated electrodes and lyophilized. Placing the electrode in a reaction tube containing Cas12a loaded with gRNA and upon addition of a cognate dsDNA, nuclease activity of Cas12a was induced. The subsequent degradation of the DNA-based material interrupted the conductive path leading to an increase in electrical resistance between the electrodes. This is a promising approach toward directly interfacing the presence of a molecular analyte with an electrical readout for further processing or data transmission. The main advantage of these CRISPR-Cas–responsive gels is their flexibility. By simply exchanging the gRNA sequence, the materials can be engineered to specifically detect dsDNA sequences of choice [171].

The above-described molecule-responsive hydrogels can be triggered in a temporally resolved manner by applying or withdrawing the inducer molecule. However, a spatially resolved manipulation remains difficult because of the diffusion of the inducer molecule within the material. This limitation can be overcome by materials responsive to light. To synthesize hydrogels whose structural and functional properties can be manipulated by light, protein-based photoreceptors have been used to reversibly crosslink chemical polymers or proteins. For an overview of such materials, see Table 8.

**Table 8:** Overview of protein photoreceptor-based hydrogel materials with light-tunable mechanical properties.

Photoreceptor	Description	Performance	Reference
UVR8	UVR8 was coupled via a protein–peptide (TIP-1/WRESAI) interaction to supramolecular nanofibers. Reversible gel–sol phase transition by light-controlled UVR8 dimer–monomer transformation.	Dark: gel state, G’ = ~10–100 Pa. UV light: Dissolution within 0.5 h. Reversion to gel state within 2 h in the dark. At least 2× reversible.	[158]
LOV2	LOV2-Jα was enzymatically functionalized on N- and C-termini with reactive azides and covalently coupled to 4-arm PEG tetrabicyclononyne (20 kDa) via strain-promoted azido–alkyne cycloaddition (SPAAC). Reversible stiffness modulation by light-controlled adjustment of crosslinker length.	Dark: G’ = ~880 Pa. Blue light (470 nm, 10 mW cm^−2^): Decrease in G′ to ~810 Pa (t_1/2_ = 0.9 s). Reversion in the dark with t_1/2_ = 35 s. At least 43× reversible.	[179]
EL222	EL222-SNAP was covalently coupled via benzylguanine maleimide into a collagen hydrogel. Reversible stiffness modulation by light-controlled EL222 monomer–dimer transformation.	Dark: E = 196 Pa. Blue light (450 nm, 10 mW cm^−2^): Increase in E to 239 Pa within 10 min. Reversion in the dark within 10 min. At least 2× reversible.	[173]
Dronpa145 N	Dronpa145 N was covalently coupled via SpyCatcher/SpyTag interaction to a protein polymer ((GB1-SpyCatcher)_3_). Reversible gel–sol phase transition by light-controlled Dronpa145 N tetramer–monomer transformation.	Violet light (400 nm): gel state, G’ = ~100–300 Pa. Cyan light (500 nm, 9 × 3 W LED): Dissolution within 2 h. Reversion to the gel state within 0.5 h upon violet light illumination (9 × 3 W LED). Reversible for 1.5 cycles.	[174]
Dronpa145 N	Dronpa145 N was covalently coupled via Michael-type addition to 4-arm PEG maleimide (20 kDa). Reversible gel–sol phase transition and reversible stiffness modulation by light-controlled Dronpa145 N tetramer–monomer transformation.	Violet light (405 nm): gel state, G’ = ~300–2000 Pa Cyan light (505 nm, 263 mW cm^−2^): 20-fold softening within 14 min. Reversion to 90% of stiff state within 15 min upon violet light illumination (273 mW cm^−2^). At least 5× reversible.	[176]
CarH_C_	CarH_C_ was genetically fused both at the N and C terminus via elastin-like polypeptide (ELP) linkers to SpyTag or SpyCatcher thus forming a protein polymer. Unidirectional gel–sol phase transition by light-induced monomerization of CarH_C_ tetramers.	Dark: gel state, G’ = ~660 Pa. White light (30 klux LED, equals ~4 mW cm^−2^): Dissolution within 20 min.	[178]
Cph1	Cph1 was covalently coupled via Michael-type addition to 8-arm PEG vinyl sulfone (40 kDa). Reversible stiffness modulation by light-controlled dimerization and monomerization of Cph1.	Red light (660 nm): G’ = ~2400 Pa Far-red light (740 nm, 0.3 mW cm^−2^): Decrease in G′ to ~1350 Pa with t_1/2_ = 86 s. Reversion to stiff state upon red light illumination (0.4 mW cm^−2^) with t_1/2_ = 78 s. At least 144× reversible.	[157]

G’, storage modulus; E, elastic modulus.

The Wang and Yang groups used the *A. thaliana* UVR8 (Fig. 6c) receptor to crosslink protein nanofibers to a hydrogel [158]. To this aim, protein nanofibers were formed from the self-assembling peptide Nap fused to the tax-interacting protein-1 (TIP-1). In addition, UVR8 was fused to the hexapeptide WRESAI that specifically binds to TIP-1. In this configuration, UVR8 homodimerization resulted in nanofiber crosslinking and the formation of a hydrogel. However, upon UV-B irradiation (300 nm), the UVR8 dimers dissociated, leading to a gel-to-sol transition [158]. Similarly, the Inoue group used the iLID and Sspb proteins, that dimerize upon blue light illumination [172]. By producing multimers of both proteins in cells and subsequent illumination, the formation of intracellular hydrogels was observed. By fusing the multimerizing proteins to an RNA-recognition motif, mRNA could be sequestered within the material. The authors apply this principle for the synthetic reconstitution of intracellular stress granules [172]. A similar design concept was followed by the Wilson group, conjugating the blue-light photoreceptors EL222 to collagen hydrogels [173]. To this aim, cysteine residues in collagen hydrogels were covalently functionalized with maleimide-benzylguanine (the SNAP-tag ligand) while EL222 was fused to SNAP-tag. Mixing both, resulted in the covalent binding of both. In this configuration, illumination with blue light resulted in EL222 dimerization and an increase in the hydrogel crosslink density leading to a higher stiffness. However, in the dark, EL222 spontaneously dissociated and the hydrogel returned to the initial soft state [173].

**Fig. 8:**
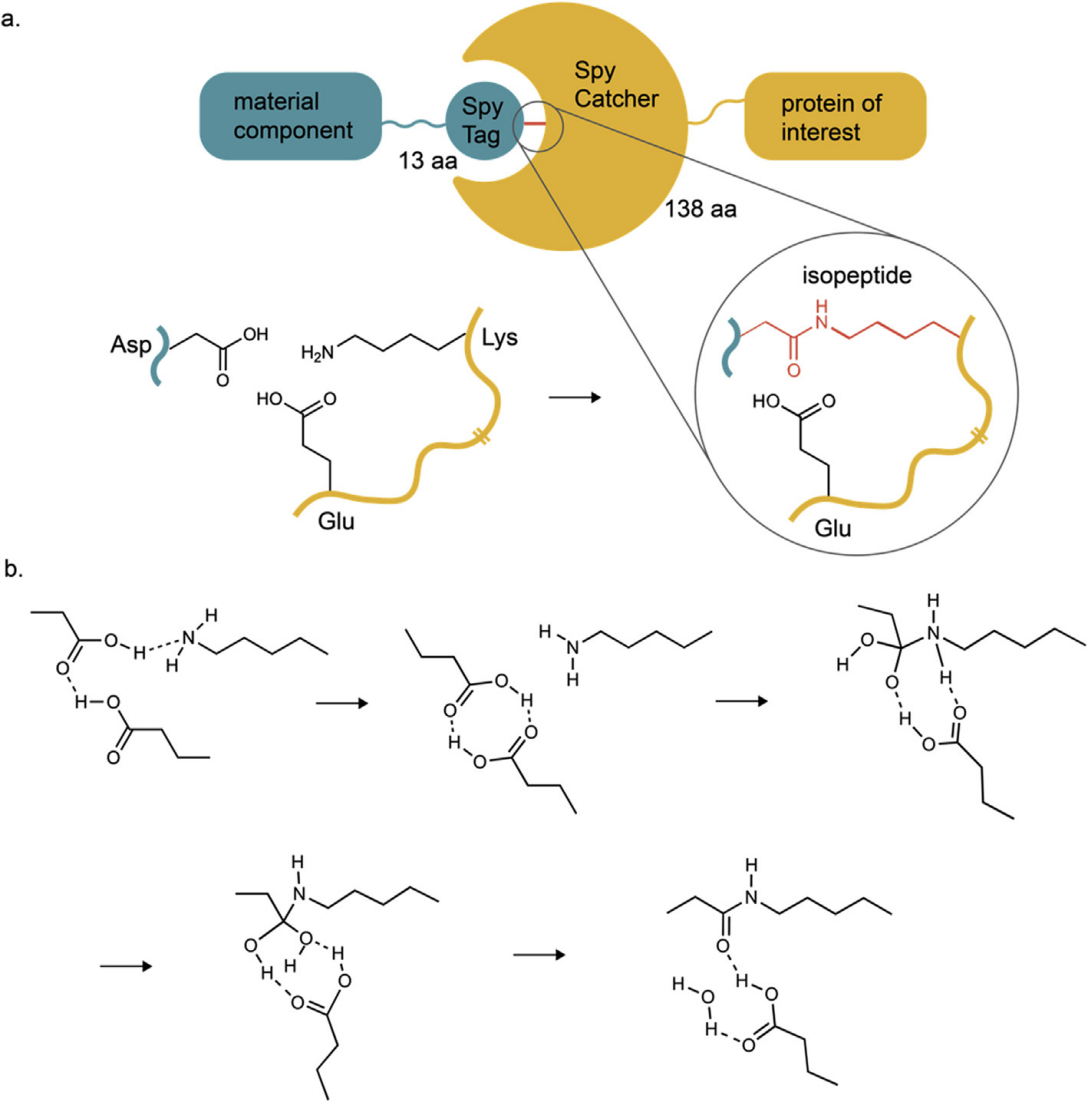
**The SpyTag/SpyCatcher system for the spontaneous formation of covalent isopeptide bonds. a.** Coupling a SpyCatcher-fused protein of interest to a material functionalized with SpyTag [175]. **b.** Reaction mechanism for the formation of an isopeptide bond between the side chains of lysine in SpyCatcher and aspartic acid in SpyTag catalyzed by glutamic acid [177]. aa, amino acids.

In another study, the Li group synthesized a hydrogel based on the fluorescent protein Dronpa145 N [174]. Dronpa145 N forms homo-tetramers in response to violet (400 nm) light and monomerizes upon illumination with cyan (500 nm) light. To form a light-responsive hydrogel, Dronpa145 N was fused to the SpyTag peptide to allow specific covalent binding to the SpyCatcher protein [174]. The SpyTag-SpyCatcher system is a modular system, where both components spontaneously bind covalently via an isopeptide bond, thereby offering many applications in bioconjugation (Fig. 8) [175]. Here, the addition of a trimeric SpyCatcher protein resulted in trivalent Dronpa145 N molecules that were crosslinked to a hydrogel upon illumination with violet light, whereas cyan light triggered Dronpa145 N dissociation and a gel-to-sol transition. In a similar work, Dronpa145 N was tetramerized by coupling to 4-arm PEG yielding a biohybrid material that could be solidified or solubilized by illumination with violet or cyan light, respectively [176]. This material was applied as an artificial extracellular matrix to control the migration of human hepatoma SMMC-7721 cells by modulating its stiffness [176]. One challenge in using Dronpa145N-based gels is the relatively high intensity of light (approx. 300 mW cm^−2^) required for efficient switching [176].

**Fig. 9:**
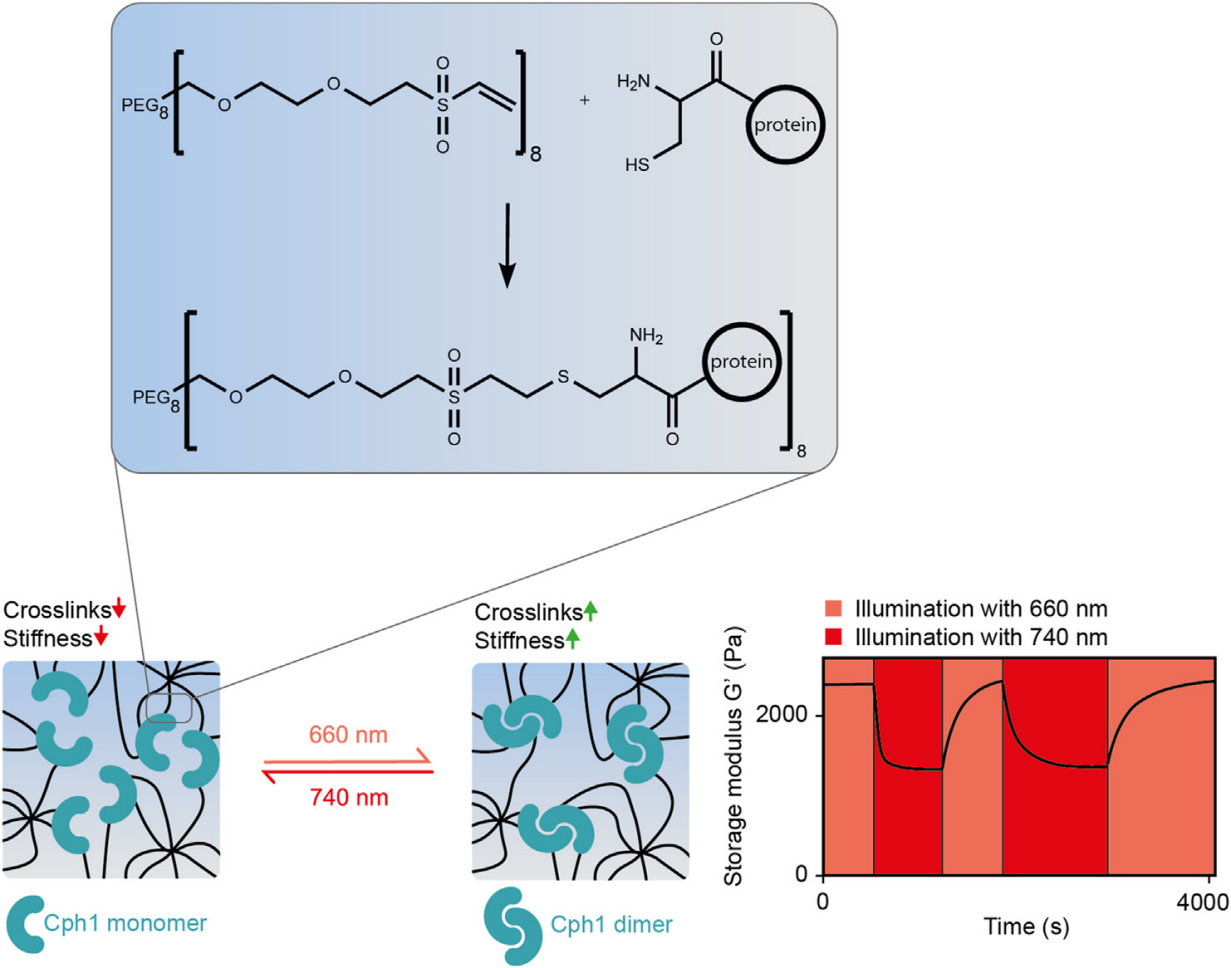
**A photoreceptor-based hydrogel with light-adjustable storage modulus.** An engineered variant of cyanobacterial phytochrome 1 (Cph1) was functionalized with a terminal cysteine and covalently coupled to vinylsulfone-functionalized 8-arm PEG. Cph1 dimerizes under 660 nm and dissociates under 740 nm light. Cph1 dimerization increases crosslink density and thus the stiffness of the material [157].

To overcome the limitations associated with the need for high intensities of energy-rich light that may impact the physiology of embedded cells, Hörner and Weber developed a hydrogel allowing a reversible change in stiffness in response to low (<1 mW cm^−2^) doses of red (660 nm) or far-red (740 nm) light [157]. To this aim, an engineered variant of the N-terminal photosensory domain of cyanobacterial phytochrome 1 (Cph1) was fused to a tandem RGD sequence to allow integrin-mediated cell attachment. The protein was further engineered with a C-terminal cysteine for coupling to eight-arm vinyl sulfone-functionalized PEG via a Michael-type addition. Alternating illumination with 660 nm or 740 nm light resulted in the reversible modulation of the hydrogel's stiffness (Fig. 9). This gel was used to expose mesenchymal stem cells (MSCs) to cycles of alternating stiffness with different frequencies to identify the signaling pathways by which the cells discriminate between short, transient mechanical stimuli, and long-lasting stimuli that may eventually lead to cell differentiation. The gel was further applied to control the migration of primary T lymphocytes by local modulation of the gel's stiffness [157].

Similarly, the Sun group developed light-responsive hydrogels by flanking the green light photoreceptor CarH on the N- and C-termini with elastin-like peptides that were subsequently concatenated to linear CarH multimers by the SpyTag-SpyCatcher system [178]. Consequently, CarH tetramerization in the dark triggered hydrogel formation, whereas illumination with green light dissociated the photoreceptor tetramers, causing a gel-to-sol transition. This gel was applied for the light-responsive release of embedded mammalian cell lines and primary cells. Of note, CarH-based hydrogels cannot be reversibly modulated because the green light–mediated dissociation of CarH dimers is mediated by the irreversible photolysis of the CarH chromophore AdoB12 [178].

Following an alternative design, the DeForest group used the light-inducible conformational change of photosensitive LOV2 to synthesize a blue light–responsive hydrogel [179]. In an elegant approach, the N- and C-termini of the protein were consecutively and selectively labeled with azide groups via a dual-enzymatic approach using an N-myristyl transferase and a sortase, respectively. The dual-azide functionalized protein was subsequently used to crosslink 4-arm bicyclononyne-functionalized PEG via strain-promoted azido-alkyne cycloaddition (SPAAC) to form a hydrogel. The storage modulus G’ of the resulting gel was reversibly modulated between approx. 810 and 880 Pa by triggering conformational changes of LOV2 induced by consecutive blue light–dark cycles [179].

The Wegner group recently used photoreceptors to induce narcissistic self-sorting and self-assembly of colloidal materials in response to multichromatic light. To this aim, 2 μm polystyrene particles functionalized with Ni^2+^-NTA were coupled to the His-tagged red and blue light receptors Cph1 and VVDHigh, respectively. Both receptors form homodimers upon illumination with light of the cognate wavelength, and dissociate under far-red light or in the dark, respectively. Exposure of a mix of both particles to red or blue light resulted in clusters of Cph1-functionalized or VVDHigh-functionalized particles, respectively. Upon co-illumination with red and blue light, both VVDHigh-functionalized and Cph1-functionalized particles self-sorted and self-assembled into distinct clusters. Cluster formation was further shown to be reversible by incubation in the dark or under far-red light [180].

##### 2.5.8.2 Toward information-processing materials

A key driver of synthetic biology was the development of synthetic circuits that process input signals according to fundamental computational operations. For example, circuits were engineered to perform signal amplification, digital and analog computation [181], information storage [182], counting of input events [183], or oscillations [15,184]. These circuits were based on molecular switches such as stimulus-responsive activator or repressor proteins and corresponding promoters that were wired to each other according to circuit topologies inspired from electrical engineering and control theory. Beyond applications in synthetic biology, such molecular circuits can serve as blueprints for the development of materials that process specific input information according to fundamental computational operations [185]. In a recent study, the DeForest group synthesized hydrogels that perceived three input signals, processed these signals according to different Boolean algebraic operations and, depending on the result of such computation, dissolved or remained stable [186]. As input sensors, peptide-based crosslinkers were used, containing a matrix metalloprotease 8 (MMP-8) cleavage site, a disulfide bond (cleavable by reducing agents), or an *ortho*-nitrobenzyl ester group (cleavable by UV light) in different configurations. The peptides flanked on both termini by azide groups and used to crosslink four-arm PEG tetrabicyclononyne by SPAAC chemistry. By placing the stimulus-responsive moieties in the peptides in different parallel (AND-gate type) or serial (OR-gate type) configurations, hydrogels were synthesized that dissolved only upon a specific combination of input stimuli. By covering all hierarchical YES/OR/AND combinations, 17 distinct crosslinkers were synthesized, each exhibiting a unique logical input–output relation [186]. These materials were used to release the cytostatic drug doxorubicin only upon the detection of MMP-8 activity AND reducing conditions, a combination indicative of tumor environments, thus ensuring a target site-specific delivery of the drug.

**Fig. 10:**
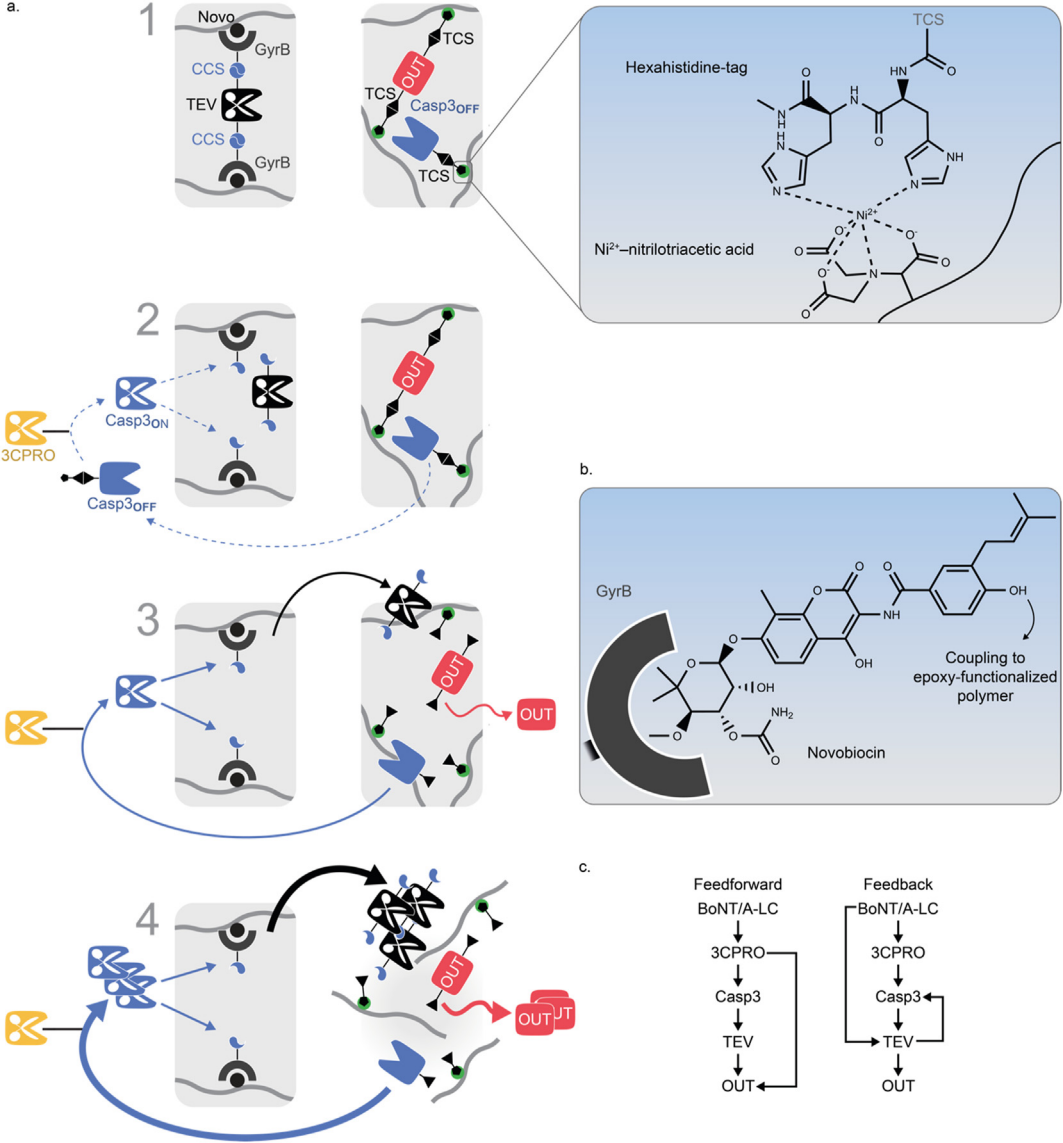
**An information-processing materials system for signal amplification. a.** Mode of function of the material. (1) Starting configuration. In the first module, the TEV protease is bound N- and C-terminally via a caspase cleavage site (CCS) to the protein GyrB. GyrB binds to novobiocin covalently coupled to epoxy-agarose (see **b** [165]). In the second module, the output protein (OUT) is fused to two His-tagged TEV cleavage sites (TCS). The His-tags crosslink Ni-NTA-modified polyacrylamide to a hydrogel (see inset). The second module further contains an engineered caspase 3 variant (Casp3_OFF_ [187]) that is inducible by the 3C protease (3CPRO). Casp3_OFF_ is linked via a TCS and His-tag to Ni-NTA-polyacrylamide. (2) Triggering the system. As Casp3_OFF_ is non-covalently bound to the polymer, small amounts of the protein diffuse into the buffer. The system is triggered by the addition of 3CPRO leading to the activation of Casp3_OFF_. Casp3_ON_ subsequently cleaves TCS and releases TEV. (3) Intermodule communication. Released TEV diffuses to the second module cleaving TCS and releasing the output protein as well as more Casp3_OFF_. (4) Signal amplification and output. The released Casp3_OFF_ is activated by 3CPRO, releasing more TEV by CCS cleavage to fuel the positive feedback loop. The TCS cleavage-mediated release of OUT reduces the crosslink of the polyacrylamide hydrogel leading to its dissolution. Using the red fluorescent protein mCherry as OUT yielded a readout observable by the naked eye. **c.** Detection of botulinum toxin A (botox, BoNT/A-LC) by signal-amplifying materials systems. Similar to the concept in a different material modules were interconnected according to feedforward and feedback topologies to amplify the initial botox signal to a readout visible by the naked eye [166].

While this example relied on linear information processing, Wagner and Weber recently described a hydrogel system for the sensing and non-linear signal amplification of input stimuli based on positive feed-forward and positive-feedback loops (Fig. 10) [165]. In this study, four different switch mechanisms were used: (i) protease-sensitive linkers for crosslinking polyacrylamide to a hydrogel, (ii) protease-sensitive linkers to couple a protease or an output protein to a polymer network, (iii) inactive proteases that were activatable by cleavage with another protease [187], and (iv) small molecule-responsive affinity-based linkers to immobilize and competitively release proteins to/from a polymer network (for example, the immobilization of GyrB-tagged proteins to novobiocin-functionalized polymer). Three proteases with orthogonal target specificity were used: the TEV protease, the human rhinovirus 3C protease, and caspase-3. In an iterative design-build-test-learn approach, the topology of the system was optimized with regard to higher sensitivity and faster response time. Key to this optimization was the use of a quantitative, ODE-based mathematical model. By this *in silico* approach, different network topologies were evaluated to identify configurations with the desired properties. The model predictions were subsequently experimentally validated yielding a final signal amplification system suitable to detect nM to μM concentrations of the antibiotic novobiocin. In this setup, the initial molecular input signal was amplified via forward and feedback loops leading to the dissolution of a hydrogel, a readout that could finally be detected by the naked eye (Fig. 10a and b) [165,188]. In a similar approach, protease-based feedback and feedforward loops were engineered in a polymer material for the detection of the botulinum neurotoxin A (botox, BoNT/A-LC). Also, in this configuration, the signal resulting from low nM concentrations of botox was amplified to result in the dissolution of a hydrogel for detection by the naked eye (Fig. 10c) [166].

**Fig. 11:**
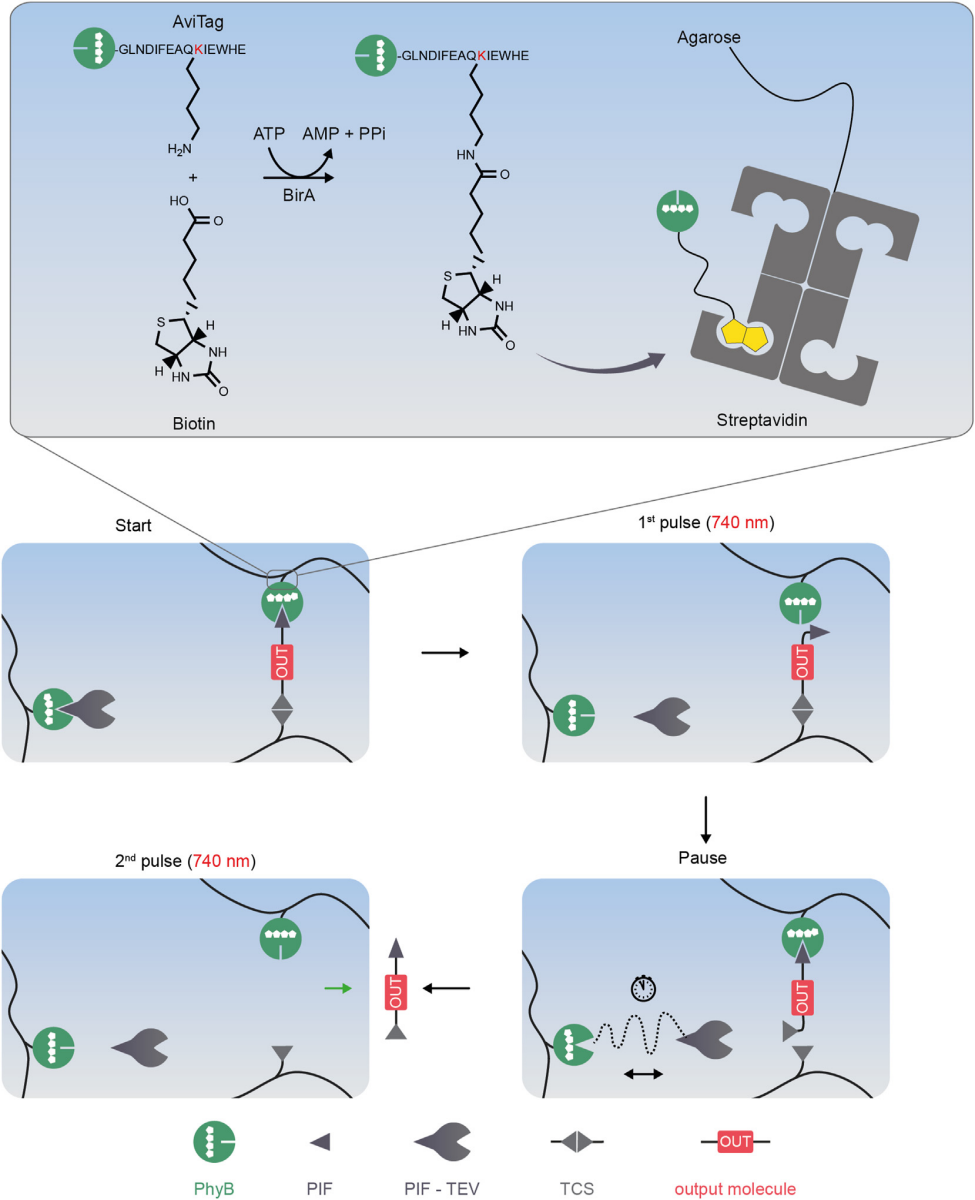
**A hydrogel that counts the number of light pulses.** The system consisted of two modules. The first one contained an agarose-based polymer functionalized with the *A. thaliana* red light photoreceptor PhyB. Under illumination with 660 nm light, PhyB bound the phytochrome-interacting factor PIF, whereas both proteins dissociated under 740 nm light. PIF was further fused to the tobacco etch mosaic virus (TEV) protease. The second module comprised the output protein (OUT) fused to an agarose polymer via two bonds, one formed by a peptide containing a cleavage site for the TEV protease (TCS) and the other by a PIF-PhyB pair. Upon illumination with a first pulse of 740 nm light, PIF-TEV was released while OUT remained bound via the TCS-peptide. In the subsequent pause between the two 740 nm pulses, released PIF-TEV diffused to TCS and cleaved the peptide, while PIF-OUT reassociated with the PhyB-functionalized hydrogel. Thus, only the second pulse of 740 nm light released OUT by dissociating the PhyB-PIF bond [156].

In the examples of the above-described hydrogels, different chemical, enzymatic, or physical cues were simultaneously applied to change the materials' properties. Such parallel, stimulus channels compare to parallel ports in PCs, where many parallel wires (for example, 25 wires for printer ports of older PCs) were used to transmit information. These parallel ports were gradually replaced by serial ports able to transmit the information through significant less wires (for example, 2 signal wires for USB 2.0). Key to reducing the number of wires was the encoding of the signals in pulses where the temporal, serial sequence of the pulses encoded the information. To enable similar, serial information-processing in materials, the Weber group designed a hydrogel system able to decode the number of input pulses applied [156]. In a first implementation, the system was designed to discriminate between one and two light pulses. The system's design was derived from pulse counter concepts in electrical engineering as well as from a synthetic gene network capable of counting the number of chemical input pulses [183]. The final hydrogel system was configured to release a protein cargo only upon the detection of two subsequent pulses of far-red light (Fig. 11) [156]. To sense the light input, the *A. thaliana* red light photoreceptor phytochrome B (PhyB) was deployed that binds to the phytochrome-interacting factor PIF6 in response to red (660 nm) light. However, upon illumination with far-red (740 nm) light, both proteins dissociate. As a means to detect the temporal encoding of the input signal, a diffusion-based delay in signal processing was incorporated. To this aim, the release of TEV protease (release of TEV-PIF6 fusion from immobilized PhyB upon the first far-red light pulse) was spatially separated from the location where it finally hydrolyzed its substrate (one of the two linkers used to couple the output protein to the polymer). The output protein was further fused to PIF6 to bind PhyB-coupled polymer. As a consequence, the output protein was released only upon (i) completion of the diffusion-controlled TEV-mediated cleavage of the linker AND (ii) the occurrence of the second light pulse (Fig. 11) [156]. Similar to the signal-amplifying hydrogel described earlier, a quantitative ODE-based mathematical model was applied to efficiently predict the parameter space in which the light pulse-counting system was functional. The model predictions were experimentally validated and the system was further extended to release phytoene desaturase (CrtI) after the first light pulse and lycopene cyclase (CrtY) after the second light pulse to consecutively catalyze the two-step biochemical reaction from 15-*cis*-phytoene via all-*trans*-lycopene to all-*trans*-beta-carotene [156].

**Fig. 12:**
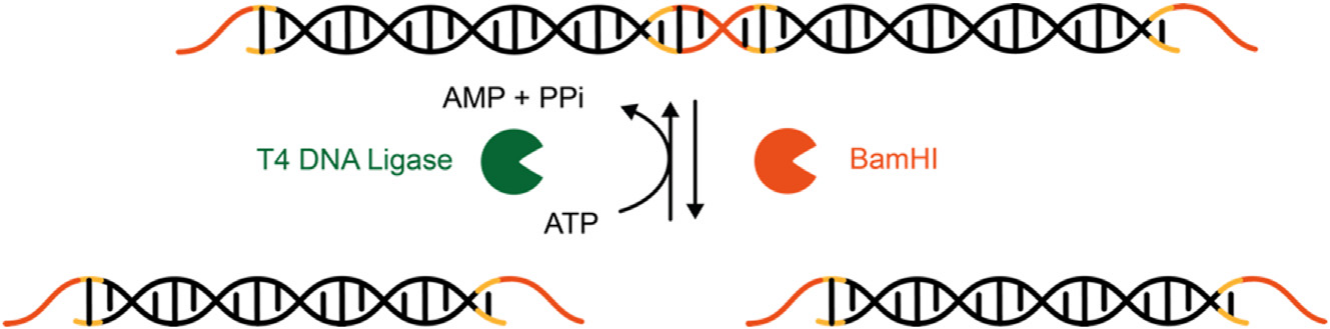
**Programmable out-of-equilibrium DNA material.** Double-stranded DNA was incubated with a BamHI nuclease that cleaved the DNA at a specific recognition site. The simultaneously added T4 DNA ligase joined both DNA fragments while consuming ATP. Thus, the steady state amount of uncleaved double-stranded DNA depended on the relative ratio of nuclease to ligase as well as on the availability of ATP. By adjusting the amount of ATP initially added to the system, the lifetime of the steady state could be programmed [189].

In a compelling recent study, the Walther group explored the synthesis of out-of-equilibrium soft matter with programmable dynamic steady states based on enzymatic reactions [189]. As material, a DNA sequence was chosen that contained a recognition site for the nuclease BamHI. To start the system, BamHI and a phage T4-derived DNA ligase were added (Fig. 12). BamHI cleaved the DNA, which was subsequently rejoined by the ligase using ATP as an energy source. Thus, the state of the material was dependent on the relative activities of both enzymes, and consequently on the supply of ATP. For example, when gradually increasing the ATP concentration from 0.1 to 1 mM, the lifetime of the dsDNA material was gradually increased from 1 to 10 days. Similarly, the addition of ATP to an energy-exhausted system resulted in a pulse-like, transient formation of dsDNA where the pulse shape and duration were dependent on the ATP amount added as well as on the incubation temperature affecting enzymatic rates [189]. In a follow-up study, the approach was extended by the concept of transient, cooperative multivalency to allow fuel-driven encapsulation, self-assembly of colloids, as well as the non-equilibrium transient narcissistic self-sorting of colloidal particles. Given the modularity of the approaches as well as the availability of many different restriction enzymes, this study enables generic access to non-equilibrium soft matter systems with programmable and adaptive dynamics in 4D [190].

## 3 ELMs

ELMs have been defined as engineered materials composed of living cells that form or assemble the material itself, or modulate the functional performance of the material in some manner [4]. Another way to characterize this field is by the term ‘Materials Synthetic Biology’ defined by integrating engineering principles from synthetic biology and materials science to redesign living systems as dynamic and responsive materials with emerging and programmable functionalities [30]. In this section, we focus on living materials according to these definitions with emphasis on materials relying on engineered cells. Accordingly, in this section, we first review approaches with a focus on engineering cells to *form* a desired material (Section 3.1), then we describe recent work on cells engineered to assemble materials (Section 3.2). In the third part, the focus will be on materials with specific *functions conferred by engineered cells* (Section 3.3).

### 3.1 Living materials formed by engineered cells

Here, we review how cells can be engineered to build materials. Two main approaches have been followed: (i) the engineering of cell–cell interactions to build cell-only materials, (ii) the engineering of cells to produce a composite material consisting of cells and cell-produced extracellular matrix.

#### 3.1.1 ELMs formed by engineered cell–cell interactions

Connecting cells to each other by genetic and/or chemical routes provides a direct access to cell-based ELMs. The Riedel-Kruse group developed an interesting approach to creating multicellular bacterial structures by displaying nanobodies or their cognate antigens on the surface of bacteria [191]. Mixing of both bacteria types resulted in three-dimensional structures. Interestingly, by varying the nanobody-antigen pairs, by using cells with different shape (rod, spheroid, filamentous), and by changing the relative ratio of the constituent cell types, different three-dimensional arrangements were obtained such as co-aggregation, phase separation, differential adhesion, or sequential layering [191].

**Fig. 13:**
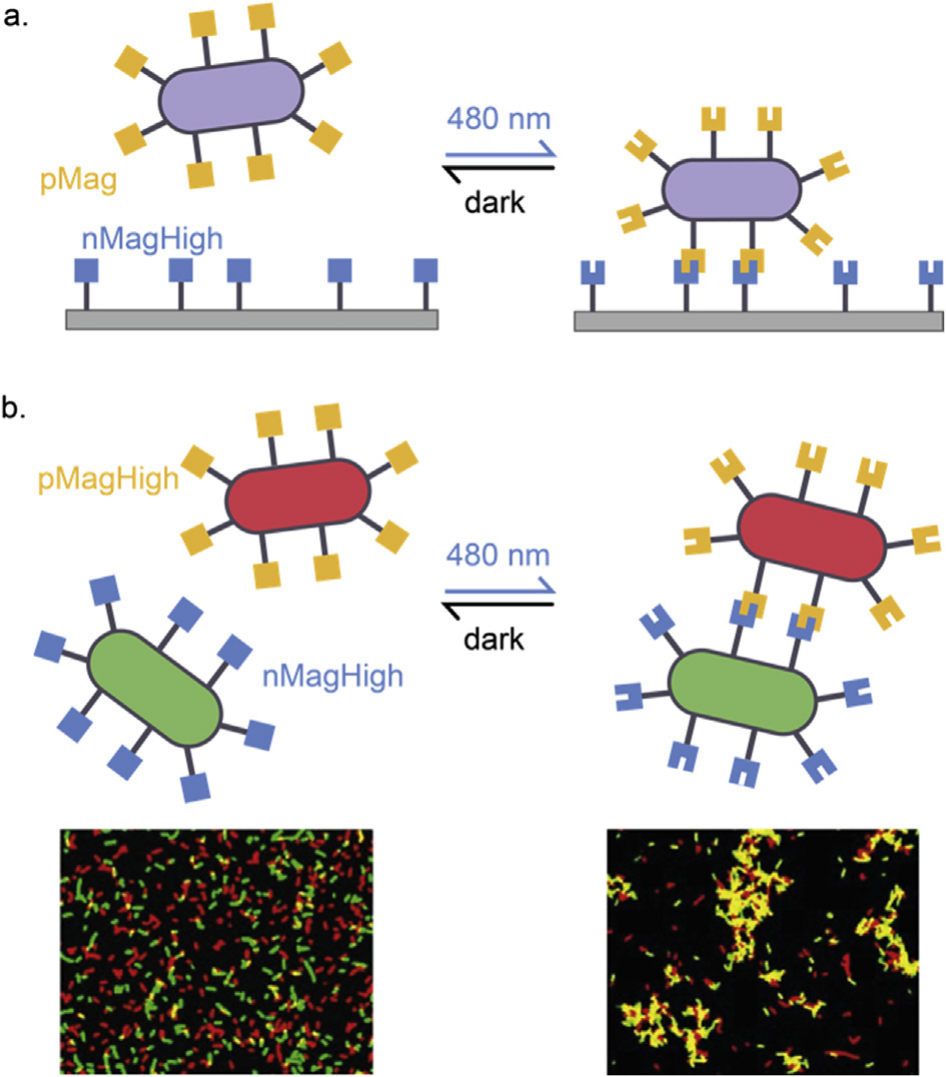
**ELMs based on engineered cell-matrix and cell–cell interactions. a.** Light-induced surface patterning with bacteria. *E. coli* cells were engineered to extracellularly display the blue light photoreceptor pMag. Furthermore, the cultivation surface was modified with the blue light receptors nMagHigh. Upon blue light illumination, pMag and nMagHigh heterodimerized leading to surface immobilization of the bacteria in illuminated areas [192]. **b.** Optically controlled formation of cell assemblies. *E. coli* cells were engineered to display either pMagHigh or nMagHigh on their surface. The cells were further engineered to produce a red or green fluorescent protein for visualization. Upon blue light illumination, pMagHigh and nMagHigh heterodimerized leading to the formation of cell assemblies [194]. Reprinted with permission from ACS Synth. Biol. 9, 1169–1180. Copyright (2020) American Chemical Society.

To gain enhanced spatial and temporal control of the formation of multicellular structures, optogenetics approaches were developed to locally trigger adherence of cells. In a first step, optogenetic tools were devised to trigger adherence of cells to surfaces. These approaches have further been extended to control cell–cell interactions. The Wegner group recently pioneered an optogenetic approach for dynamically controlling the assembly of cells on surfaces [192]. They used the protein pair nMag/pMag that dimerizes when irradiated with blue light and dissociates in the dark. The constitutive display of pMag on the surface of *E. coli* cells allowed cells to attach to a surface coated with nMag upon blue light illumination (Fig. 13a). When illuminating the cells through a photomask, spatially patterned structures of surface-bound cells were obtained [192]. A similar approach of optically controlling cell attachment was recently performed in mammalian cells by the Weber group [193]. To this aim, a PIF variant optimized for secretion (PIF^S^) was inserted into an extracellular loop of human integrin β3 in different cell lines (HEK-293T, HeLa, MCF7). The cell suspension was added onto a glass surface functionalized with PhyB. Upon illumination with red light, the cells bound the PhyB-functionalized matrix via the PIF^S^-engineered integrin whereas illumination with 740 nm light reversed the binding thus leading to cell dissociation. When locally illuminating with red or far-red light, cellular structures could be patterned onto the surface. It was further shown that attachment via the engineered integrin triggered the formation of focal adhesions and the activation of pathways involved in mechanosensing such as the MAP kinase- or the hippo-pathways [193].

Beyond surface patterning, optogenetic switches have further been applied by the Wegner group for controlling the assembly of three-dimensional cellular structures [194]. Engineering the surface of *E. coli* with either the nMagHigh or pMagHigh photoswitches allowed the optically controlled aggregation of the cells (Fig. 13b). In such assemblies, quorum sensing and biofilm formation were increased because of the higher proximity of cells to each other as compared to cultures without photoswitchable proteins. The formation of aggregates and their size could be influenced by changing parameters such as exposure time, light intensity, and cell density [194]. This approach of creating 3D cell clusters in response to light has also been extended to mammalian cells. The same group engineered human breast carcinoma MDA-MB-231 cells to either express the blue light photoreceptor Cry2 or its interaction partner CIBN on their surfaces. Illumination with blue light resulted in the formation of multicellular assemblies that could subsequently be dispersed by cultivation in the dark. Such blue light-switchable cell–cell interaction was suggested for scaffold-free bottom-up tissue engineering or to study biological processes where cell–cell interactions play a pivotal role [195].

In an intriguing study, the Mano group recently showed that mammalian cells are also able to form desired three-dimensional structures such as fibers without the need for genetic or chemical modification but simply by providing a sophisticated cell culture environment [196]. In their work, they placed a hydrophilic, wettable line surrounded by superhydrophobic areas in a culture dish. Subsequently, a suspension of undifferentiated human adipose tissue-derived mesenchymal stem cells (hASCs) was applied to the wettable region, whereas no liquid adhered to the superhydrophobic area. In the next step, the culture plate was inverted resulting in a hanging line of culture medium and cells similar to the hanging drop method previously used for organoid formation [197]. Driven by gravity, the cells accumulated at the bottom and started building intercellular connections. The length and thickness of these fibers could be tuned by the initial cell concentration and it was possible to grow fibers made from several different cell types together [196]. The Joshi group recently used a similar approach to synthesize stiff living materials [198]. They grew an *E. coli* mutant devoid of forming extracellular matrix proteins, pelleted the cells and casted them onto a PVDF membrane in polypropylene mold and let the biomass dry to form a stiff material. This approach was further shown to be compatible with *S. cerevisiae* or *Lactobacillus rhamnosus*. The resulting cell-only stiff material was shown to resist different solvents such as chloroform, ethanol, or hexane and also elevated temperatures up to 100 °C. Importantly, the resulting material still contained alive cells that could be used to regrow a new material. Such cell-only stiff materials were suggested as suitable material in a circular economy [198].

#### 3.1.2 ELMs formed by engineered cells and extracellular matrix

**Fig. 14:**
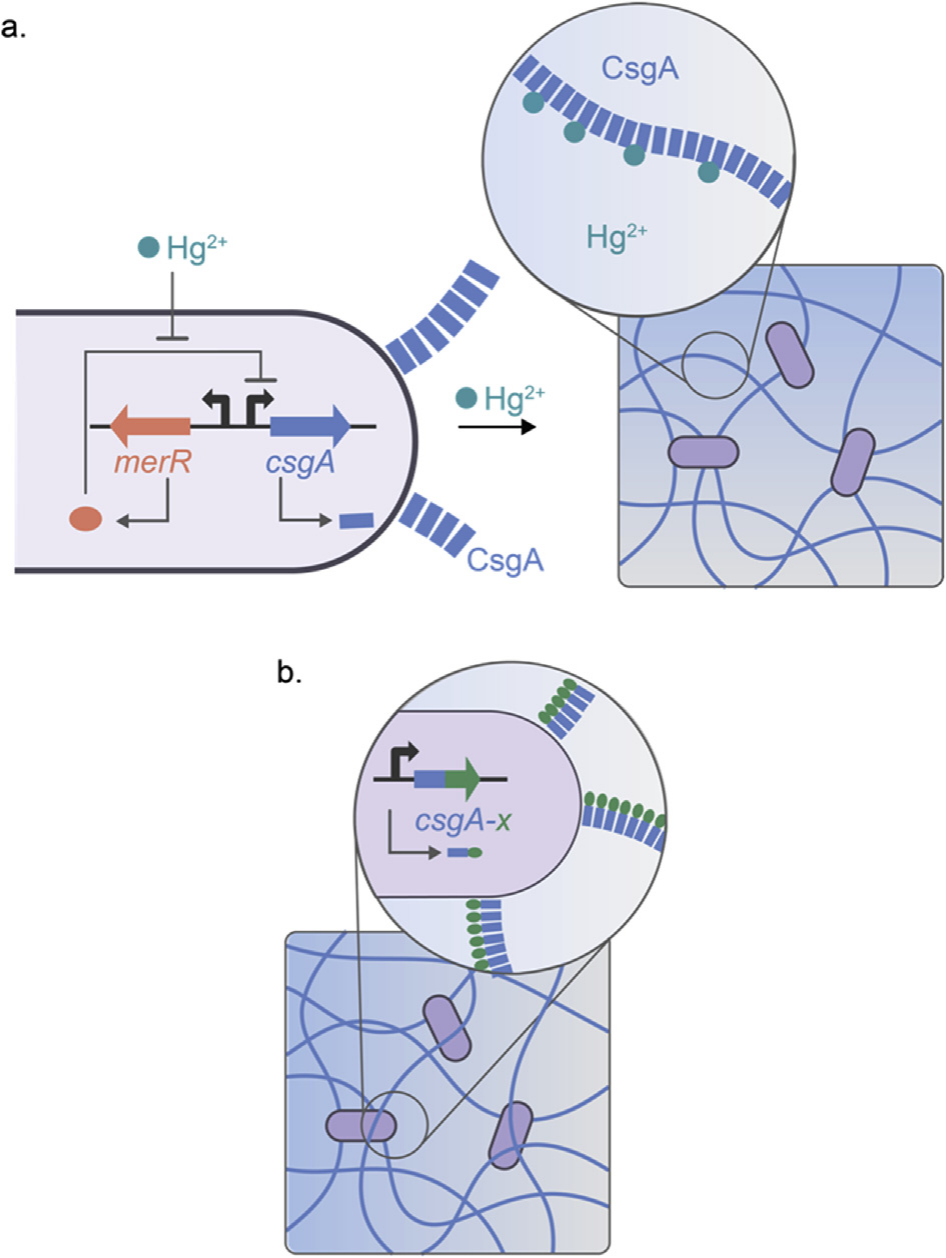
**Engineered living materials. a.** An engineered living material for bioremediation. *E. coli* cells were engineered to produce the CsgA protein under control of a mercury-inducible promoter. When cultivated in the presence of Hg^2+^, the MerR repressor protein was inactivated leading to the production of CsgA. CsgA was secreted where it polymerized to form an extracellular matrix. As curli naturally binds Hg^2+^, the heavy metal was sequestered by the forming biofilm material [199]. **b.** Engineering curli biofilms. By genetically fusing CsgA to a peptide or protein of interest, curli-based biofilm materials with desired functionality can be produced.

Many bacterial species naturally form a biofilm by producing a protective extracellular matrix composed of saccharides and proteins and, to a smaller extent, nucleic acids. In the past few years, efforts toward designing materials based on such natural biofilms have led to the development of ELMs with various interesting properties suitable for therapeutic but also environmental and industrial applications. Recent research has focused on curli, a protein-based amyloid nanofiber that represents the main proteinaceous structural component of biofilms formed by *E. coli*. Besides producing and purifying curli for further processing, curli production by bacteria is used to create biofilms as living materials. The production of curli-based biofilms has been targeted by synthetic-biological approaches to engineer ELMs for biomedical, environmental, or electronic applications. For example, the Joshi group developed an ELM for applications in bioremediation [199]. In this study, they engineered *E. coli* with a mercury-inducible promoter configured to express the curli constituent CsgA. Thus, upon detection of mercury, biofilm formation was induced (Fig. 14a). As curli fibers have a natural affinity for mercury, the formed ELM sequestered mercury from the environment. Hence, this ELM could possibly be used for the remediation of mercury-contaminated soils [199].

Several research groups recently focused on engineering the properties of curli-based ELMs by fusing CsgA as the main component of the biofilm to different protein domains (Fig. 14b). For instance, domains with specific affinities can make the biofilm adhere to surfaces of interest. The Joshi group developed an *E. coli*-based biofilm, which specifically localized to the intestinal mucosa after oral administration to mice [200]. This was achieved by fusing the curli proteins to a mucin-binding trefoil factor (cTFF2). As the living part of the material (*E. coli*) autonomously regenerated the material, persistence of at least 5 days was attained [200]. A similar system has been leveraged for the treatment of gastrointestinal inflammation in mice [201]. The probiotic strain *E. coli Nissle 1917* was transformed with a synthetic gene expression cassette coding for the mucin-binding curli and administered to mice before, during, or after the induction of colonic inflammation. An ELM formed and remained in the colon for at least one month. This probiotic-associated therapeutic curli hybrid (PATCH) material lowered inflammation in the colon of the mouse model [201].

In addition, curi fibers have been harnessed to interface biological with abiotic materials, such as nanoparticles and quantum dots, resulting in hybrid organic–inorganic materials [202,203]. For example, the Lu group engineered curli fibers for binding to gold particles or quantum dots, giving rise to ELMs that could be used as conductive biomaterials or as capacitance-based touch switches, respectively. Furthermore, the Zhong group placed curli biosynthesis under the control of a blue light–responsive promoter to pattern ELM formation. By adding different quantum dots sequentially to the forming ELM, they created a layered material [204].

As an alternative to the aforementioned affinity-based non-covalent binding, the SpyCatcher system was used to covalently bind a variety of catalytic activities to an ELM. This could yield catalytic surfaces incorporating multiple activities for applications in the pharmaceutical industry or for water treatment [205].

**Fig. 15:**
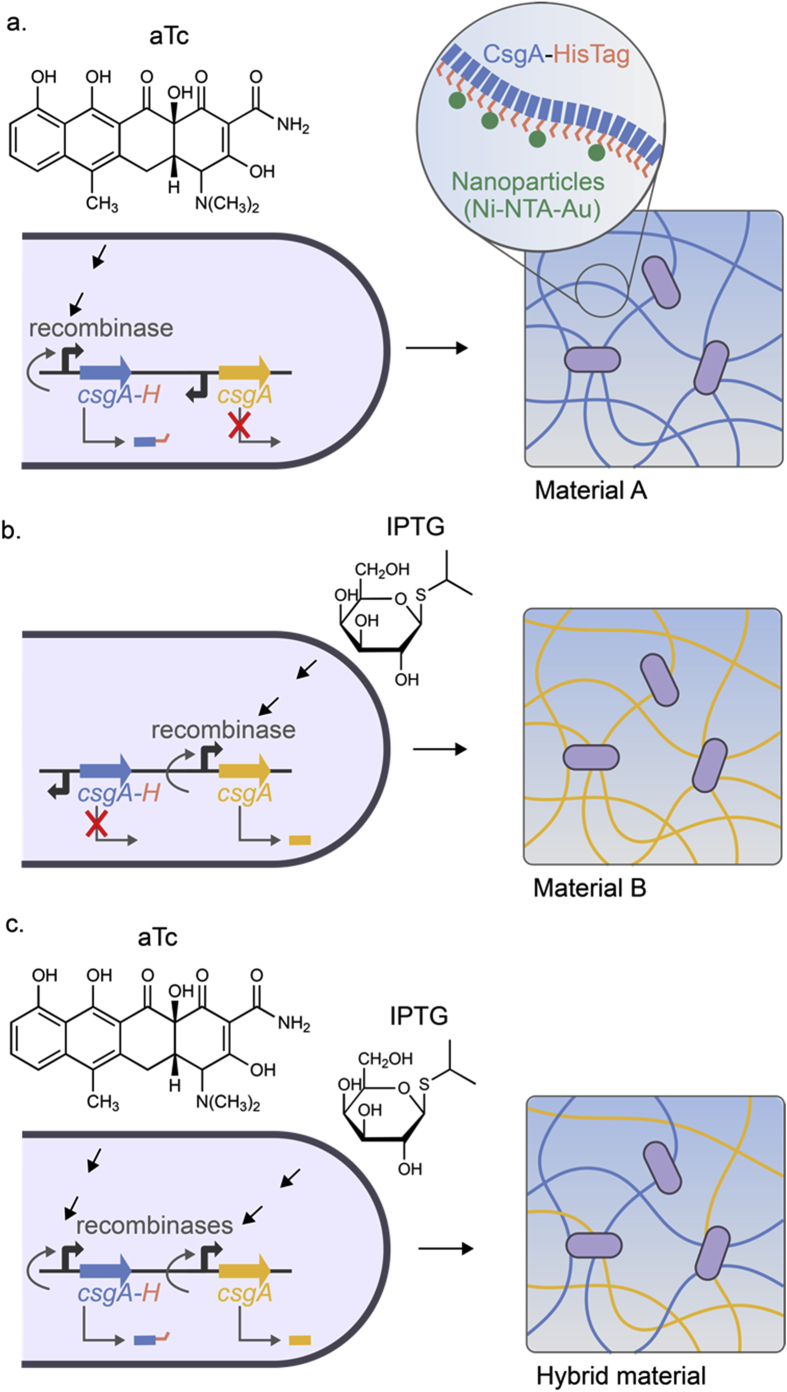
**Programming ELM composition and function.***E. coli* was engineered to produce wildtype CsgA and His-tagged CsgA under the control of two different promoters. Each promoter was placed in an inverted configuration between recognition sites of specific recombinases. The promoters controlling the production of the recombinases were inducible by isopropyl β-d-1-thiogalactopyranoside (IPTG) or by anhydrotetracycline (aTc). Thus, induction of a recombinase by the addition of IPTG or aTc resulted in the inversion of the promoter sequence to allow expression of the target gene. **a.** The addition of aTc induced curli formation based on CsgA-His-tag. **b.** Addition of IPTG induced production of wildtype curli. **c.** Upon simultaneous addition of both, a hybrid curli was produced consisting of His-tagged and wildtype curli. His-tagged curli could be coupled to Ni-NTA-functionalized molecules. Interestingly, a segregation of the two CsgA variants in the mixed gel was observed leading to wildtype and engineered curli fibers [206].

To generate biofilms with adjustable properties, the expression rates of different CsgA variants can be tuned. For example, the Seker group engineered an *E. coli* strain with two expression cassettes, one for wildtype CsgA and one for His-tagged CsgA [206]. The corresponding promoters were placed between recombination sites so that the orientation of each promoter could be inverted by inducing the expression of a corresponding recombinase. The genes for the two recombinases were placed under the control of an isopropyl β-d-1-thiogalactopyranoside (IPTG) or an anhydrotetracycline (aTc)-inducible promoter. In this configuration, the production of the recombinase that inverts the promoter for wildtype CsgA was induced by IPTG, whereas the recombinase for inversion of the promoter driving CsgA-His production was produced in response to aTc. Thus, using the same cell but using different inducers, three types of ELM could be created: wildtype curli (addition of IPTG), His-tagged curli (addition of aTc), or hybrid curli consisting of both species (IPTG and aTc, Fig. 15) [206]. An overview of molecular engineering approaches of ELMs based on curli proteins is shown in Table 9.

**Table 9:** ELMs based on engineered curli.

Development	Description	Reference
Enzyme immobilization	Curli nanofibers immobilization platform based on lipase-binding peptides for improved catalytic performance	[19]
Amyloid adhesive material	Assembly of the barnacle adhesive protein, AACP43, into amyloid-type structures	[207]
Drug delivery	Hydrogels based on engineered amyloid curli fibers, customized to interact selectively with different tissues of the gastrointestinal tract	[200]
Nanomaterial production devices	Formation of curli fibers displaying material-binding peptides for nanomaterial synthesis	[208]
Metabolic engineering	Multienzyme-assembly-cascade system for the biocatalytic production of glucosamine from chitin on amyloid fibers	[209]
Tunable biological interfaces with inorganic materials	Cell-synthesized curli nanofibers to controllably form gold nanoparticles and gold nanowires	[210]
Bioremediation	Mercury-absorbing self-assembling curli nanofibers	[199]
Biofilm-Integrated Nanofiber Display (BIND)	Platform for self-assembly of engineered CsgA fusion proteins into amyloid curli nanofiber networks	[205,211]
Engineered cellular platform	System based on inducible genetic circuits and cellular communication circuits to regulate *E. coli* curli amyloid production to achieve tunable multiscale patterning	[212]

The Voigt group selectively produced different curli variants to pattern ELMs onto textiles, ceramics, and plastic [213]. To this aim, a previously developed optogenetic system was applied to selectively trigger expression from three specific promoters in response to illumination with either red, green, or blue light (RGB vision in *E. coli* [214]). The system was configured to produce His-tagged CsgA, HA-tagged CsgA, or wildtype CsgA in response to red, green, or blue light, respectively. By local illumination with light of different color, patterned biofilms were formed with different properties in the differentially illuminated areas. Light-inducible biofilm formation was further applied for the coating of textiles. To this aim, cotton fabric was inoculated with cells engineered to form a biofilm under blue light and to produce GFP under green light. After 6 h illumination under blue light, the fabric was washed and subsequently illuminated with a green light for inducing GFP production. Accordingly, the resulting fabric showed green fluorescence when later excited with blue light. Furthermore, electron microscopy analysis confirmed the adherence of curli-based biofilms to the cotton fibers. This study thus represents a bio-based way to integrate new spectral properties into wearable textiles in response to external signals [213].

In an intriguing approach, the Zhong group has recently developed living materials fabricated via mineralization of light-inducible biofilms [215]. In their work, they coupled light-inducible bacterial biofilm formation with biomimetic hydroxyapatite mineralization. To this aim, they used a blue light-inducible promoter for the production of biofilms formed by CsgA fused to an engineered variant of mussel foot protein Mfp3S*.* Mfp3S was enriched with aspartate, lysine, and tyrosine residues, which promote nucleation, growth, and adhesion of hydroxyapatite. Using this approach, they used patterned illumination to grow a biofilm with desired shape that was subsequently mineralized. They applied this approach for the site-specific repair of defects in surfaces. At the site of defect in a polystyrene surface, they optically induced the growth of a biofilm, the fibers of which locally immobilized polystyrene microspheres. The subsequent formation of hydroxyapatite triggered a mineralization process that stabilized the patch at the site of the defect. This approach might be used to locally seal defects, whereas the optical control allows excellent spatial precision in forming the repair material [215].

**Fig. 16:**
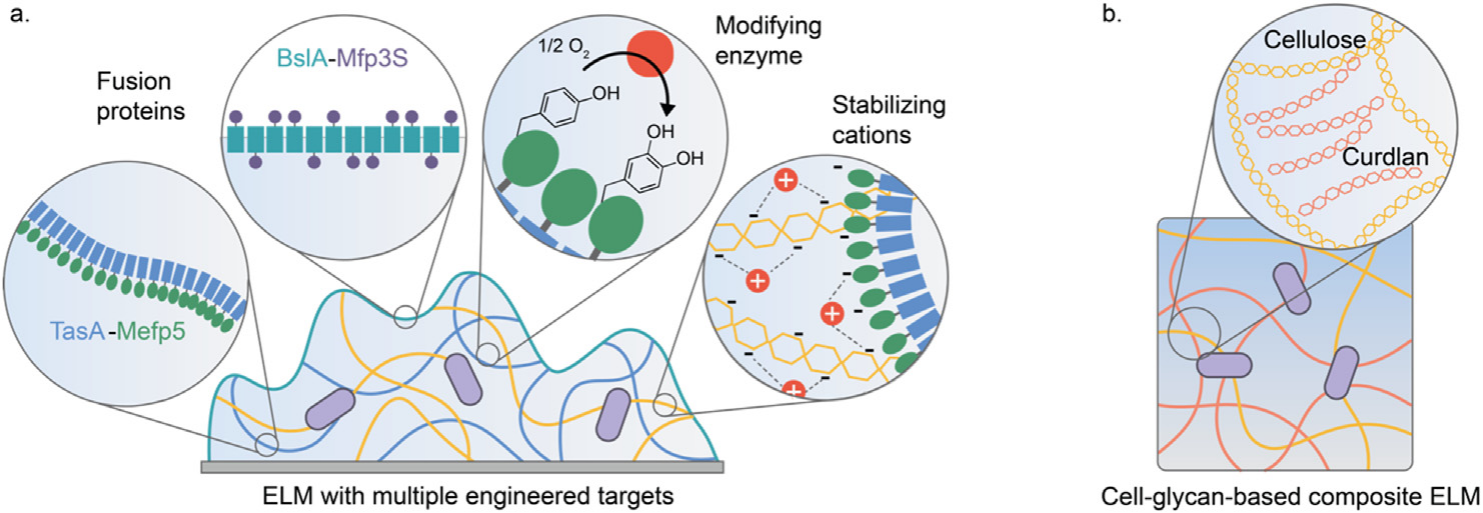
**Engineering protein and oligosaccharide-based ELMs: a.** Engineering of a living glue by multitarget engineering of *B. subtilis*-based ELMs. The biofilm proteins TasA and BslA were fused to the polyphenolic phosphoprotein Mefp5 and the mussel foot protein Mfp3Sp, respectively. The secretion of a tyrosinase converted tyrosine residues in the protein into DOPA. DOPA spontaneously oxidized to DOPA-quinone that formed covalent bonds with other molecules such as amino acids. The biofilm was further stabilized via electrostatic interactions with bivalent cations (adapted from Ref. [217]). **b.** Cell-glycan-based composite ELM. The naturally cellulose-producing organism *G. xylinus* was engineered to produce curdlan synthase. Thus, a living material was synthesized formed by the cells embedded into a matrix of cellulose and curdlan. This hybrid material showed increased hydrophobicity and water resistance compared to non-engineered materials [221].

The approach of designing ELMs by engineering biofilm properties has also been performed for other bacteria. For example, the Zhong group simultaneously engineered several extracellular components of a *B. subtilis* biofilm-based ELM yielding a waterproof glue (Fig. 16a). In this work, the extracellular amyloid peptide TasA was functionalized with the adhesive peptide Mefp5 derived from underwater-living mussels to increase biofilm adhesion. In addition, the mussel foot protein Mfp3Sp was fused to BslA, a protein that localizes at the surface of *B. subtilis* biofilms. Mfp3Sp is a previously described tyrosine-rich adhesive peptide derived from the mussel foot protein Mfp3S that forms coacervate structures with low surface energy to facilitate spreading over surfaces. Thus, Mfp3Sp enhanced biofilm adhesion while BslA prevented water penetration into the biofilm, especially in wetting conditions. Furthermore, cells were engineered to produce tyrosinase, an enzyme that catalyzes the hydroxylation of tyrosine residues in the biofilm proteins to L-3,4-dihydroxyphenylalanin (DOPA). DOPA can form bidentate hydrogen bonds with surfaces. Furthermore, DOPA oxidizes in the presence of oxygen to DOPA-quinone, a highly reactive molecule that forms covalent crosslinks to other molecules such as amino acids [216] thus stabilizing the biofilm and the attachment to the surface. Finally, adhesion was further increased by the addition of multivalent metal ions (for example, Ca^2+^, Mg^2+^, Fe^3+^) to facilitate the curing process (Fig. 16a) [217]. Similarly, the Zhong group engineered *B. subtilis*/TasA-based biofilms to yield materials with viscoelastic behaviors of hydrogels that could be fabricated into microstructures having different 3D shapes by using 3D printing or microencapsulation. The materials were multifunctional, self-regenerating, and tunable, and showed considerable fabrication processability. Based on these characteristics, the materials were suggested to be suitable as biomaterials in biotechnology and biomedicine [218].

One interesting target for the engineering of ELMs based on cells and extracellular matrix is the S-layer of *Caulobacter crescentus* as a foundation for stable, high-density 2D living materials. The S-layer forms a para-crystalline surface layer around the bacterium based on hexameric lattices of the RsaA protein. The Ajo-Frankling group functionalized RsaA with the SpyTag peptide, thus rendering the layer accessible for covalent modification with different cargo such as engineered proteins, nanocrystals, or biopolymers at a high density (1 attachment site per 288 nm^2^) [219]. Interestingly, the ligation of quantum dots to the bacterial surface did not impair cell viability and remained intact for 2 weeks. This approach may act as basis for the self-organization of soft and hard nanomaterials on a cell surface with control over 2D density, composition, and stability of the resulting ELM for a range of applications [219].

In a recent study, the same group engineered *C. crescentus* to produce a crosslinked extracellular protein matrix for living materials [220]. To this aim, *C. crescentus* was engineered to secrete via its type-1 secretion apparatus an extracellular matrix protein constructed from elastin-like polypeptides fused to a supercharged SpyCatcher variant. It was shown that this secreted protein bound covalently to a SpyTag-functionalized S-layer (see paragraph above) of *C. crescentus*. This two-strain system to secrete a synthetic extracellular protein matrix was suggested as a step toward understanding the parameters required to engineer living cells to autonomously construct ELMs [220].

The synthesis of biofilm-based ELMs is not restricted to modifying extracellular proteins. The Kondo group has developed engineered exopolysaccharide-based biofilms [221]. For example, *Gluconacetobacter xylinus*, a natural cellulose-producing bacterium, has been transformed with the *Agrobacterium* curdlan synthase gene *crdS* conferring the ability to synthesize curdlan based on uridine diphosphate glucose, a late intermediate of the cellulose synthesis pathway. This led to the formation of a cellulose/curdlan nanocomposite with increased hydrophobicity and water resistance compared to wildtype cellulose-based ELMs (Fig. 16b) [221]. A complementary strategy to engineer glycan-based ELMs is the incorporation of non-native sugars into an extracellular polysaccharide. To this aim, the Wang group disrupted the natural *de novo* synthesis pathway for exopolysaccharides and replaced it by a salvage pathway that can use sugar analogs supplied to the medium. This was applied to functionalize polysaccharides with fucose residues bearing amino, aldehyde, azido, or alkyne groups that can further be coupled to other molecules by, for example, click chemistry [222]. Alternatively, exopolysaccharides were functionalized via proteins fused to a specific binding domain. For example, the Ellis group recently functionalized bacterial cellulose with proteins fused to a cellulose-binding domain. Here, the fusion protein could directly be secreted by the cellulose-producing cells or exogenously added in purified form. Coating bacterial cellulose with antimicrobial or contaminant-binding proteins yielded materials suitable for wound dressings or for water purification, respectively. Furthermore, possible applications of coated exopolysaccharide-containing biofilms include the use as matrix for tissue engineering [54].

In a recent study, the Lu and Ellis groups developed an ELM formed from engineered microbial co-cultures of *S. cerevisiae* and cellulose-producing *K. rhaeticus* bacteria. In this co-culture, yeast was engineered to secrete different enzymes such as beta-lactamase fused to a cellulose-binding domain for stable incorporation into the resulting material. It was shown that the immobilized enzyme was retained after washing and maintained its activity after drying and rehydration of the material. In a next step, yeast was engineered to produce cellulose-modifying enzymes such as glucanases and hydrolases to modify the produced material *in situ*. By engineering yeast with light- and chemical-responsive receptors coupled to a fluorescent reporter, the material could be patterned by local illumination or acted as a sensor for chemicals, respectively. This study showcases the viability of microbial co-cultures combined with synthetic biology tools to design, grow and test living materials [223].

### 3.2 ELMs assembled by engineered cells

In this section, we review ELMs based on cells that have been engineered to modify and process materials in their environment to yield an extracellular matrix. For example, the Keitz group used the electroactive bacterium *Shewanella oneidensis* to polymerize extracellular monomers to a hydrogel [224,225]. In this process, the Mtr pathway of *S. oneidensis* directed metabolic electron flux to an extracellular metal catalyst, which generated radicals from a halogenated initiator. The radicals were exploited to start the cross-linking of exogenously added acrylate-functionalized hyaluronic acid yielding a cell-containing hydrogel. Placing components of the Mtr pathway under the control of inducible promoters further allowed the tuning of hydrogel formation kinetics [224,225]. The same group also applied *S. oneidensis* to form inorganic hybrid ELMs. Here, extracellular palladium-based nanoparticles were formed around the cells by the reduction of Pd(II) contained in the medium. Interestingly, particle synthesis rate and phenotype could be modulated by changing the availability of the outer membrane cytochrome, MtrC, or of soluble redox shuttles such as flavins [226]. In a similar approach, *Cupriavidus metallidurans*, *E. coli*, and *Clostridium sporogenes* were used to trigger polymerization of PEG methacrylate via atom transfer radical polymerization (ATRP) initiated by an iron activator generated by electron transfer [227]. In a highly interesting approach, the Alexander group used such polymerization to synthesize templated polymers that strongly bound to the organisms that had produced them [228]. This was achieved using two distinct monomers added to the cells: (i) the permanent cation, trimethyl aminoethyl methacrylate (TMAEMA), to strongly bind to negatively charged bacterial surface spots, and (ii) the zwitterionic sulphobetaine, 2-(*N*-3-sulphopropyl-*N*,*N*-dimethylammonium)ethyl methacrylate (MEDSA), to increase polymer solubility as well as to act as a spacer between the cationic building blocks. Subsequently, bacteria-mediated reduction of Cu(II) to Cu(I) via respiratory chain components initiated atom transfer radical polymerization of the monomers bound to or being in close proximity to the bacterium. This *in situ* polymerization yielded templated polymers that preferably bound bacteria that previously had initiated their polymerization [228].

Complementing such bacteria-induced polymerization strategies, the Takemoto group recently described the formation of a hydrogel crosslinked by metabolically modified murine myoblast C2C12 cells [229]. In this work, the tetra-acetylated monosaccharide *N*-azidoacetylmannosamine was supplied to C2C12 cells yielding the incorporation of this modified glycan into the cell's surface glycoproteins. Subsequently, alginate functionalized with dibenzocyclooctyne was added to initiate SPAAC-mediated covalent crosslinking of the cells to hydrogels [229]. Such hydrogels crosslinked by cells were suggested as a promising route to the generation of living cell-based materials, technologies, and medicines [229].

In several recent studies, bacteria were used to fabricate construction materials. The cement industry is a major factor of global warming (about 4% of CO_2_ emission in 2018 [230]), which has led to the development of alternative and more sustainable production processes [231]. In addition, constructions based on concrete may suffer from cracks that weaken structure over time. Initially, microcracks can appear and propagate into larger ones that inevitably have to be repaired to prolong the lifespan of the construction [232]. Living materials have the ability to self-repair and self-heal, which makes them ideal modules in civil engineering. Incorporating living components into building material would allow the on-demand growth or regeneration of materials from a parent inoculum — a feature impossible to obtain with traditional building materials. Thus, recent efforts focused on doping building materials with living organisms [233]. Table 10 provides an overview of ELMs as construction materials and compares their compressive strength to conventional materials.

**Table 10:** Comparison of physical parameter between conventional and ELM-based construction materials.

Material	Organism	Survival of organisms	Compressive strength (MPa)	Crack healing ratio	Reference
Microencapsulated bacterial spores in self-healing concrete	*Bacillus sphaericus*	61–67% (8 h)	–	95.4%	[231]
	*Bacillus sphaericus*	–	40–50	48–80%	[234]
Inoculation of a cyanobacterium in a sand-hydrogel scaffold	*Synechococcus* sp. *PCC 7002*	37% for 14 days, at 24% relative humidity	3.6	–	[233]
Human urine and MICP-based bio-brick	*Sporosarcina pasteurii*	–	2.7	–	[235]
Sandstone bio-brick	*Sporosarcina pasteurii*		2	–	[236]
Autoclaved bricks	–	–	20	–	[236]
Red clay bricks	–	–	>20	–	[236]
Compressed earth blocks	–	–	0.7–3.1	–	[236]
Sandstone	–	–	70	–	[236]
Non-facing brick	–	–	8.8	–	[235]
Face brick	–	–	14.5	–	[235]

A versatile process to synthesize ELM-based construction materials is microbially induced calcium carbonate precipitation (MICP). This process is based on the natural ability of certain microorganisms to produce calcium carbonate. Two main pathways have been applied: Ureolytic bacteria such as *Sporosarcina pasteurii* (also called *Bacillus pasteurii*), *Sporosarcina ureae*, *Bacillus sphaericus*, and *Priestia megaterium* (previously *Bacillus megaterium*) decompose urea into ammonia and carbonate ions. The released carbonate precipitates Ca^2+^ to form CaCO_3_ [235,237]. For example, the US-based company Biomason uses urea to microbially produce bioLITH® tiles in a CO_2_-neutral process [238]. Biomason is further expanding its technology for the *in-situ* solidification of the soil, for example, for the construction of landing spots for aircrafts in remote areas. As sustainable source for urea, the Randall group suggested human urine. They extracted urea from urine using a calcium hydroxide-based approach and used it in an MICP process to fabricate a biological brick with a compressive strength of 2.7 MPa [235]. This strength was larger than a bio-brick made from synthetic urea (0.9 MPa) [239] but lower than conventional face bricks (14.5 MPa) [236]. As a second approach, bacteria such as *Pseudomonas denitrificans* or *Castellaniella denitrificans* were used that precipitate calcium carbonate through denitrification [237]. In this process, nitrate is reduced while organic carbon is oxidized to generate carbonate to precipitate Ca^2+^ to CaCO_3_. In alkaline conditions, the further oxidation of NO_2_^−^, formed by the reduction of NO_3_^−^, is mostly suppressed causing NO_2_^−^ accumulation. Since NO_2_^−^ was described to inhibit corrosion by oxidation of Fe^2+^ to Fe^3+^, NO_2_^−^ accumulation could even be beneficial [237].

**Fig. 17:**
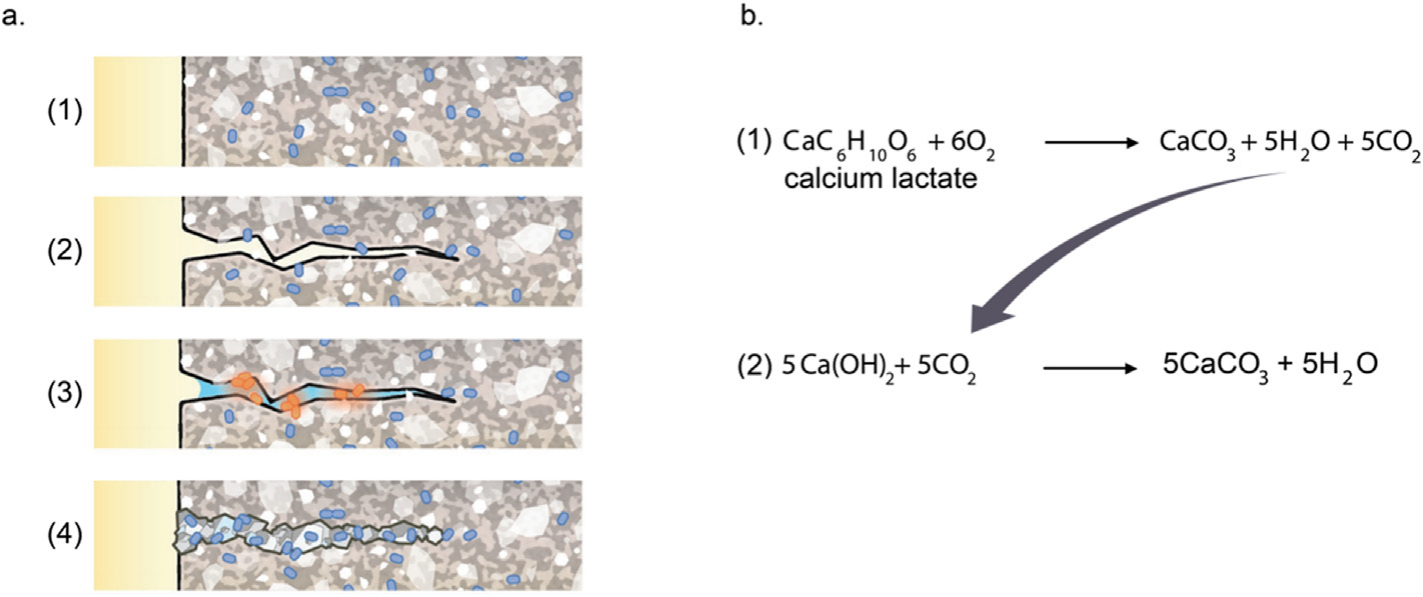
**Self-healing concrete. a.** The concrete contained dormant bacteria (1). Upon crack formation (2), oxygen and humidity entered thereby activating the bacteria (3). The activated bacteria subsequently deposit CaCO_3_ (4) and seal the crack. Reprinted with permission from Adv. Mater. 30, 1–34. Copyright (2018) WILEY-VCH Verlag GmbH & Co. KGaA, Weinheim. **b.** Formation of CaCO_3_ from organic calcium salts by bacteria [237].

A key factor that hinders the efficient use of bacteria in engineered living building materials is the difficulty of maintaining their viability in concrete. Indeed, the cement environment has quite harsh conditions such as pH > 12, high ionic strength, elevated temperatures during solidification, high mechanical shear force during concrete preparation, a dense matrix (small pores), and a lack of nutrients. To mitigate these effects, the use of bacterial spores has been suggested. Bacterial spores represent a metabolically inactive, dormant, and robust state in which the genome is strongly protected in the spore capsule. To further improve viability, different microencapsulation techniques have been developed to protect the spores mostly from mechanical stress during concrete preparation [231]. In the case of self-healing concrete, the spores are incorporated at the time of construction and hibernated there. However, upon crack formation and the subsequent entry of water and oxygen, the spores germinate (Fig. 17a). This led to the activation of the microbial metabolism and the conversion of calcium salts into calcium carbonate (Fig. 17b) [237].

In a recent study, the Srubar group pioneered the use of photosynthetic bacteria such as *Synechococcus* to form living building materials from sand [233]. *Synechococcus* fixes CO_2_ via its enzyme Rubisco. However, upon low-CO_2_ availability, O_2_ competitively inhibits Rubisco and lowers CO_2_ carboxylation efficiency. *Synechococcus* overcomes this limitation by concentrating HCO_3^−^_ from the environment within the cell while exporting OH^−^ outside of the cell. The extracellularly increased local pH further promotes CaCO_3_ precipitation. Inoculating a sand-based hydrogel scaffold with the bacterium resulted in CaCO_3_ biomineralization. By adjusting environmental parameters such as humidity, the viability of the bacteria as well as the structural properties of the material could be balanced as a function of the desired material's specifications. Interestingly, this study showed that one living material could be used as inoculum for the next generation of the material, thus providing the perspective of perpetually growing new structural materials from existing ones. Furthermore, these living building materials exhibited a compressive strength comparable to that of cementitious mortars [233].

### 3.3 Engineering cells to confer functional properties to ELMs

Engineering cells for specific functions and combining them with a suitable material environment has been shown to yield functional ELMs for a wide variety of applications. Here, we classify these ELMs according to their function. We present recent work on ELMs for analytical applications (Section 3.3.1), as living electronic devices (Section 3.3.2), for energy harvesting and conversion (Section 3.3.3), bioproduction processes (Section 3.3.4), biomedical applications (Section 3.3.5), as well as for applications in (soft) robotics (Section 3.3.6).

#### 3.3.1 ELMs for analytical applications

Synthetic biology has dissected the innate sensing mechanisms of living cells into modular genetic parts, accelerating the construction of whole-cell-based biosensors [240]. Cells can be engineered with input-specific receptors coupled to cellular reporters that are detected by analytical physical–chemical methods or even by the naked eye. This approach has enabled the development of biosensors applied for the monitoring of engineered metabolic pathways in microbial fermentations [241,242], the detection of environmental pollutants [243], or the sensing of disease cues [23,244]. Synergizing these cell-based sensors with the protecting/preserving properties of compatible polymer materials provides the opportunity for more robust biosensing modules.

Among the cell encapsulation materials used in biosensors, hydrogels stand out for providing an ideal environment for the cells to robustly sustain bioactivity over time. For example, *E. coli* biosensor cells were encapsulated into alginate beads and spread over the soil to detect and visualize the diffusion of explosives at the micromolar level [245]. The alginate gel provided a semi-liquid environment that supported cell viability for several hours in the field [245]. A further interesting material for cell embedding/encapsulation is polydimethylsiloxane (PDMS) in conjunction with a polyacrylamide/alginate hydrogel [246,247]. In the hydrogel, the covalently cross-linked polyacrylamide network was highly stretchable, whereas the reversibly cross-linked alginate network dissipated mechanical energy under deformation, leading to stretchable and tough hydrogels. With such materials, different patterns and structures could be designed by molding or 3D printing, making them interesting scaffolds for stretchable, wearable, and portable biosensors [246,247].

**Fig. 18:**
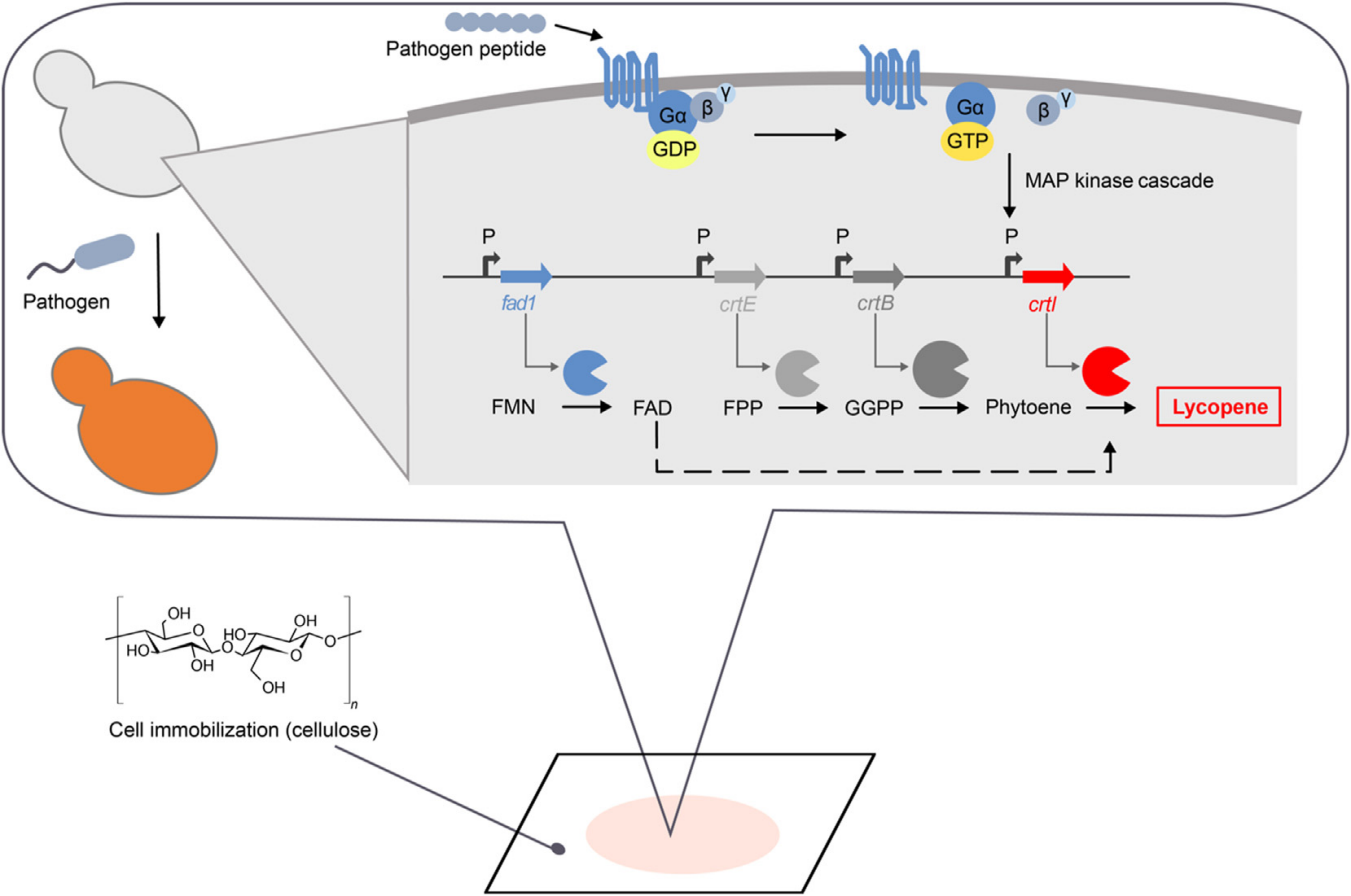
Example of a living sensor material. *S. cerevisiae* immobilized on cellulose has been engineered to express a G protein-coupled receptor (GPCR) sensitive to a pathogen-derived peptide. Upon peptide binding to the receptor, the trimeric G protein (comprising the Gα, Gβ and Gγ subunits) dissociated from the receptor and triggered activation of the MAP kinase pathway that subsequently activated transcription of the gene encoding for the enzyme CrtI. This enzyme catalyzed the conversion of phytoene to lycopene, which is a strongly red-colored pigment visible to the naked eye. Conversion of farnesyl pyrophosphate (FPP) via geranylgeranyl pyrophosphate (GGPP) to phytoene was catalyzed by the enzymes crtE and CrtB, respectively. Furthermore, the gene *fad1* was expressed for synthesizing the cofactor flavin adenine dinucleotide (FAD) from flavin mononucleotide (FMN) [248].

In addition to hydrogels, other passive materials have been used to encapsulate biosensor cells. For instance, the Cornish group engineered *S. cerevisiae* with G protein-coupled receptors (GPCRs) to sense different peptides released by pathogenic fungi [248]. The sensing process was wired to a visible readout by activating the enzymatic production of the red pigment lycopene. The designer cells were immobilized on a cellulose paper to generate a rapid colorimetric dipstick test for fungal pathogens with nanomolar sensitivity (Fig. 18) [248]. Another study described the implementation of a PDMS membrane functionalized with *Spodoptera frugipera Sf21* insect cells expressing several different odorant receptors (ORs) for the detection of volatiles [249]. The cells were engineered to co-express an olfactory system composed of silk moth pheromone receptors and an OR co-receptor (Orco) coupled to the fluorescent calcium indicator protein GCaMP. The sensing of the target volatile compound triggered calcium influx into the cell, which resulted in an increase in the fluorescence intensity of GCaMP. This artificial nose has been applied to detect mixtures of odorants including 1-octen-3-ol, geosmin, bombykal, and bombykol at the ppb level. These examples, among many others, illustrate the high potential of synergizing synthetic biology and material sciences for the design of living sensing materials with flexible inputs and outputs and improved sensitivity.

In an intriguing study, the Lu and Zhao groups recently developed magnetic living hydrogels for intestinal localization, retention, and diagnosis [250]. To this aim, they formed polyvinyl alcohol (PVA)-based hydrogels into which neodymium–iron–boron (NdFeB) nanoparticles as well as engineered cells were incorporated. By applying a strong external magnetic field, the microparticles were magnetized to generate a uniform magnetic moment across the bulk gel. The resulting hydrogel exhibited a low Young's modulus (21 kPa) making it flexible to adapt to the movements of the intestine while showing a high toughness (280 kJ m^−3^) preventing that the material was torn apart by the intestine motion. Different cell types were evaluated as cargo such as *E. coli Nissle 1917* or engineered cells acting as blood sensors (see Ref. [251] and below). When orally applying these living materials, they were shown to be retained in the intestine of mice by an externally applied magnet. Upon removal of the magnet, the hydrogels were excreted and the incorporated reporter cells were analyzed. The authors showed that the system was functional in detecting gastrointestinal bleeding in mice [250].

#### 3.3.2 ELMs as living electronics

With the emergence of the Internet of things (IoT), smart, connected devices are penetrating all areas of life. Upgrading biosensing living devices with access to information technology could significantly advance the digitalization of biomedicine [252].

**Fig. 19:**
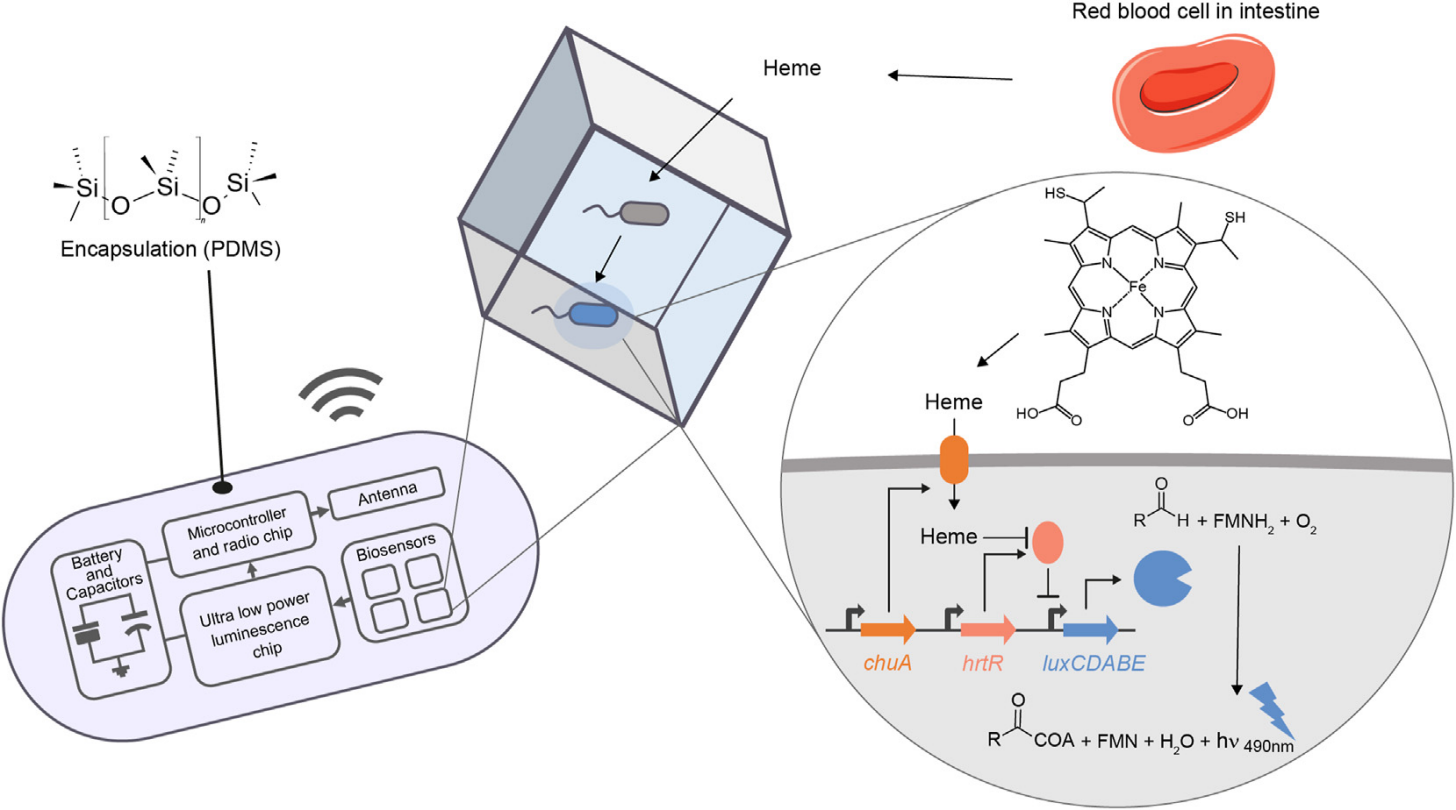
**Example of a living electronic device for the detection of blood in the intestine.** Probiotic *E. coli Nissle 1917* cells were engineered with the heme transporter ChuA and with the heme repressor HrtR for repression of the bacterial luciferase operon *luxCDABE*. In the presence of heme, HrtR binding to the DNA was prevented leading to induction of the luciferase operon resulting in a bioluminescent biochemical reaction. Bacteria were introduced into a PDMS (polydimethylsiloxane)-based microelectronic device featuring a chip for luminescence detection, a power source, a microcontroller, a radio chip, as well as an antenna. The device can be swallowed. Upon intestinal bleeding, heme was released and induced luminescence that subsequently triggering a radio signal. The signal was perceived by an external receiver such as a smartphone [253].

To integrate living diagnostics into the IoT, recent research has yielded biomolecular interfaces to connect cell fate and function to electronic devices. For example, the Lu group described an ingestible microbioelectronic device for the online detection of gastrointestinal bleeding [253]. To this aim, probiotic *E. coli Nissle 1917* was engineered with a promoter inducible by heme, a molecule contained in the blood. The heme-inducible promoter was configured to drive expression of the LuxCDABE operon, which produced a bioluminescent signal. Bioluminescence was read by a detector wired to a microcontroller and radio chip that transmitted the signal to an external receiver connected to a computer or a mobile phone. The functionality of the device was validated by orally administering the device to a porcine model of gastrointestinal bleeding. The ingested device reliably transmitted the presence of blood to the external device (Fig. 19).

Besides this cell–material interface with optical readout, a recent study used impedance measurements to detect changes in bacterial gene expression [254]. In this system, the promoter of interest was configured to drive a bacterial lysis gene for inducing cell death. The subsequently reduced cell number was detected by impedance measurement and transmitted to an electronic readout device. The functionality of the system was validated by constructing an arsenic-responsive bioelectronic device. To this aim, an arsenic-inducible promoter was used to drive the lysis gene leading to a decrease in population density, which was subsequently detected by impedance readout. The detection of 250 ppb arsenic was completed within approx. 2 hours [254]. As an alternative electrical readout, a biofuel cell was used in combination with the electroactive bacterium *Shewanella oneidensis MR-1*. In this system, the arsenic-inducible promoter was applied to drive expression of the *mtrB* gene, a major component in the bacterium's metal reduction (Mtr) pathway. The presence of arsenic was indicated by an increase in the current produced by the biofuel cell [255].

The You group recently combined a synthetic gene circuit to control pattern formation with curli-producing *E. coli* to grow electrical pressure sensors [256]. To this aim, they relied on a genetic circuit previously described by the same group [257] in which quorum-sensing signaling was used to induce circular patterns of gene expression when growing bacteria in 2D. When now growing *E. coli* on a porous membrane for supplying nutrients and not restricting growth in 3D, colonies emerged with a dome-shaped pattern of target gene expression on the top. As target gene, mCherry (for visualization) as well as hexahistidine-tagged CsgA was used. The shape of the dome was shown to be dependent on nutrient permeability and hydrophobicity of the supporting porous membrane. Following fixation of the colonies, His-tagged CsgA was loaded with gold nanoparticles resulting in an electrically conductive material. By placing one colony upside down on top of another colony, a pressure sensor was designed. By applying mechanical load, the colonies were forced together, which resulted in an increased electrical current through the materials. The authors used these sensors to control the intensity of an LED light, where a higher pressure correlated with an increased light intensity. Given the functionality of the system and the opportunity to program other growth patterns, this method was suggested as a platform to grow pressure sensors for different applications [256].

In addition to living electronics for analytical devices, cells have as well been engineered as actors that perform a desired function in response to an electrical input signal. First approaches to trigger transgene expression in response to electrical input relied on the electrochemical production of molecules to induce gene expression [258]. As an input device, a miniaturized electrochemical cell was constructed by placing an ethanol-containing agarose hydrogel within a porous Teflon tubing coupled to two platinum electrodes. Application of an electrical current triggered ethanol oxidation to acetaldehyde, which induced expression from a synthetic acetaldehyde-responsive promoter in mammalian cells cultivated in close proximity. This system has been applied to translate the amplitude or frequency of an alternating current into an adjustable genetic readout [258].

The subsequent generation of electrobiological interfaces relied on optogenetics, where electrically powered LEDs triggered optogenetic switches to induce the desired cellular response. The Ye group implemented this concept in a mammalian cell-based implant to manage diabetes in a mouse model [259]. They externally applied an electromagnetic field to induce an electrical current in the implant's coil, which powered a far-red light LED (similar to cochlear implants that are powered by external field generators). The LED was placed next to alginate/poly-l-lysine-encapsulated engineered cells to induce the activity of bacterial light-activated cyclic diguanylate monophosphate (c-di-GMP) synthase BphS. Elevated c-di-GMP levels were sensed by a synthetic transcription factor, which was based on the *Streptomyces coelicolor* BldD protein that binds its cognate DNA sequence in the presence of c-di-GMP. Binding of FRTA to the proximity of a minimal promoter induced expression of insulin or an engineered variant of glucagon-like peptide 1 (GLP) to lower blood glucose levels. The implant was administered to diabetic mice that were kept near the field generator. The field generator was connected to a control box for wireless integration into the Internet enabling the tuning of GLP production in mice via a smartphone app. This system was extended by the integration of an electronic blood glucose sensor. Connecting the readout of the blood glucose sensor via a control software with the electro-opto-genetic system for insulin production yielded a closed-loop control circuit for managing diabetes [259].

The latest generation of electrogenetic interfaces has been optimized with regard to low power consumption similar to heart pacemakers where implanted batteries have a lifetime of several years. To this aim, the Fussenegger group engineered mammalian cells with the L-type voltage-gated channel Ca_V_1.2 and the inwardly rectifying potassium channel K_ir_2.1 [251]. When placing the cells between two electrodes and applying electrical pulses, the channels opened leading to an increase of intracellular Ca^2+^ that was subsequently linked to the expression of the gene of interest. Since such gene expression-based control yielded rather slow responses due to the time needed for transcription and translation, a next generation of the system was developed allowing the real-time glycemic control in type 1 diabetic mice. There, proinsulin as well as the luciferase nanoluc were targeted for secretion in an engineered pancreas-derived beta cell line further comprising the Ca_V_1.2 and K_ir_2.1 channels. Electrical stimulation induced channel opening and an increase in intracellular Ca^2+^, which in turn induced the rapid secretion of insulin and nanoluc-loaded vesicles [251]. Such electrogenetic devices pave the way for directly connecting biological (therapeutic) devices to the IoT to facilitate the seamless integration of biological and digital health.

#### 3.3.3 ELMs for energy harvesting and energy conversion

Whole cell-based hybrid materials provide new opportunities to generate more sustainable and greener energy sources in response to global environmental challenges. Such alternative energy sources are based on, for example, microbial fuel cells containing exoelectrogenic bacteria. Electrons produced by these bacteria from the oxidation of carbon sources flow through extracellular electron transfer pathways to anodes, thereby producing electricity [260,261]. Synthetic biology strategies have been applied to improve the electron transfer efficiency of bacteria by increasing intracellular electron generation, optimizing electroconductive cytochrome systems, and promoting biosynthesis and secretion of electron shuttles [262]. In addition, 3D porous electrodes with enhanced surface area for higher bacterial loading capability [263,264] and conductive coating materials forming artificial biofilms have been explored to improve electron delivery. For example, a 3D macroporous reduced graphene oxide (rGO)/bacteria composite generated by one-step *in situ* bioreduction and self-assembly of graphene oxide (GO) with engineered *Shewanella oneidensis,* showed a 25-fold increase in the outward current (oxidation current, electron flux from bacteria to electrodes) and a 74-fold increase in the inward current (reduction current, electron flux from electrodes to bacteria) over that of the naturally occurring systems [265].

In another example, the development of photosynthetic semiconductor biohybrids has enabled the light-driven conversion of CO_2_ to chemicals with added value [266]. For example, the Yang group coated *Moorella thermoacetica* with cadmium sulfide (CdS) semiconducting nanoparticles [267]. The absorption of photons by CdS promoted electrons into the conduction band and these electrons were subsequently used to fuel the reductive acetyl-coenzyme A biosynthetic pathway to synthesize acetic acid from CO_2_.

**Fig. 20:**
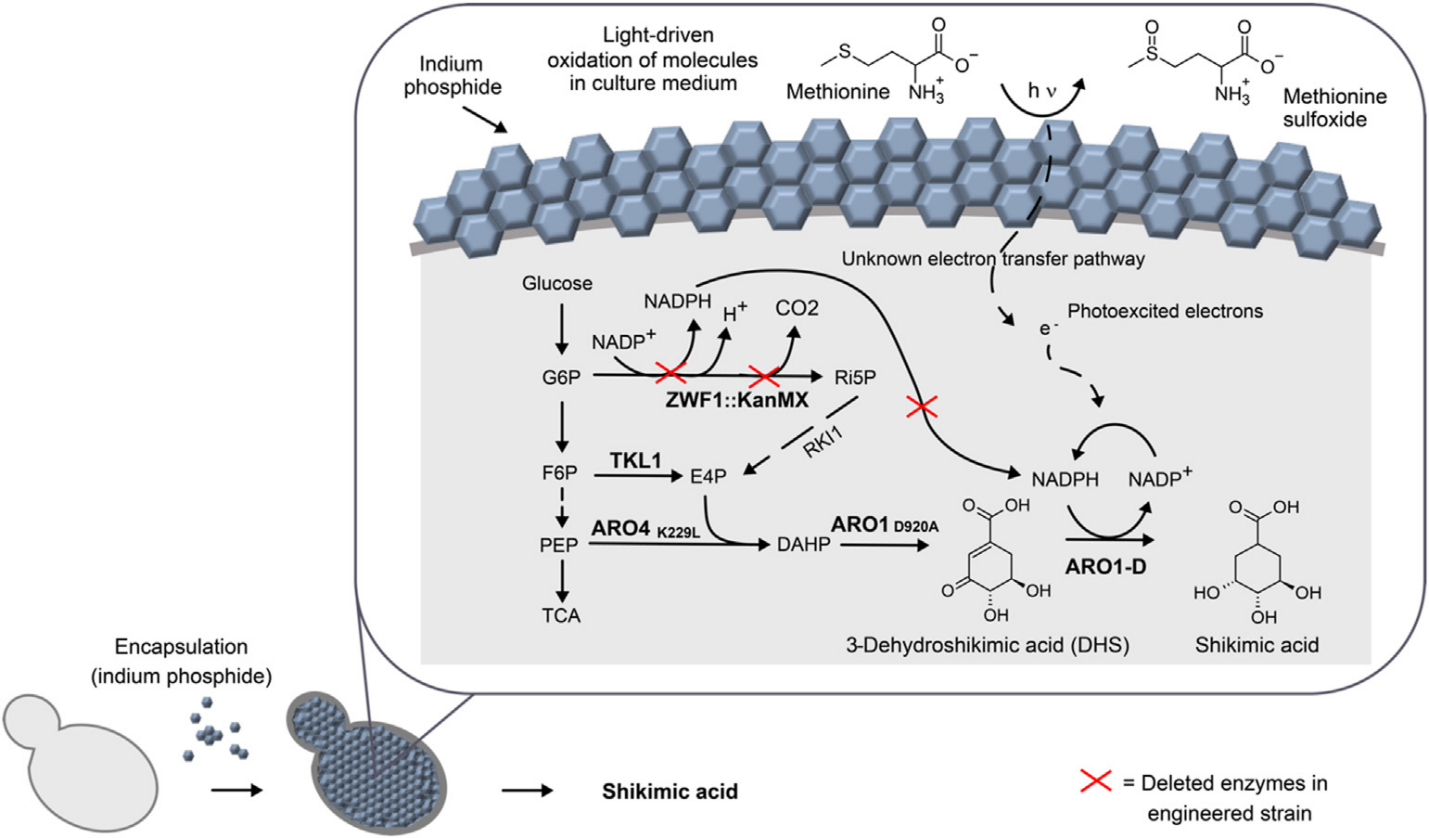
**Example of a living energy conversion material.***S. cerevisiae* coated with indium phosphide (InP) has been metabolically engineered for the light-driven overproduction of shikimic acid. To this aim, the oxidative pentose phosphate pathway has been disrupted by deletion of the enzyme ZWF1 to reduce carbon loss in the form of CO_2_. However, the shikimic acid pathway required NADPH produced in the pentose phosphate pathway. To overcome this limitation, NADPH regeneration was compensated by the photogenerated electrons from InP nanoparticles, thus providing a carbon- and energy-efficient production system for shikimic acid. Aro1-D, pentafunctional aromatic enzyme; ARO1_D920A_, mutant pentafunctional aromatic enzyme; ARO4_K229L_, feedback-insensitive DAHP synthase; DAHP, 3-deoxy-d-arabinoheptulosonate-7-phosphate; E4P, erythrose-4-phosphate; F6P, fructose-6-phosphate; G6P, glucose-6-phosphate; PEP, phosphoenolpyruvate; Ri5P, ribulose-5-phosphate; RKI1, ribose-5-phosphate isomerase; TCA, tricarboxylic acid cycle; TKL1, transketolase; ZWF1, glucose-6-phosphate 1-dehydrogenase [269].

In a similar approach, *E. coli* was engineered to produce a heavy metal-binding protein on its surface to bind CdS nanoparticles [268]. Furthermore, the cells had been engineered with an oxygen-tolerant [NiFe]-hydrogenase. Photons absorbed by CdS nanoparticles produced electrons that were transferred to methyl viologen added to the system. Methyl viologen penetrated into the bacterial cell and served as an electron carrier in the production of hydrogen catalyzed by the recombinant hydrogenase [268]. Similarly, *S. cerevisiae* coated with indium phosphide shells has been reported as a bioinorganic hybrid platform to harvest photogenerated electrons [269]. The harvested electrons maintained the cytosolic regeneration of redox cofactors and enabled the bioproduction of high value-added metabolites such as shikimic acid (Fig. 20). The combination of functionalized materials and engineered cells has a strong potential for the light-driven supply of reducing equivalents to biosynthetic pathways. However, a better understanding of the so far elusive electron transfer mechanism between the components of these living materials must further be investigated as a basis for engineering and improving the performance.

#### 3.3.4 ELMs for bioproduction

While industrial production processes are typically performed in large-scale bioreactors, two recent approaches proposed alternative, small-scale and multi-parallel production processes for small molecules and proteins based on living materials [270,271]. The Alper group used 3D printing of engineered *E. coli* or *S. cerevisiae* embedded into F127-bisurethane methacrylate (F127-BUM) hydrogels and showed that these living materials sustained the long-term (1 year) and robust production of different small molecules or peptides. Furthermore, by simultaneously combining materials with different embedded microorganisms, more complex biosynthesis pathways were implemented with each microorganism performing one part of the overall biosynthesis pathway. The authors further showed that the co-culture of embedded cells was maintained over time, whereas a suspension co-culture of the same organisms showed strong shifts in relative cell types over time [271].

Alternatively, the You group developed a living material for the continuous, integrated production and purification of proteins or small molecules [270]. They used the polysaccharide chitosan for encapsulating *E. coli*. The cells were engineered to induce cell lysis at high densities triggering the death of most cells and the subsequent release of the cell content. Growth of *E. coli* to high cell density changed pH and ionic strength inside the capsule which induced the shrinking of the chitosan capsule thus squeezing out the protein and small molecule content of the capsule while retaining the cells due to their larger size. With most of the cells lysed, pH and ionic strength within the capsule returned to the initial state and the capsules started swelling again. The resuming growth of *E. coli* triggered a new round of production, lysis and, capsule shrinking. The beauty of this approach was, that cells engineered for different metabolic reactions could separately be encapsulated and only mixed for production. Such division of labor strongly facilitated the engineering process. This concept was demonstrated by engineering seven bacterial strains each producing enzymes for one step in the fatty acid biosynthesis pathway. Combining capsule types each harboring one distinct enzymatic activity, the consortium was able to produce fatty acids [270] in a seven-step biochemical reaction.

#### 3.3.5 ELMs for biomedical applications

The emergence of synthetic biology opened the door for the development of living medication strategies for the treatment of diseases that require ongoing medical attention [244,272,273]. These strategies are based on the administration of material-embedded drug-producing bacteria or mammalian cells to provide sustained and patient-adapted delivery of therapeutic compounds. Recently, the Sankaran and Del Campo groups developed bacteria-based ELMs to produce therapeutic compounds in response to optical stimuli [274,275]. As host cells, endotoxin-free *E. coli* (ClearColi) were used, the outer membrane lipopolysaccharides of which have genetically been modified to avoid an endotoxin response in humans [276]. They transformed ClearColi with the blue light–responsive pDawn gene expression system to produce the red fluorescent protein as a model compound [274] or to express the *vio-ABCE* operon for the synthesis of the antimicrobial and antitumoral drug deoxyviolacein [275]. The engineered cells were embedded in either chemically crosslinked polyacrylamide or physically crosslinked agarose. Interestingly, the presence of acrylamide monomer at 6% w/v concentration and the free-radical polymerization did not decrease bacterial viability after encapsulation. The resulting living material sustained robust levels of drug production and release for at least 7 weeks [275]. To ensure patient safety, recent research focused on probiotic bacteria. For instance, probiotic bacteria such *as E. coli Nissle 1917* are unharmful to humans and have been engineered as hosts for several vaccine and pharmaceutical formulations [277].

Light-inducible switches to control drug production are highly intriguing, thanks to the possibility of precisely tuning drug dose and timing by modulating the light input [278]. However, clinical application might be limited by the need for instrumentation such as optical waveguides to deliver the light to the implanted material. Although commonly used implantable waveguides are based on glass or synthetic polymers that are non-biodegradable [279] and must surgically be removed after application, Del Campo overcame this limitation by the development of a biodegradable optical waveguide [280]. The waveguide was produced via extrusion printing of PEG and PEG/Pluronic precursors, and cured by *in situ* photopolymerization. The degradation times were tunable by adjusting the molar mass of the gel precursors, which were synthesized by linking PEG diacrylate with varying proportions of DL-dithiothreitol. The resulting waveguide showed optical losses as low as 0.1 dB cm^−1^ in the spectrum of visible light. The waveguide was applied to induce drug release from an optogenetically controlled ELM by delivering light across >5 cm of muscle tissue [280].

In a different approach, engineered bacteria were applied to synthesize functionalized surfaces for biomedical applications. For instance, living interfaces have been made between surface-attached *Lactococcus lactis* and human mesenchymal stem cells. To this end, the Sanchez-Salmeron group engineered *L. lactis* to produce human fibronectin III7–10 (known to support cell adhesion) on its cell wall and to deliver biological cues that support stem cell growth and differentiation [281,282]. The culture surface was functionalized with the fibronectin-displaying bacteria and used as a living matrix for stem cell culture. To avoid bacterial overgrowth of the culture, bacterial and mammalian cell growth was carefully balanced by the addition of bacteriostatic antibiotics slowing down bacterial growth while not killing them. Such approaches will potentially support the design of living materials that host symbiotic interactions between eukaryotic and prokaryotic cells and could be exploited in multiple biomedical applications such as tissue engineering.

**Fig. 21:**
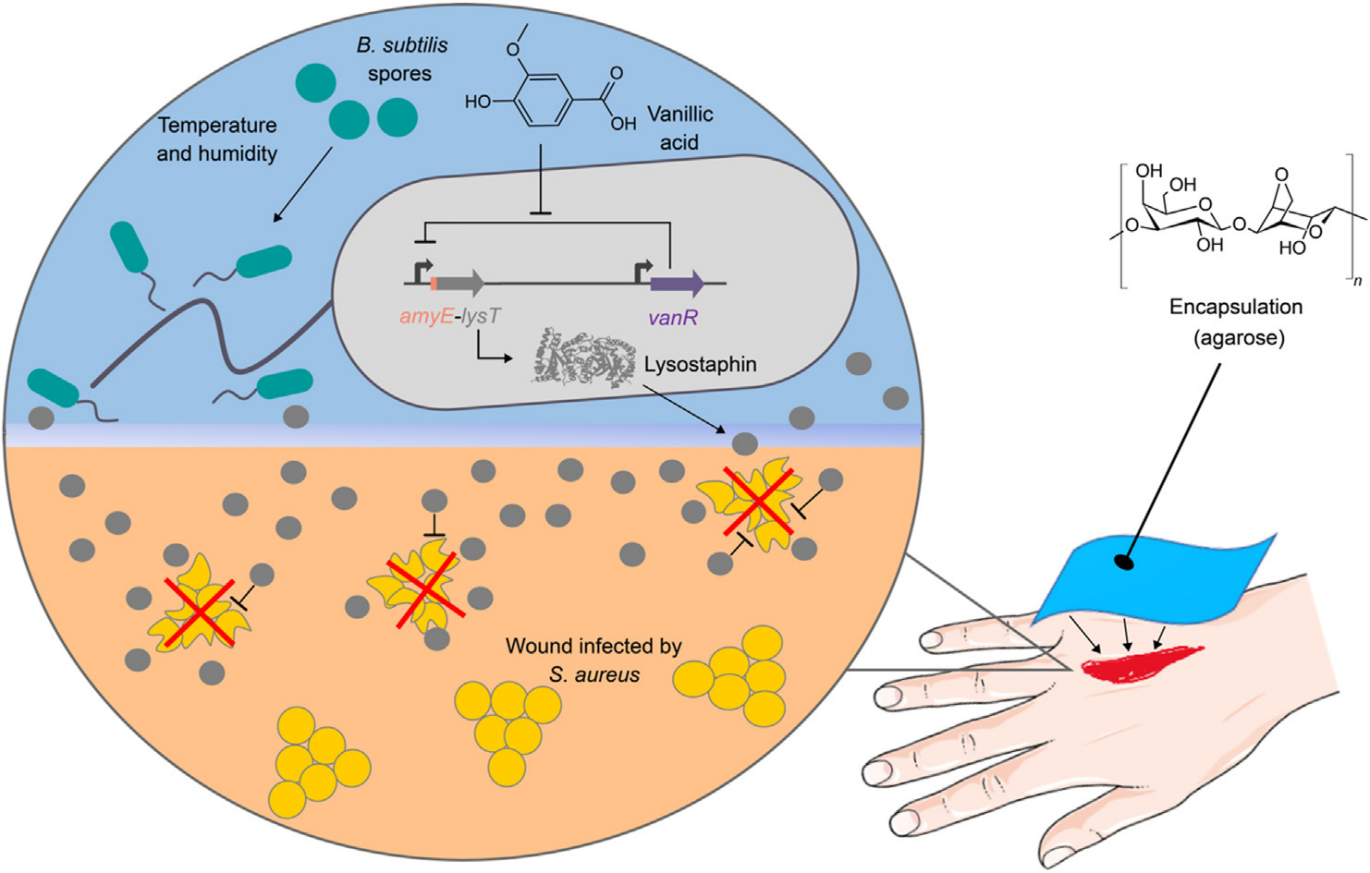
**Example of a living antibacterial wound dressing.***B. subtilis* was engineered to express the lysostaphin gene (lysT) under the control of an inducible promoter responsive to the aroma molecule vanillic acid. To secrete lysostaphin to the extracellular space, it was fused to a secretion signal derived from the *B. subtilis* alpha-amylase (AmyE). Subsequently, bacteria were induced to sporulate. The spores were mixed with agarose and 3D-printed into an agarose hydrogel patch that fitted the wound. When applied to the wound, temperature and humidity induced spore germination to yield growing bacteria. Upon addition of vanillic acid, lysostaphin was secreted to induce the death of *S. aureus* bacteria colonizing the wound. VanR, vanillic acid–responsive repressor protein [283].

Another example of a living surface with biomedical application is the development of living wound dressings with enhanced antimicrobial properties. For example, the Voigt group engineered *B. subtilis* with a vanillic acid–responsive promoter to produce lysostaphin, an antibiotic that selectively kills *S. aureus*. *B.* *subtilis* spores were encapsulated within an agarose hydrogel and 3D-printed into a bandage. Upon administration to the wound, the spores germinated due to increased humidity and temperature. In a next step, vanillic acid was added exogenously to activate a genetic switch inducing the production of lysostaphin and subsequently killing *S. aureus* (Fig. 21) [283]. Similar to the principle of self-healing living concrete above, the use of spores within hydrogels, as opposed to vegetative cells, offers several advantages including resistance to extreme desiccation and storage under ambient conditions without continuous nutrient supply. These advantages of spores can support the further development of applications where engineered cells are required to survive in fluctuating and stressful environmental conditions.

**Fig. 22:**
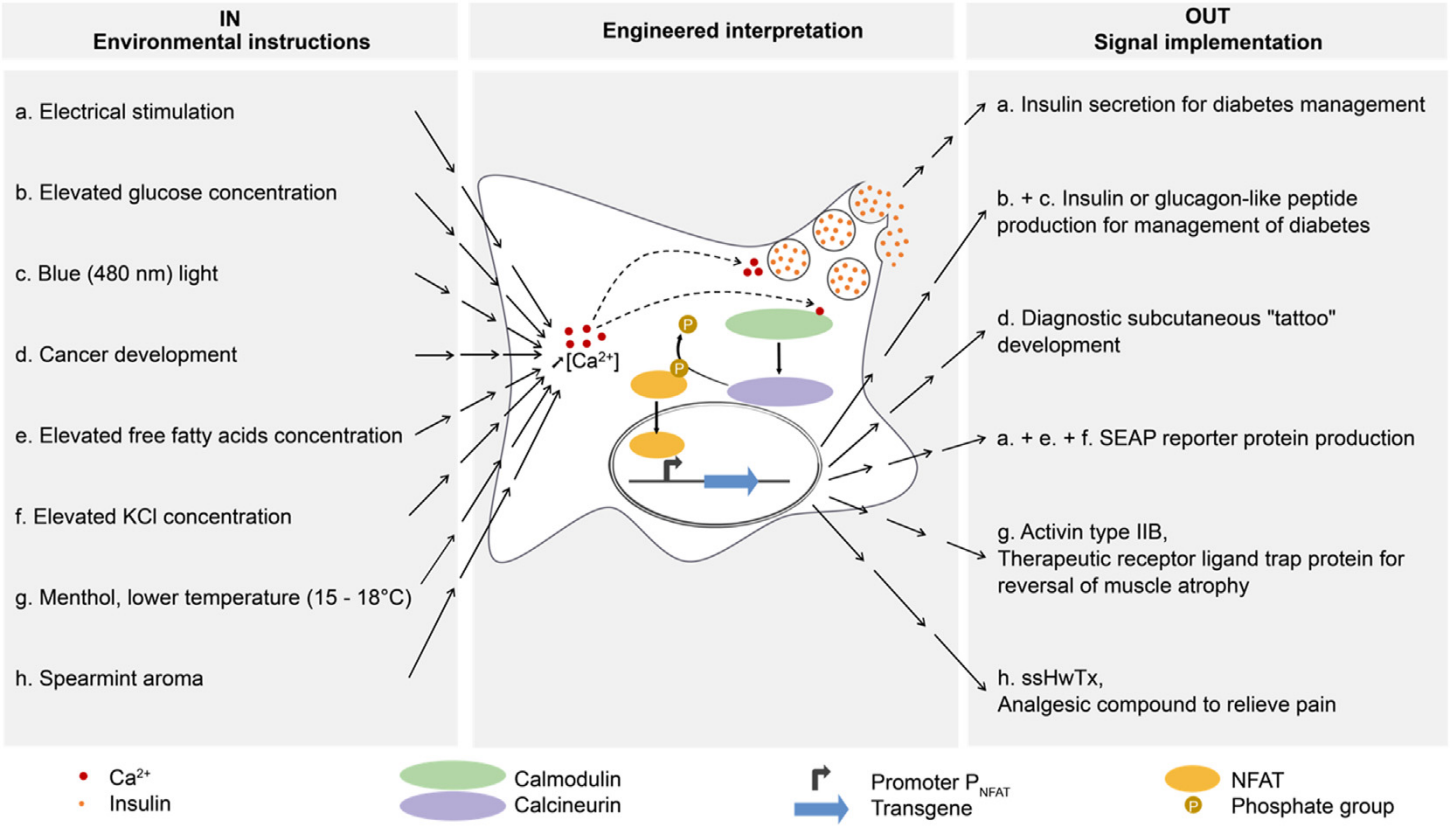
**Modular engineering of signal sensing, processing, and actuation in mammalian cells.** By genetically introducing specific receptors, different input stimuli (left panel) were wired to one central process of cellular information processing, such as an increase in the intracellular calcium concentration. Increased intracellular calcium concentration was subsequently wired to an output of choice that could be the secretion of (a.) proteins from cellular vesicles or (b.–h.) the production and secretion of therapeutic proteins. **a.** Electrically stimulated secretion of insulin for diabetes management in mice [251]. **b.** Glucose-inducible expression of insulin or the glucagon-like peptide for closed-loop controlled management of diabetes [286]. **c.** Light-inducible expression of the glucagon-like peptide for optical control of diabetes [287]. **d.** A cancer-related increase in the concentration of Ca^2+^ triggers the production of pigment-forming enzymes to yield a visible output in the form of a subcutaneous tattoo [288]. **e.** Cell-based detector for free fatty acids [293]. **f.** Detection of K^+^ by cells [290]. **g.** Menthol/temperature-controlled production of the activin type IIB protein for reversal of muscle atrophy [289]. **h.** Spearmint-inducible production of an analgesic compound [294].

The above-described approaches based on engineered bacteria allow numerous applications in biomedicine. Such approaches are complemented by encapsulated mammalian cells for therapeutic applications. The advantage of using engineered mammalian cells is that they can seamlessly be integrated into the signaling networks of the mammalian host organism. This allows the construction of ‘sentinel’ cells that are implanted into the body and continuously profile important disease parameters. Upon deviation of specific parameters from the healthy set point, therapeutic responses can autonomously be induced to counteract the disease even before clinical manifestation. Optimally, the patient would not even realize this closed-loop disease management. This approach with disruptive therapeutic potential was pioneered by the Fussenegger group having developed and refined so-called prosthetic gene networks [284,285]. For the construction of such networks, typically a natural or engineered receptor is used to detect the disease stimulus. Upon activation, the signal is transferred via a natural or engineered signaling cascade finally triggering the desired output such as the production or secretion of a therapeutic protein. Fig. 22 illustrates how such networks can be constructed and adapted to specific inputs/outputs in a modular manner. Receptors with the desired specificity are linked to generic intracellular signaling mechanisms such as an increase in the intracellular Ca^2+^ concentration. The signaling events downstream of Ca^2+^ can again be linked to diverse output, such as the secretion or the production of a therapeutic protein. By modularly exchanging the receptors and the output elements, any specific input can be wired via central Ca^2+^ signaling to any specific output [251,[286], [287], [288], [289]]. Similar central hubs for connecting a specific input to a specific output are canonical signaling cascades such as the JAK/STAT, MAPK, PLCG, or PI3K/Akt pathways [[290], [291]]. To extend sensing/input capabilities beyond naturally occurring receptors, the same group developed a platform for the engineering of receptors recognizing specific, user-defined targets. This platform relied on single-chain variable fragments (scFvs) of antibodies, dimerizing proteins, or other proteins with specific binding activity linked to the extracellular domains of erythropoietin receptor yielding customizable epitope sensors [291]. The further translation of such approaches into the clinics will depend on the validation of cell encapsulation strategies in clinical studies aiming at long-term implant survival and maximum safety with regard to undesired cell release. A recent study described a silicone reservoir with a porous (pore size <0.8 μm) polymeric membrane for the encapsulation and implantation of cells [292]. This setting, combined with a synthetic polymer coating, prevented fibrosis and sustained the survival of engineered encapsulated cells and the production of erythropoietin in immunocompetent mice for >130 days. The system was further retrievable after use for safe disposal [292].

#### 3.3.6 ELMs in robotics

Robots are taking over more and more tasks that previously required human intervention. Conventional robots are made from rigid materials, which allow them to perform rapid, precise, and sequential operations [295]. In recent years, more and more material scientists working in the robotic field drew inspiration from biology. Natural systems can execute tasks such as manipulation and locomotion without the need for a rigid skeleton. Moreover, living organisms are capable of sensing, actuation, locomotion, and most importantly, self-healing and self-regeneration. Engineers have thus explored the use of soft, nature-mimicking materials to confer the necessary deformability, adaptability, and agility to a new generation of robots, so-called soft robots [296,297]. One highly attractive field in soft robotics is the incorporation of living cells as sensors or actuators. Biohybrid robots can, for example, undertake biomimetic motions such as swimming [298], walking [299], or gripping [300]. They can further be controlled via optical [299], chemical [300], electrical, or magnetic [301] stimulation. In such soft biohybrid robots, living structures are combined with soft synthetic materials to perform the overall desired task. Here, PDMS is the most commonly used synthetic material because of its easy processability, adjustable stiffness, transparency, and modifiable surface [302]. Furthermore, hydrogels are also attractive candidates for the construction of biohybrid robots because of their excellent biocompatibility and an adjustable elastic modulus in the range of soft tissues allowing easier integration with living systems [303]. Also, other materials such as carbon-based nanotubes [304] or gold nanowires [305] have been applied. To better structure and describe such cell-incorporating soft robots, the Quinn group proposed a robotic taxonomic key for devices using organic materials [306]. This taxonomy has recently been revised by the Selhuber-Unkel group systematizing the robots with regard to structure, actuation, sensing, and control [305] (see also there for an extensive review of robots incorporating living materials).

Synthetic biology approaches have recently fostered advances in soft robotics by designing cell-based sensors and actuators. As actuators, especially in a liquid or biological environment, engineered muscle cells, both cardiac and skeletal ones, are attractive tools. Cardiac muscle cells have the advantage of spontaneous contractility and synchronized beating as long as glucose is provided in their environment. Thus, no external stimulation is necessary, yet the cells have to be applied on a material allowing for cell–cell connections (gap junctions) to mediate cell-to-cell excitation propagation. Consequently, cardiomyocytes have been used as autonomous biohybrid actuators [305,307]. Skeletal muscle cells, on the contrary, do not spontaneously contract but require external stimuli for inducing contraction. However, skeletal muscles have the ability to regenerate after injury, in opposition to cardiomyocytes [305].

**Fig. 23:**
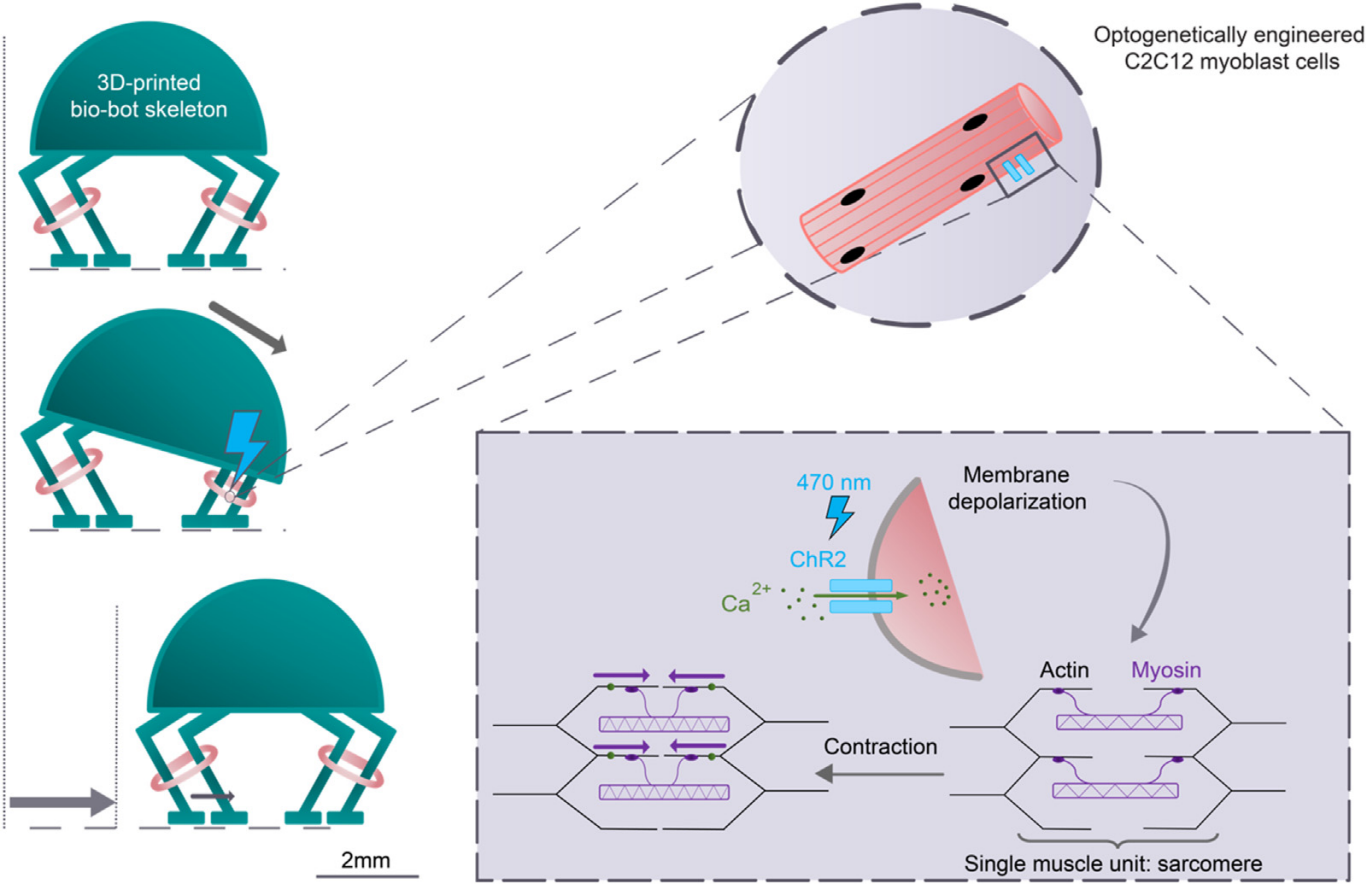
**Optically stimulated 2D locomotion of a biobot with a symmetrical structure.** A biobot skeleton was 3D printed and functionalized with muscle rings. The muscle ring, produced from engineered C2C12 murine myoblasts, further contained the blue light–sensitive ion channel channelrhodopsin 2 (ChR2). Upon local blue light illumination, ChR opened, leading to Ca^2+^ influx, the subsequent actin and myosin-mediated contraction of the sarcomere, and hence of the complete muscle. Optically stimulating contraction only on one site triggered targeted locomotion of the biobot [299].

To allow for external control of muscle cell contraction, cells were engineered to express the blue light–sensitive ion channel channelrhodopsin-2 (ChR2). Upon illumination with blue 470 nm light, the membrane depolarized leading to an increase in intracellular Ca^2+^ via ChR2. The calcium ions subsequently promoted contraction. This principle has been applied by the Parker group for constructing a soft-robotic ray with phototactic guidance [308]. To this aim, a ray-inspired gold-based skeleton was combined with an elastomer body that served as a substrate for the cultivation of rat cardiomyocytes engineered to express ChR2. The robot was operated in a physiological salt solution supplemented with glucose as an energy source. Simultaneous illumination on the left- and right-side induced cell contraction, which translated into an undulating movement of the elastomer and the subsequent propulsion to swim forward. Illumination of only one side triggered unilateral propulsion and a curved swimming path of the robot. Thus, maneuvering the robot through a labyrinth was possible by side-selective illumination [308]. In a similar approach, the Bashir group developed a biobot for optically stimulated 2D locomotion [299]. In this study, immortalized C2C12 murine myoblasts were engineered for ChR2-based light-inducible contraction. The use of these cell lines obviated the need for the repetitive isolation of primary cardiomyocytes from animal donors. The engineered cells were mixed with extracellular matrix constituents such as Matrigel basement membrane, fibrinogen, and thrombin and injected into a printed ring mold as a template for the formation of the muscle ring. Traction forces exerted by embedded cells drove self-assembly into densely packed muscle strips and rings. The resulting rings were able to exert 300 μN (0.56 kPa) of the active tension force in response to an optical stimulus. The muscle rings were attached to a two-leg-based biobot skeleton. Upon optical stimulation, the biobot started walking at a speed of 310 μm/s or 1.3 body lengths/min (Fig. 23) [299]. One drawback of biobots actuated by mammalian cells is the vulnerability to drying out in the air. This drawback was mitigated by developing a bio-robot in which the skeletal muscle cells have been embedded in a collagen capsule. This robot was successfully operated in the air [309].

Engineered microorganisms are also highly attractive components of soft robotic systems. Based on the ability of flagellated bacteria to actively swim, bacteriobots have been designed. The Gullivan group engineered the bacterial chemotaxis system to be responsive to theophylline [310]. In this work, production of the chemotaxis-involved protein CheZ was placed under the control of a theophylline-responsive RNA aptamer. Thus, only in the presence of theophylline, cells were able to perform a run and tumble movement, whereas in its absence, only a tumble movement was observed. The engineered bacteria were shown to swim toward a theophylline source but not toward structurally similar molecules such as caffeine [310]. In a follow-up study, the Goulian group engineered ‘hitchhiking’ consortia of bacteriobots, where one population produced the attractant molecule for the other population. Thus, one population followed the other by chemotaxis [311]. Such bacteriobots could be engineered for carrying a payload toward their target. This has, for example, been evaluated by the construction of tumor-targeting bacteria carrying chemotherapeutic drugs [312].

In an elegant approach, the Wegner group recently engineered *E. coli* for the reversible pick-up and delivery of cargo [313]. To this aim, *E. coli* was engineered to produce the biotinylated biotin-acceptor peptide on its surface. The cells were subsequently coated with streptavidin followed by the addition of biotinylated PhyB. Furthermore, a PIF-derived protein was fused to a His-tag for coupling to NTA-functionalized beads. Upon illumination with red light, the PIF-linked cargo was loaded via PhyB onto the *E.* coli microswimmers. However, upon illumination with far-red light, the proteins dissociated thus unloading the cargo [313]. Further examples of biologically powered robots are listed in Table 11 (see also [305] for a review covering the field).

**Table 11:** Engineered living materials for robotic applications.

Biological component	Control method	Robotic application	Reference
Skeletal muscle	Optical	Genetically modified skeletal muscle cells able to drive the movement of a biobot	[299]
Cardiomyocyte	Optical	Control on the speed and direction of a phototactic artificial ray	[308]
Neurons		Neuromuscular actuation of biohybrid biobots	[314]
*E. coli*	Optical	3D micromotors	[315]
*E. coli*	Chemical	Soft robotic gripper capable of fluidic actuation	[300]
*E. coli*	Magnetic	Erythrocyte-based bacteria swimmer with guidance provided by superparamagnetic iron oxide nanoparticles loaded into the erythrocytes	[316]
*Drosophila melanogaster* heart muscle	Optical	Light-regulated actuator	[317]

**Fig. 24:**
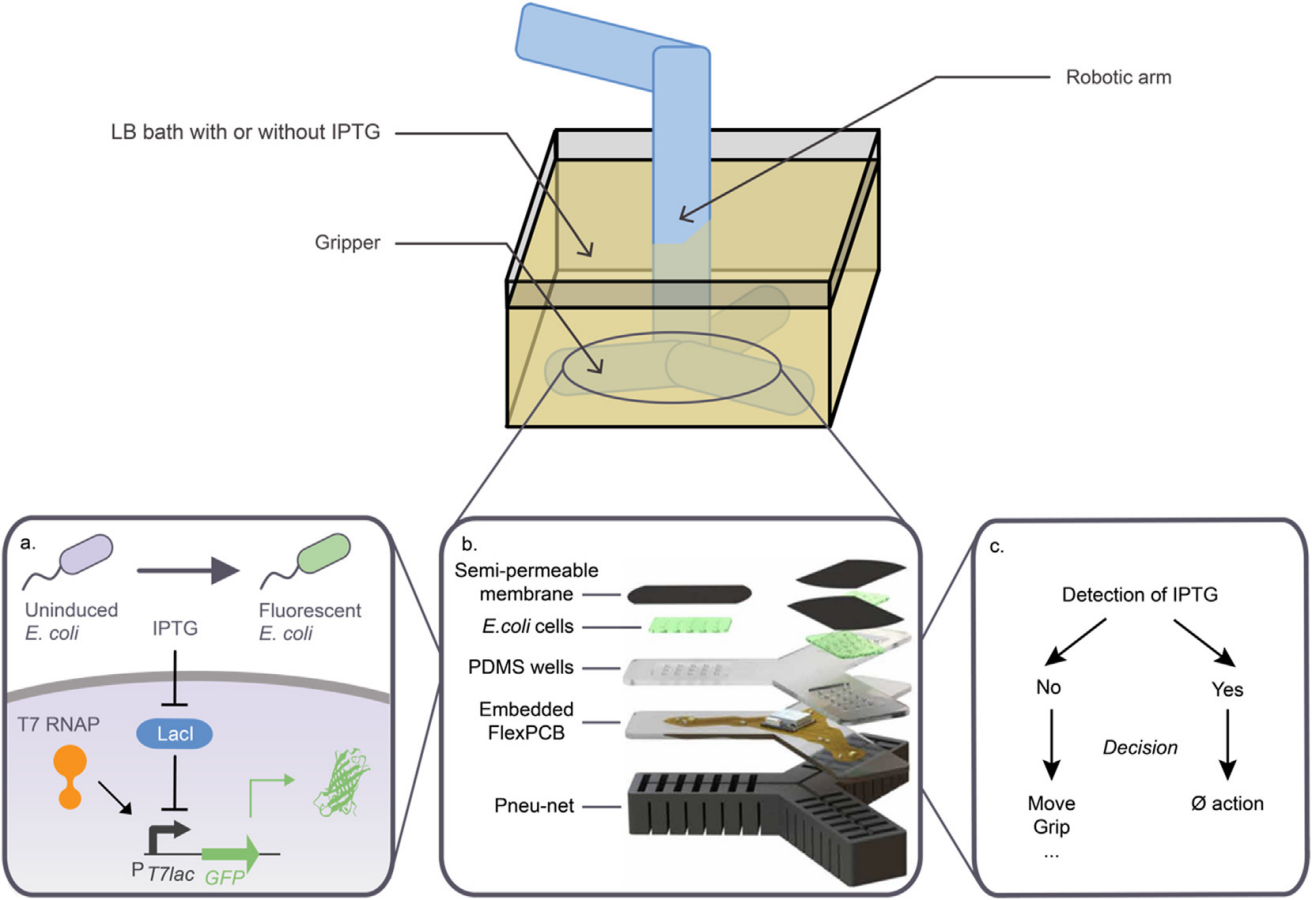
**A gripper robot with living sensors for chemical detection. a.** Construction of the biosensor. *E. coli* cells were genetically modified to produce the green fluorescent protein upon detection of IPTG. In this configuration, IPTG inhibited binding of the lactose repressor LacI to an RNA polymerase T7-specific promoter driving *gfp* expression. **b.** Construction of the gripper. The biosensor cells were embedded in a flexible gripper further featuring an optical system incorporated on a flexible printed circuit board (PCB) for the detection of GFP production. From Science Robotics 26, Vol. 4, Issue 31, eaax0765. Reprinted with permission from AAAS. **c.** Decision-making based on the biosensor readout. The gripper moved to the target vessel submersing the cells in the sensor. In the case of IPTG in the liquid, the biosensor produced a fluorescent readout that was detected by the electronic circuit, which further instructed the robot to not place an item into this IPTG-containing vessel. However, in case the target vessel did not contain IPTG, the robot placed the item into the vessel. IPTG, isopropyl β-d-1-thiogalactopryanoside [300].

Engineered microorganisms are also suitable as a living sensing component in robotic devices. For example, the Tan group constructed a gripper robot with engineered *E. coli* as a sensor [300]. The robot was programmed to sense the presence of the chemical IPTG in a solution and to place an item of choice into this solution only in the absence of the chemical. To this aim, *E. coli* was engineered to produce the green fluorescent protein under the control of an IPTG-inducible promoter. The cells were embedded into a PDMS-based device further equipped with LEDs and a photodetector to measure the cells’ fluorescence. Using this biohybrid sensor, the robot was able to probe the presence of IPTG in a liquid to decide whether or not to place an item into the liquid (Fig. 24) [300]. The broad availability of molecular sensors in synthetic biology makes this design principle for robots capable of sensing a large array of chemicals and biomolecules.

Other interesting organisms for the synthetic biology-based construction of robots are insects. Insects can freely move and are robust to fluctuating conditions in the environment. To the best of our knowledge, insect-based biohybrid robots have not yet been developed in combination with synthetic biology. However, interesting approaches in this direction comprise the loading of a biofuel cell to cockroaches for powering remote wireless devices for the transmission of hygrothermal data [318]. Moreover, thanks to the rapidly developing technologies to genetically engineer different insects, by, for example, the Alphey group and the company Oxitec, the design of insect-based biohybrid robots is likely to become a reality [319].

In an intriguing approach, the Ware group recently engineered living, shape-morphing ELMs that could potentially be used in soft robotics [320]. In this study, *S. cerevisiae* was embedded into hydrogels synthesized from the polymerization of acrylamide and *N*,*N*′-methylene bis-acrylamide. Cell proliferation within the hydrogel triggered an increase in the material's volume. To enable shape-morphing properties, the yeast cells were engineered with a blue light–responsive optogenetic switch to control the expression of the *his3* gene required for growth in a histidine-free medium. In this configuration, the local illumination with blue light triggered *his3* expression and the subsequent growth of the illuminated cells. This growth translated into a local increase in the hydrogel volume and enabled the optically guided morphing of the hydrogel shape [320].

## 4 Conclusions and perspectives

In this review article, we have identified two areas in which synthetic biology is fueling innovation in materials sciences: (i) by providing structural and functional (precursors of) materials, and (ii) by engineering organisms in a way that they form or assemble the material or confer novel functionality to it while remaining an integral part of the resulting living composite. Here, we anticipate the future developments in these fields and identify new scientific directions as well as research policy measures to boost the flourishing of the field.

### 4.1 The promise of artificial intelligence and model-based design

The rapidly increasing capabilities of artificial intelligence (AI) and machine learning will transform the way biological systems are analyzed and engineered [321]. In recent years, the frequency of scientific publications applying AI to synthetic biology has rapidly increased. Here, we highlight the latest examples of AI-driven synthetic biology that have immediate consequences for the design of synthetic biology-based materials.

Key to any engineering is the ability to precisely predict and control the transcription and translation rate of proteins. Here, AI has recently fostered advances. For example, applying techniques from computer vision and natural language processing, deep learning architectures were developed to characterize and optimize RNA-based riboregulators for controlling gene expression [322], so-called toehold switches. The deep learning systems were, for example, applied through transfer-learning to redesign suboptimal toehold sensors and to improve their performance, even with sparse training data [323,324]. Such switches are now useful for paper-based systems and materials to sense specific input RNA stimuli and to drive gene expression [325]. Further examples of using AI include the prediction of ribosome-binding sites providing desired translation rates [326], the *de novo* design of promoter elements in *E. coli* [327], or the design of an optimized CRISPR guide RNA for high-fidelity Cas9 variants [328]. Such approaches to construct genetic tools with predictable functions are highly relevant for the design of more complex expression systems as required for synthetic gene networks [321] or for orchestrating complex metabolic fluxes [329,330]. Accordingly, AI approaches are expected to foster innovation in metabolic engineering by simplifying the reconstruction and design of metabolic pathways, optimizing pathways, building and testing cellular factories, as well as scaling up cellular factories (as recently reviewed elsewhere [331]). Such metabolic engineering widens the opportunity for the bio-based production of monomers or precursors of materials or to incorporate desired metabolic reactions (for example, drug production) into (therapeutic) ELMs.

Another field, where AI is currently pushing a breakthrough, and which has direct implications on the synthesis of synthetic biology-based materials, is the prediction of protein structure based on the amino acid sequence. A major breakthrough was the development of AlphaFold, a trained neural network to make accurate predictions of protein structure [144]. The performance of AlphaFold was strongly increased in AlphaFold2 as seen in a competition in late 2020. AlphaFold2 had been trained on approx. 170,000 protein structures from the protein data bank. The system was able to determine highly accurate structures with an average error of approximately 1.6 Å. Furthermore, AlphaFold2 estimated which parts of each predicted protein structure were reliable using an internal confidence measure [144,332]. Such tools will enable the *in silico* design of proteins with desired structures opening new possibilities for designing structural and functional building blocks for protein-based materials. In a complementary approach to predicting protein structures, AI was applied to design icosahedral protein-based nanostructures at the example of the adeno-associated virus (AAV) capsid. In this approach, machine learning models were trained on experimental data without biophysical modeling. The trained models were used to design icosahedral capsids based on highly diverse protein sequences. In total, 201,426 variants were predicted of which 110,689 were experimentally verified to be viable as AAV capsid [333]. This approach unlocked vast areas of functional but previously unreachable sequence space, providing new opportunities, not only for viral vector design but also in general for the design of protein-based (nano-) materials.

An intriguing application of AI was recently described by the You group facing the challenge of excessive computation time when running their models to guide the design of synthetic biological pattern-forming processes [334]. In a first step, they applied a conventional model based on partial differential equations to simulate synthetic pattern formation using different parameter combinations. In the next step, these data were used to train a neural network, which was finally applied to predict the outcome of the patterning based on different parameters. The authors showed that the predictions of the neural network closely matched the ones from the mechanistic model, however, with a 30,000 times improved speed [334].

In addition to such AI approaches, the design of synthetic biological systems is heavily supported by quantitative mathematical models to identify the parameter space in which the final system shows desired properties [14]. Using quantitative models, different parameter combinations can easily be tested and optimized, thus significantly increasing the chances of success when building and testing the device in the wet lab [184,335,336]. Modeling approaches used in synthetic biology have also successfully been applied for guiding the synthesis of synthetic biology-based information-processing materials systems functioning as a signal amplifier or counter of input pulses [156,165,166]. However, when designing ELMs, where the biological processes in the cell are directly linked to the macroscopic properties and functions of the material, novel modeling strategies would be required. Combining the modeling approaches established in synthetic biology with those in polymer physics could be a promising strategy. Such integrated modeling could, for example, be used to predict the materials' properties based on the cell's engineered genetic and metabolic programs and would in turn shortcut the design route to tailored ELMs.

The power of quantitative mathematical models and AI-based prediction tools can further be potentiated by the use of robotic machinery for the automated generation of microbial strains and the subsequent testing for performance. Thus, biosynthetic and genetic networks for synthesizing complete materials or precursors thereof could be designed, built, tested, and optimized within an integrated, automated setup [25,330,337,338].

Given the huge opportunities of data-driven and IT-intensive synthetic biology, it is no surprise that major IT companies are entering the synthetic biology field. For example, Google and its subsidiary DeepMind are front-running the field in protein structure prediction (AlphaFold) or in the design of multiprotein assemblies such as AAV capsids (see above). Furthermore, Microsoft recently incorporated its first molecular biology laboratory (Microsoft Research Station B [339,340]) to build a platform for programming biology and thus enabling fundamental breakthroughs across a broad range of industries, including medicine, agriculture, food, construction, textiles, materials, and chemicals [339]. Similarly, IBM Research has established the Cellular Engineering Laboratory aiming at establishing end-to-end engineering principles for biological systems. Using mathematical models and data-driven machine learning approaches, IBM aims at extracting, inferring, and establishing design principles for biological machines [341].

### 4.2 Toward the directed evolution of materials

The properties of materials in nature are the result of a billion-year-long trial and error process in which living organisms gradually evolved the material's ‘synthesis protocol.’ This Darwinian principle of mutagenesis and selection has successfully been reconstructed in the laboratory for the directed evolution of biomolecules with desired function. Directed evolution comprises four steps that are iteratively applied [342]: (i) Diversification of the starting material. To create the desired variety, the gene encoding the desired product such as an antibody or an enzyme is subjected to mutagenesis. The resulting library of diversified genetic components is subjected to (ii) transcription and translation in which the proteins encoded by the library are biosynthesized. This library of proteins undergoes (iii) screening and selection so that only those variants are retained that match the desired properties. In the last step (iv), which is replication, the successful variants are amplified. The four steps can be applied iteratively to converge to optimized variants [342]. From this process, it is evident that directed evolution requires a bidirectional coupling of genotype and phenotype: the forward coupling of genotype to phenotype takes place in the transcription/translation step, where the genetic information is converted into the corresponding protein. In the screening/selection step, however, it must be ensured that the selection at the protein/phenotype level allows the inference of the genotype so that only those gene variants are retained that encode the selected proteins. Different approaches have been developed for ensuring such bidirectional coupling of genotype and phenotype such as phage display, ribosome display, or the selection of enzymes in microorganisms where the desired enzyme activity would allow survival/identification of the producer cell (for reviews see Refs. [[343], [344], [345], [346]]).

The main limitation for the directed evolution of materials is the need to bidirectionally couple the genotype of the material producer and the phenotype of the final material [347]. One way to overcome this limitation would be a combination of ELMs with high-throughput materials characterization. For example, ELM-producing micro-organisms could be mutated and cultivated in a format where each cell would produce one distinct ELM [348] that could subsequently be screened for desired materials properties using high-throughput robotics for measuring, for example, rheological properties [349]. The establishment of such directed evolution would allow evolving materials synthesized from renewable sources toward any property or function that can be synthesized by any engineered cell or multicellular community.

### 4.3 Harnessing nature's ability for self-organization

The synthesis of structured materials in nature occurs in a self-organized manner yielding hierarchical architectures [4]. By engineering such pattern-forming processes into ELMs, living materials could form in a self-organized manner. A possible starting point for such work is recent efforts in synthetic biology aiming at engineering pattern-forming processes. In a pioneering work, the Lim group used synthetic Notch-based receptors for juxtacrine signaling to engineer artificial genetic programs in which specific cell-to-cell contacts induced changes in cell adhesion [350]. These minimal intercellular programs yielded assemblies with hallmarks of natural developmental systems such as robust self-organization into multidomain structures, well-choreographed sequential assembly, cell type divergence, symmetry breaking, and the capacity for regeneration upon injury [350]. In an alternative approach, the Weiss group established self-forming stochastic Turing patterns in a synthetic bacterial population [351]. Complementary to such self-organized pattern formation, the emergence of patterns by providing spatiotemporally controlled external cues was shown in bacterial as well as mammalian systems. For example, based on quorum-sensing signaling, the Weiss group developed a synthetic multicellular system for programmed pattern formation in *E. coli* [352]. Similarly, the Lim group recently described a mammalian synthetic system based on fluorescent proteins as morphogens to program multicellular patterning [353] (for review articles on pattern formation in synthetic biology, see Refs. [354,355]).

Initial work in engineering pattern formation into ELMs has been pioneered by the Lu group who programmed bacteria to form a tunable multiscale material. In this configuration, *E. coli* communicated via quorum-sensing molecules, where the local concentration of the signaling molecule induced production of different CsgA variants yielding material production and patterning [212]. Similarly, the Riedel-Kruse group engineered bacterial adhesion for programming multicellular morphologies and patterns [191]. Further research on spontaneous or programmed pattern formation in ELMs will allow the synthesis of more and more sophisticated multicomponent, hierarchically structured materials. One intriguing feature of such materials structured by pattern-forming processes is the ability of self-regeneration and self-healing. Upon local detection of an injury-induced change in the reaction-diffusion system, regenerative programs can automatically be triggered.

### 4.4 Translation of synthetic biology-based materials toward application

The translation of synthetic biology-based materials toward application requires two main steps: (i) up-scaling the production process to industrial scale; and (ii) as a function of the application field, obtaining regulatory approval. Both steps differ fundamentally for the two areas described in this manuscript, non-living materials produced by engineered cells and ELMs.

For promoting non-living materials produced by engineered cells toward application, conventional and approved industrial scale processes from biological, chemical, and materials engineering can be applied. For example, in the case of precursors for polymer materials (e.g. nylon), the precursors are produced by standard fermentation and subsequently processed using the same chemistry and processes as for conventional polymer materials. For protein-based materials, standard production and purification processes are applicable, whereas the transformation to the final product may require specific processing. This is, for example, the case for silk-based materials, where the way of processing strongly influences the spatial arrangement of the protein fibers and the resulting macroscopic properties of the material [86,87]. From a regulatory point of view, products produced by genetically engineered organisms, where the organism is not part of the final product, are considered as unproblematic with regard to genetic engineering. For example, food products processed with the help of genetically modified organisms (e.g. cheese production with recombinantly produced rennet/chymosin) require no genetic engineering-specific labeling (Regulation (EC) No 1829/2003 of the European Parliament and of the Council).

In recent years, several companies started developing materials produced or processed by synthetic biology tools. For example, the company ColoriFix is using a synthetic biology approach to produce, deposit, and fix pigments onto textiles. This method could save up to 90% of water in the dying process and reduces the released dyes by 99% [356,357]. This is suggested as an environmentally friendly alternative to chemical dying being responsible for around 20% of global industrial water pollution [357]. The company Modern Meadow is bringing the potential of synthetic biology to the fashion industry. They engineered yeast to produce and shape engineered collagen to emulate animal hides and may have even properties superior to natural leather [358,359]. Similarly, the company Modern Synthesis [360] is using bacterial cellulose produced from engineered bacteria to design clothing. As a special feature, they use engineered organisms producing dye molecules to color the fabric during the synthesis process. In an application for the electronics industry, Zymergen developed a process for producing diamine monomers from engineered organisms that have been generated by a suite of robotics to build millions of strains in parallel, with AI learning from failures to design the next round of strains. The diamines are further processed to hyaline, a polyimide film for use in flexible electronics such as foldable smartphones [48].

In contrast to the aforementioned non-living materials, the recently emerging class of ELMs needs to overcome challenges with regard to manufacturing and regulatory approval.

Especially for ELMs formed or assembled by engineered organisms, new production processes are required as a significant step of the process is taken over by engineered cells and no longer by the materials engineer. Thus, conditions must be provided, where the materials can grow to desired structure and shape. This can be obtained by 3D printing, casting, or molding. For example, the company Biomason is growing tiles as a construction material using molds [4] (for an overview of processing methods for ELMs, see Ref. [4]). One strong advantage in ELM production is the possibility to use local resources (e.g. sand), to inoculate those with the engineered organism, and to grow the material *in situ*. This is of special interest for production processes in remote areas without existing infrastructure or in space [236,335] travel. Examples, where ELMs are already used in a commercial context, comprise the company Glowee that is engineering microorganisms to make them more efficient in terms of light production. Based on engineered bioluminescent bacteria, Glowee is producing illumination hardware for different design and commodity purposes [361]. In the construction field, the company Biomason is growing tiles, whereas the company Basilisk Concrete is using bacteria to prepare self-healing concrete [238,362]. Also, living materials can serve as inspiration for art and design. The designer Jan Klingler is producing lamps with a colored pattern formed by micro-organisms [363]. Although these organisms have not been genetically engineered, here also synthetic biology might allow growing aesthetic colors and patterns [354,355].

As opposed to the aforementioned applications, the introduction of synthetic biology-based materials into human therapy faces strict regulations to ensure patient benefit. In these evaluations, the observed therapeutic effect is compared to possible risks and side-effects. For implanted living therapeutic materials, however, the benefit/risk ratio might change over time, for example, because of changes in cell viability or number. This requires new ways to assess patient benefit and possible side-effects that must be consolidated in the regulation process of such novel therapeutics.

For the therapeutic application of materials containing engineered cells, especially if the material is to be implanted in the body, materials are required that (i) allow sufficient long-term viability of the cells; (ii) allow the cells to sense physiological and pathological parameters in the body or to react to external (optical) stimuli; (iii) shield the cell from the body's immune response to avoid rejection; (iv) show good biocompatibility; (v) withstand the mechanical forces in the body; (vi) ensure no unintended release of the engineered cells; and (vii) allow easy, patient compliant administration and removal, for example for replenishing the therapeutic cells. In this context, stretchable hydrogel-elastomer-based hybrid materials have shown promising results in initial laboratory studies [247]. For example, the recently developed deployable physical containment strategy by the Lu group is based on encapsulating engineered bacteria into alginate/Ca^2+^ beads that were further coated with a tough hydrogel formed from crosslinked alginate and polyacrylamide. They showed that this strategy effectively prevented the escape of bacteria [364].

Based on regulatory requirements, we anticipate that first applications containing engineered, living cells will be applied topically, for example, as wound dressing, where the material can easily be applied and removed in case complications manifest. Further applications might be living materials targeted to the digestive tract such as ELMs or biohybrid devices based on engineered probiotic bacteria. Such devices will be transiently in the body and eliminated in the course of a few days. One strong advantage of (probiotic) bacteria-containing materials is, that in case of complications, systemic antibiotic therapy can be applied to efficiently eliminate the living therapeutic payload.

In contrast to topically or gastrointestinally targeted materials containing engineered cells, the application of encapsulated cells that are to be implanted into the body, likely faces stronger regulatory requirements. To get a glimpse of the time scales that might be necessary for such approaches, other similarly innovative therapies might serve as examples. For example, gene therapy trials using retroviral viral vectors have been performed around the beginning of the millennium until facing an unexpected setback in 2002/2003 by the observation that the applied viral vectors triggered insertional oncogenesis and T cell leukemia in patients [[365], [366], [367], [368]]. In subsequent years, much effort was invested in improving the safety of the vectors, which finally yielded the regulatory approval of lentiviral-based CAR-T therapies (Kymriah® and Tecartus™) in 2018. These therapies are now providing a life-saving opportunity for leukemia patients being unresponsive to any alternative therapy. We anticipate that the regulatory approval of implantable materials containing engineered cells will require extended preclinical and clinical trial phases in which unexpected effects might be observed and overcome. Given the example of gene therapy, it is also likely that therapies targeting a high unmet medical need, such as untreatable life-threatening diseases, will receive prioritized attention and approval.

Once these regulatory steps are overcome and initially approved therapies show safety and efficacy, the way for a new generation of materials containing engineered cells will be paved. The introduction of such materials will likely revolutionize the way how health and disease are managed. A glimpse of what could become clinical reality can be gained in recent review articles summarizing medically oriented pioneering work in synthetic biology [23,369].

### 4.5 Structural and funding policies to harness the potential of synthetic biology-based materials

Research and development of materials based on synthetic biology is an inherently interdisciplinary enterprise requiring the close collaboration of materials scientists and synthetic biologists. In such collaboration, it is essential that each partner learns the needs, opportunities, and constraints of the other to efficiently organize the overall process. Such intrinsic need for interdisciplinary collaboration is reminiscent of the emergence of synthetic biology, where engineers and life scientists teamed up to rethink the way of how biological systems with desired function could be constructed. Likely the most efficient way of fostering interdisciplinary collaboration is to bring the different partners into close proximity in a dedicated research center. This interdisciplinary center concept has been implemented, for example, in the Wyss Institute at Harvard or in the Department of Biosystems Science and Engineering of ETH Zurich in Basel, both of which emerged as major innovation hubs in synthetic biology. With these successful examples in mind, centers uniting all complementary skills for advancing synthetic biology-based materials can be expected to become major drivers in the materials sciences. Such targeted, center-based initiatives uniting synthetic biology and materials science have been initiated, for example, at the Wyss Institute, at the Leibniz Science Campus for Living Therapeutic Materials [370], or with the ELMs initiative at the UC San Diego Materials Research Science and Engineering Center [371].

The highly interdisciplinary nature of research on synthetic biology-based materials means that they are challenging to realize through traditional, discipline-specific funding schemes. However, creating specific grant opportunities for this promising area of research accelerates the field and enables innovation that would otherwise has remained untapped. For example, the U.S. Defense Advanced Research Project Agency (DARPA) was early to identify the innovative potential of ELMs and initiated a specific funding program [372]. DARPA supported research groups and companies developing ELMs as living building materials [78,373]. Also, the U.S. National Science Foundation specifically funds research on ELMs in the frame of its prestigious Idea Machine program. These dedicated funding schemes for ELMs will give the U.S. a competitive advantage in the field. Other national and private funding instruments should follow to secure a share of the innovation potential of synthetic biology-based materials.

Equally important as dedicated centers and funding schemes, the future success of synthetic biology-based materials requires a strong, collaborative community that jointly advances scientific discoveries, supports scientific exchange, and also increases the visibility toward funders and the broader public. To support this community-building, a new Living Materials conference series was established in 2020 [374].

By providing a means to instruct and engineer nature's own materials scientist — the individual cell — synthetic biology has the power to drive the biologization of the materials sciences and deliver materials needed to address major global challenges. Indeed, the rapidly emerging field of synthetic biology-based materials has already produced innovative materials with remarkable functionalities for a variety of applications in areas ranging from sustainable bioeconomy to healthcare. Synthetic biology continues to progress at a rapid pace, leading to an ever-growing capability to instruct and engineer cellular processes. We predict that the growing success and virtually unlimited possibilities of synthetic biology-based materials will autocatalytically accelerate research and innovation in this field and will usher in a new era of living materials.

## Declaration of competing interest

The authors declare that they have no known competing financial interests or personal relationships that could have appeared to influence the work reported in this paper.
